# High‐throughput quantitative assessment of ABA‐responsive elements at single‐nucleotide resolution

**DOI:** 10.1002/qub2.87

**Published:** 2025-01-30

**Authors:** Fangnan Sun, Yaxin Deng, Weihua Zhao, Yixue Xiong, Lingxia Zhao, Lida Zhang

**Affiliations:** ^1^ Department of Plant Science School of Agriculture and Biology Shanghai Jiao Tong University Shanghai China

**Keywords:** STARR‐seq, abscisic acid responsive element, *cis*‐regulatory element, enhancer, synthetic promoter

## Abstract

Abscisic acid (ABA)‐responsive elements (ABREs) are the major *cis*‐regulatory elements in ABA‐induced gene expression. However, the impact of sequence variations on ABRE function is not yet well‐understood. Here, we used synthetic STARR‐seq to quantitatively assess the effects of single‐nucleotide substitutions on ABRE activity. Our results revealed that the nucleotide substitutions in both the ACGT‐core and ACGT‐flank regions affected transcriptional strength. Alterations in the ACGT‐core sequence had a more significant impact on ABRE activity than changes in the flanking region. Interestingly, we observed that the ACGT‐flank variants with high activity exhibited a strong sequence preference in the downstream region, whereas the highly active core variants were diverse in sequence patterns. Our studies provide a quantitative map of ABRE activity at single‐nucleotide resolution, which will facilitate the design of ABA‐responsive promoters with desired activities in plants.

## 1 INTRODUCTION

Precise control of gene expression is essential for generation of transgenic plants with desired traits. *Cis*‐regulatory elements (CREs), such as enhancers and promoters, encode the DNA information required to establish precise patterns of gene expression during plant development and adaptation to environmental changes [1]. Sequence variations within CREs can alter their interactions with transcription factors and other transcriptional regulators, and lead to changes in gene expression, affecting the plant phenotypes [2]. Understanding the effects of sequence changes on CRE activity is crucial for the modification of regulatory DNA in plant engineering.

To directly measure CRE activity on a large scale, massively parallel reporter assays (MPRAs) have been developed in the past decade [3, 4, 5]. Although MPRAs allow the simultaneous testing of thousands of DNA sequences and their variants for regulatory activity in a single experiment, the coupling of candidate CREs with DNA barcodes complicates the generation of screening libraries [6, 7]. To overcome this limitation, a variant of MPRAs, self‐transcribing active regulatory region sequencing (STARR‐seq) was developed by using the CRE itself as a DNA barcode [8]. The STARR‐seq relied on the insertion of candidate CREs in the 3' untranslated region (3'UTR) of a reporter gene, which was scaled to identify millions of CREs at the whole‐genome level [9, 10, 11, 12, 13]. STARR‐seq has been applied to identify CREs in Arabidopsis, rice, and maize protoplasts [14, 15, 16, 17]. However, this method, which inserts candidate CREs in the 3'UTR suffers from a relatively low signal‐to‐noise ratio in plants [18]. Recent studies have shown that the candidate CREs are most effective when inserted upstream of the minimal promoter rather than within the 3'UTR [19, 20]. The improved version of STARR‐seq provided the ability to parallel identification and assessment of plant CREs by transient expression of reporter genes in tobacco leaves [21, 22].

The phytohormone abscisic acid (ABA) plays a pivotal role in a variety of developmental processes and adaptive responses to environmental stimuli in plants [23, 24, 25]. Promoter analysis of ABA‐regulated genes has led to the identification of ABA‐responsive elements (ABREs) for ABA‐inducible gene expression [26]. The ABREs contain a conserved ACGT sequence, which is the binding site for basic leucine zipper (bZIP) transcription factors [27]. Sequence variations within ABREs have been shown to affect gene expression by altering bZIP protein binding [26, 28, 29]. ABRE sequences exhibit diversity but are commonly found in the promoter regions of ABA‐responsive genes in plant genomes [30, 31, 32]. However, the precise effects of sequence variations on ABRE function are still not well‐understood.

In the study, we developed a synthetic version of STARR‐seq to systematically study how sequence variations in ABRE affect transcriptional activity. We generated the STARR‐seq libraries with synthetic ABRE variants, and assessed the regulatory activities of these elements in transiently transformed tobacco leaves. By a comprehensive analysis of ABRE variants, we identified sequence determinants of ABRE activity and uncovered regulatory characterization for ABA response. Our studies establish a promising approach for quantitatively evaluating ABRE activity at single‐nucleotide resolution. The resulting quantitative map of ABRE activity will facilitate design of ABA‐responsive promoters with desired features in plants.

## 2 RESULTS

### 2.1 Strategy for high‐throughput assessment of ABRE activity

**FIGURE 1:**
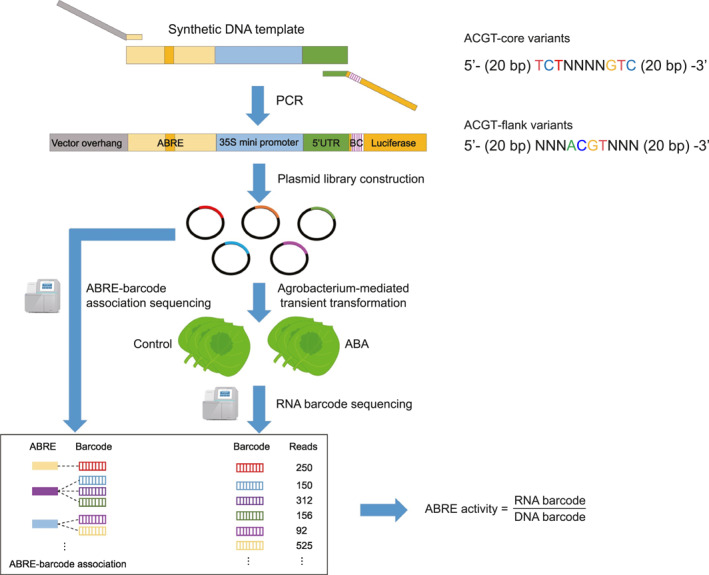
Schematic of the synthetic STARR‐seq method used to measure ABRE activity in tobacco leaves. The oligo pool containing degenerate nucleotides (N) within the ABRE element were synthesized and amplified using PCR to add the minimal promoter and the random barcodes. The amplified fragments were cloned into plasmid constructs to drive the expression of a luciferase reporter gene. The STARR‐seq libraries were transiently transformed into tobacco leaves, and the transcribed barcodes were counted by targeted RNA sequencing. The promoter strength was quantified by comparing the reporter transcript abundance with that of input plasmid.

To quantitatively assess the effects of nucleotide substitutions on ABRE activity, we established a synthetic STARR‐seq method for simultaneously testing the synthetic ABRE variants by transient expression of libraries in tobacco leaves. As shown in Figure 1, we synthesized an oligonucleotide pool of 50 base pair (bp) ABI1 promoters with randomized nucleotides in ABRE element. The synthesized oligo pool was amplified using PCR to add the 35S minimal promoter and the random barcodes. We introduced the barcodes in the reporter gene to link ABRE that enabled us to determine from which variant the reporter transcripts were driven. The amplified fragments were cloned into the vector to construct the STARR‐seq libraries. The resulting libraries were transiently transformed into tobacco leaves, and the transcribed barcodes were counted by targeted RNA sequencing (RNA‐seq). The transcriptional strength of the ABRE variants was quantified by measuring the reporter transcript abundance to the abundance of input plasmid.

We generated one library of ACGT‐core variants and four libraries of ACGT‐flank variants to assess the effects of all possible single‐nucleotide substitutions on ABRE activity. Additionally, a control library containing the 50‐bp enhancer (from −96 to −47 relative to the transcription start site, TSS) from the 35S promoter was also constructed in the STARR‐seq assay. By association sequencing of the pooled plasmid library, we assigned the unique barcodes to the ABRE variants that were cloned with in the random pairing design. The ACGT‐core library consisted of 255 variants, each with 11 unique barcodes, while the four ACGT‐flank libraries contained a total of 3491 variants, with an average of approximately 10.8 barcodes per variant (Figure S1). To enhance the readouts of STARR‐seq, only ABRE variants linked to at least 5 unique barcodes were kept for further analyses. By RNA sequencing of barcoded reporter transcripts, a total of 2459 unique barcodes associated with 220 ACGT‐core variants and 32,885 barcodes linked to 2334 ACGT‐flank variants were recovered from the RNA‐seq samples, respectively (Table 1).

**TABLE 1:** Summary of STARR‐seq libraries.

Library	All possible variants	Barcodes detected by DNA‐seq	Associated variants (%)^a^	Barcodes recovered by RNA‐seq (%)^b^	Variants detected by RNA‐seq (%)^c^
35S	1	108	1 (100.0%)	102 (94.4%)	1 (100.0%)
ACGT‐core	256	2805	255 (99.6%)	2459 (87.7%)	220 (86.3%)
ACGT‐flank	4096	37,842	3491 (85.2%)	32,885 (86.9%)	2334 (66.9%)

^a^ The number of associated variants/number of all possible variants.

^b^ The number of barcodes recovered by RNA‐seq/number of barcodes detected by DNA‐seq.

^c^ The number of variants detected by RNA‐seq/number of associated variants.

**FIGURE 2:**
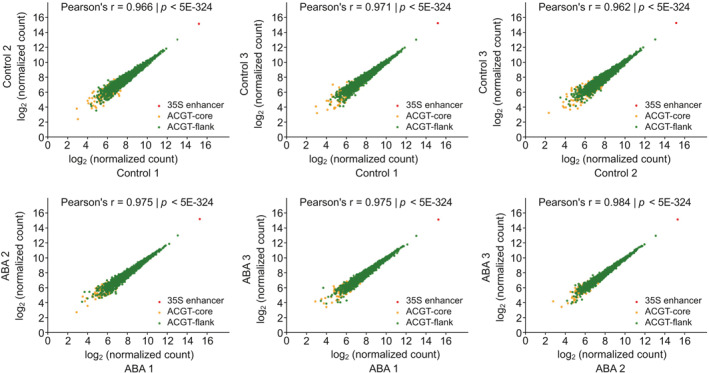
Correlation plots for biological replicates of STARR‐seq in tobacco leaves. Pearson correlation coefficient and *p* value are indicated for each pair of RNA‐seq replicates.

**FIGURE 3:**
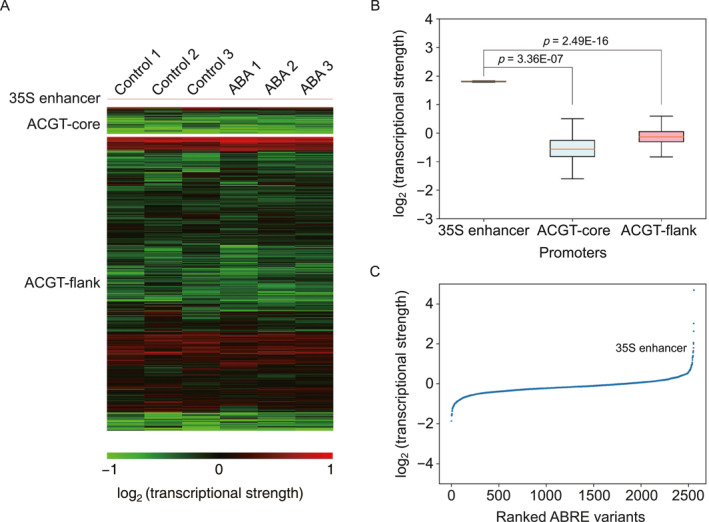
Promoter strength identified by STARR‐seq. (A) Heat map of promoter strengths in tobacco leaves. (B) Comparison of transcriptional strengths of the enhancer (from −96 to −47 relative to the TSS) from the 35S promoter, ACGT‐core, and ACGT‐flank libraries in tobacco leaves. (C) Promoters ranked by transcriptional strength.

We performed three biological replicates for each condition in the STARR‐seq experiment. The replicates were strongly correlated (Pearson correlation coefficient, PCC = 0.945 for control, and PCC = 0.975 for ABA treatment), indicating highly reproducible for the STARR‐seq assay (Figure 2). The RNA‐seq analysis revealed that the regulatory architecture of a single copy of the ABRE variant in conjunction with the 35S minimal promoter could drive the expression of the reporter gene in tobacco leaves (Figure 3A). As expected, the activity of the 50‐bp enhancer from the 35S promoter was significantly higher than the ACGT‐flank variants whereas the activity of the ACGT‐core variants was much lower (Figure 3B). Promoters ranked by their transcriptional strength (RNA/input DNA ratio) revealed that only five ABRE variants had higher transcriptional activity than the 35S enhancer, representing 0.2% of ABRE variants tested in the STARR‐seq assay (Figure 3C). Taken together, our results suggested that the STARR‐seq method produced reproducible and quantitative information, which could be used for systematically evaluating the effects of sequence variations on ABRE activity.

### 2.2 Effect of ACGT‐core variation

**FIGURE 4:**
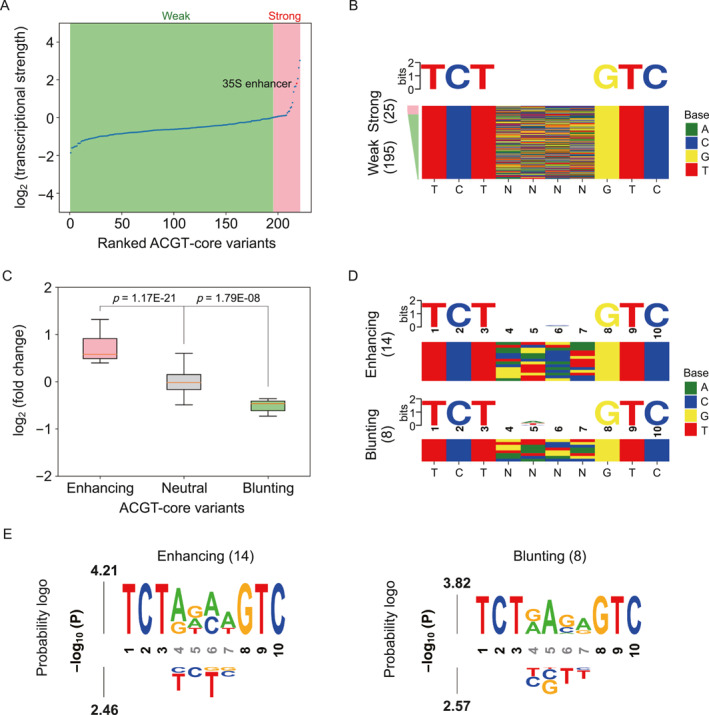
Analysis of ACGT‐core variants. (A) ACGT‐core variants ranked by transcriptional strength. (B) Consensus sequence (upper) for the strong ACGT‐core variants and a color chart (below) summarizing the sequence at each position for the ranked core variants. (C) Boxplot of the log_2_ fold change in the activity for enhancing neutral and blunting ACGT‐core variants under ABA treatment. (D) Consensus motifs (upper) and color charts (below) summarizing the sequence at each position for enhancing and blunting core variants. (E) Sequence probability logo for enhancing and blunting core variants from statistical tests of whether a specific nucleotide at a given position is more (upper) or less (below) active than other variants. Fixed positions in black.

The ABRE elements contain an ACGT‐core usually found in binding sites for bZIP proteins. An STARR‐seq library was generated with core variants within the ABRE element to assess the effects of these sequence variations on transcriptional activity. Compared to the ACGT‐flank variants, changes in the core region had a more pronounced effect on ABRE activity (Figure 3B). Promoters arranged in order of transcriptional strength showed that 25 (11.4%) core variants exhibited high activity with a log_2_ RNA/DNA ratio > 0, while 195 (88.6%) variants displayed low activity with a log_2_ RNA/DNA ratio < 0 (Figure 4A). We created a sequence logo from the highly active core variants, but it did not reveal any strong sequence preference for these variants (Figure 4B). These results suggested that the core variants exhibited a diverse sequence pattern in enhancing transcriptional activity.

To analyze the responsive activity of each core variant, we used DESeq2 to compare the abundance of reporter transcripts between control and ABA‐treated leaves [33]. This resulted in the identification of 14 (6.4%) core variants with significantly increased activity, 8 (3.6%) variants with significantly decreased activity, and 198 (90.0%) that did not exhibit significant changes in transcriptional activity under ABA treatment (Figure 4C, Figure S2). We observed a subtle sequence preference for both the enhancing and blunting core variants under ABA treatment (Figure 4D). Interestingly, the sequence logo indicated that the core variants with highly responsive activity were preferentially associated with an AGAA sequence (Figure 4E). The results suggested that some alternative core motifs like AGAA are also able to enhance the ABA‐responsive activity in plants.

### 2.3 Effect of ACGT‐flank variation

**FIGURE 5:**
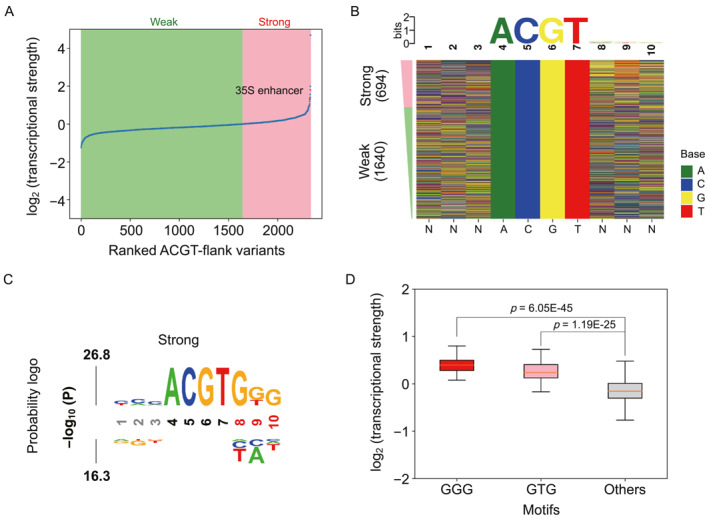
Analysis of transcriptional strength of ACGT‐flank variants. (A) ACGT‐flank variants ranked by transcriptional strength. (B) Consensus sequence (upper) for the strong ACGT‐flank variants and a color chart (lower) for the ranked ACGT‐flank variants. (C) Probability logo for ACGT‐flank variants. Positions with significant nucleotides (*p* < 0.01) are highlighted in red, and fixed positions in black. (D) Boxplot of promoter strength for the motifs GGG, GTG and other sequences in the downstream flanking region.

The ACGT‐core sequence within the ABRE element is well conserved, but the flanking sequences beyond the core vary. We next constructed four STARR‐seq libraries to assess the effects of flanking sequences on ABRE activity. Transient expression of libraries revealed that 694 (29.7%) ACGT‐flank variants exhibited high activity with a log_2_ RNA/DNA ratio >0, while 1640 (70.3%) variants showed low activity with a log_2_ RNA/DNA ratio <0 (Figure 5A). We observed a significant sequence preference for the highly active ACGT‐flank variants (Figure 5B,C). In particular, the transcriptional activity was significantly higher for variants with GGG or GTG in the downstream region (Figure 5D).

**FIGURE 6:**
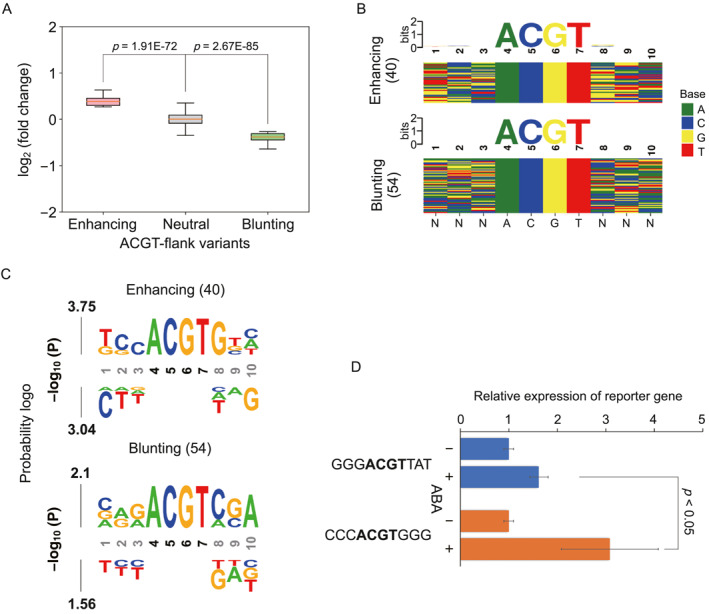
Analysis of responsive activity of ACGT‐flank variants. (A) Boxplot of the log_2_ fold change in the activity for enhancing neutral and blunting ACGT‐flank variants under ABA treatment. (B) Consensus motifs (upper) and color charts (below) for enhancing and blunting ACGT‐flank variants. (C) Probability logo for enhancing and blunting ACGT‐flank variants. Fixed positions in black. (D) Transcriptional strength of two ACGT‐flank variants (GGGACGTTAT and CCCACGTGGG) validated by individual transient expression in tobacco without (−) and with (+) ABA treatment.

We further compared the activity of ACGT‐flank variants between control and ABA‐treated samples. Our analyses showed that most of these ACGT‐flank variants had modest effects on ABRE activity. However, there were 40 (1.7%) variants which significantly enhanced activity, while 54 (2.3%) variants which significantly blunted activity under ABA treatment (Figure 6A, Figure S2). The sequence logo showed a slight sequence preference for the enhancing variants. These ACGT‐flank variants with highly responsive activity were preferentially associated with a G directly following ACGT in the downstream region (Figure 6B,C). In contrast, we did not find any distinct pattern for the blunting variants under ABA treatment. To validate the high activity of ACGT‐flank variants with the preferred nucleotides, we compared the activity levels between the ABRE variants GGGACGTTAT and CCCACGTGGG by individual transient expression. We observed that changing the downstream sequence to the preferred motif resulted in a significant increase in ABA‐responsive activity, which was consistent with the findings from theSTARR‐seq experiment (Figure 6D). Taken together, these results suggested that the nucleotides following the ACGT motif play a crucial role in enhancing the responsive activity of ABRE element.

## 3 DISCUSSION

Deciphering the impact of sequence variation on *cis*‐regulatory function is crucial for understanding the precise control of gene expression in plants. Here, we present a synthetic STARR‐seq method for systematically evaluating the impact of sequence variations on *cis*‐regulatory function. In contrast to the original STARR‐seq method, a significant advantage of this synthetic version is that randomly synthesized DNA sequences enable a comprehensive and exhaustive mutational analysis of CREs [19]. Moreover, the synthetic STARR‐seq assay with transient transformation in tobacco is a highly accessible method, which would greatly expand the ability to directly measure the activity of CREs in response to environmental changes [20, 34].

Using the synthetic STARR‐seq method, we comprehensively evaluated the impact of sequence variations of ABRE elements for their activities. Our results showed that sequence changes in the ACGT‐core regions had a more pronounced effect on transcriptional activity than alternations of the ACGT‐flank sequences. It might be due to those bZIP transcription factors preferentially bound to DNA sequences that contained a conserved ACGT motif in plants [27, 35]. The synthetic STARR‐seq successfully identified several sequence features that determine ABRE activity. Specifically, a consensus sequence of G (G/T)G following the core motif contributes to enhancing the activity of ABRE elements, which matched well with the consensus derived from a small number of experimentally determined ABRE variants [26, 28]. In contrast to the ACGT‐flank variants, the core variants with low information content did not show any strong sequence preference for high activity.

The results from our STARR‐seq assays revealed that only a small fraction of ABRE variants, less than 10%, had significant changes in their transcriptional activity under ABA treatment, while most substitutions resulted in modest effects on the ABA‐responsive activity. Interestingly, our analysis showed that an alternative core with the AGAA motif could enhance the activity of ABRE variants in response to ABA. This finding suggested that non‐ACGT‐core elements might be involved in ABA‐regulated gene expression [36, 37]. Furthermore, we identified the preferred nucleotides following the ACGT‐core and experimentally validated the GGG motif, which could enhance the ABA‐responsive activity of ABRE elements.

Sequence variations within ABRE elements can influence their activity by altering the binding of bZIP transcription factors [26, 28, 29]. However, the binding specificity between ABRE elements and bZIP transcription factors remains unclear. The synthetic STARR‐seq assay carried out in the bZIP knockdown tobacco provides an opportunity to systematically discriminate the binding specificity of these bZIPs and evaluate their effects on ABRE activity. Moreover, the increasing STARR‐seq experiments have accelerated the development of computational methods for predicting the activity of CREs from DNA sequence [20, 38]. The data generated by this study will enable deep learning‐based models to accurately predict the activity of synthetic promoters in response to ABA, facilitating the design of condition‐dependent promoters in plants.

## 4 MATERIALS AND METHODS

### 4.1 STARR‐seq library design and generation

The STARR‐seq plasmids used here are based on the pGreenII 0800‐LUC vector [39]. The reporter constructs contain the ABRE variant, followed by the 35S minimal promoter (−46 to +5 relative to the TSS), the synthetic 5' UTR synJ (Kanoria and Burma, 2012; 5'‐ACACGCTGGAATTCTAGTATACTAAACC‐3'), an ATG start codon and a 12‐bp random barcode (5'‐VNNVNNVNNVNN‐3') in front of the second codon of the firefly luciferase gene. A 50‐bp regulatory sequence (5'‐TCTCCTTCCCATTTTCTTCGTCTACGTGTCGACCATCCACCGGTTTTTGT‐3') with an ABRE element was extracted from the promoter of the Arabidopsis *ABI1* gene [40]. To generate variant libraries, the oligonucleotides containing degenerate nucleotides (N) at ABRE sequences were synthesized by Shanghai Shenggong Bioengineering Co., Ltd. The synthesized oligo pool was then amplified using PCR to add the minimal promoter and the random barcodes with 12 cycles in 8 parallel reactions. The primer pairs and synthetic DNA templates used for constructing STARR‐seq libraries are shown in Table S1. After pooling the reactions, the amplified fragments were cloned into the pGreen II 0800‐LUC vector by one‐step cloning (Vazyme, Nanjing, China#C112‐02). A STARR‐seq library of ACGT‐core variants and four libraries of ACGT‐flank variants were generated, respectively. An additional library was constructed as a control, containing the 50‐bp enhancer (from −96 to −47 relative to the TSS) from the 35S promoter along with random barcodes. Approximately 100, 3000 and 40,000 colonies were respectively collected from the 35S enhancer, ACGT‐core, and ACGT‐flank libraries and then pooled for STARR‐seq assay.

### 4.2 Plant cultivation and transformation

Tobacco (*Nicotiana benthamiana*) seeds were sown in the soil with a 1:1 ratio of peat and vermiculite, and covered with a transparent plastic cover. After 1 week, the seedlings were transferred to an independent flowerpot, and agroinfiltration was carried out after 3–4 weeks. Tobacco plants were grown in the growth chamber at 25°C with a 16‐h light and an 8‐h dark photoperiod (light intensity 100 μmol photons m^−2^ s^−1^).

The pooled plasmid library was introduced into *Agrobacterium tumefaciens* strain GV3101 harboring the helper plasmid pSoup by electroporation. *Agrobacterium* carrying the plasmid library was grown overnight in the Luria–Bertani (LB) medium supplemented with kanamycin (50 μg/mL) and rifampicin (25 μg/mL) at 28°C to an OD_600_ of 1.0. The harvested cells were resuspended in the infiltration buffer (10 mmol/L MgCl_2_, 200 μmol/L acetosyringone) to OD_600_ of 0.8 and incubated for 3 h at room temperature. Agroinfiltration was carried out in the vacuum infiltration device (Shanghai Wonbio Biotechnology Co., Ltd). Upon starting the device, the vacuum chamber's pressure dropped to 80 mbar in about 30 s, allowing the suspension into the intracellular space between leaf epidermal cells [41]. The tobacco plants were further grown in the dark for 48 h before being brought back to the greenhouse. The infiltrated tobacco leaves were sprayed with 25 μmol/L ABA and harvested after 4‐h treatment.

### 4.3 ABRE‐barcode association sequencing and analysis

To link the barcode to the ABRE, PCR was performed on the plasmids isolated from the transformed *A. tumefaciens*. The ABRE‐barcode DNA fragments were amplified using PCR in 16 parallel reactions with 15 cycles (Table S2). After pooling the reactions, the PCR product was then sent for paired‐end sequencing on the Illumina NovaSeq 6000 platform (Shanghai Biozeron Biotechnology Co., Ltd). The overlapping paired‐end reads were joined into single sequences using fastq‐join. The ABRE‐barcode pairs perfectly matched to the designed sequences were extracted from the assembled reads. The read number was counted for each unique ABRE‐barcode pair and those with read counts below 10 were then excluded from the association analysis. A Python dictionary of ABRE‐barcode pairs was created to link unique barcodes to each ABRE variant. To improve the results of STARR‐seq, only ABRE variants associated with at least 5 unique barcodes were kept for further analyses.

### 4.4 RNA barcode sequencing and analysis

For each STARR‐seq experiment, three biological replicates were performed. Each sample of three to six leaves was collected from three tobacco plants. The samples were frozen in liquid nitrogen and ground into a fine powder with a mortar. Total RNA was extracted using the RNAprep pure Plant Kit (Tiangen, Beijing, China). First‐strand cDNA synthesis was performed using PrimeScript RT Reagent Kit (Takara, Beijing, China) following the manufacturer's protocol. Prior to reverse transcription, the RNA samples were treated twice with DNase I to eliminate the contaminating plasmid DNA. Eight reactions with 1‐μg RNA each and an LUC construct‐specific primer were prepared for cDNA synthesis. Half of the reactions were used as negative control, in which the reverse transcriptase was replaced with water. After cDNA synthesis, the reactions were pooled. To find the number of PCR cycles required for the following PCR reaction to amplify the barcode region, the real‐time quantitative PCR (qPCR) was performed for each sample. The barcode region was amplified using PCR with 24 cycles as determined by qPCR. The amplified DNA fragments were then collected from 16 parallel reactions. The primers are shown in Table S2. The barcode regions of six libraries, including three controls and three ABA‐treated samples, were read out by next‐generation sequencing on the Illumina NovaSeq 6000 platform (Shanghai Biozeron Biotechnology Co., Ltd).

Barcode sequences were extracted from the RNA sequencing dataset by Python scripts, and the read number was counted for each unique barcode. Barcodes with read counts of less than 5 were discarded in further analyses. The transcriptional strength was normalized with the input plasmid DNA for each promoter. The responsive activity of the ABRE variant was quantified as the fold change of transcriptional strength in ABA‐treated samples compared to controls. The ABA‐responsive variants were identified using DESeq2 with a *p* value < 0.05 and a fold change >1.2 [33]. A sequence probability logo to depict the consensus motif for ABRE variants was generated using kpLogo [42].

### 4.5 Validation of ABRE variants

To validate the activity of ABRE variants in our STARR‐seq, we selected two variants (GGGACGTTAT and CCCACGTGGG) and tested them individually for their function. The pGreenII 0800‐LUC vectors carrying the ABRE variant were introduced into tobacco leaves by *Agrobacterium‐*mediated transient transformation. The tobacco plants were treated with 25 μmol/L ABA after being kept in the dark for 48 h. After eliminating plasmid DNA with DNase I, the qRT‐PCR were conducted to determine the expression level of reporter genes. Three biological and three technical replicates were carried out for each sample. The activity of ABRE element was quantified by calculating the ratio of LUC to REN expression levels.

## AUTHOR CONTRIBUTIONS

**Fangnan Sun**: Conceptualization; investigation; formal analysis and writing‐original draft. **Yaxin Deng**: Conceptualization; data curation; investigation and writing‐original draft. **Weihua Zhao**: Conceptualization; investigation and writing‐original draft. **Yixue Xiong**: Formal analysis and visualization. **Lingxia Zhao**: Project administration and resources. **Lida Zhang**: Conceptualization; project administration; resources; supervision and writing‐review and editing.

## CONFLICT OF INTEREST STATEMENT

The authors declare that they have no competing financial interests or personal relationships that could have appeared to influence the work reported in this paper.

## ETHICS STATEMENT

This article does not contain any studies with human or animal materials performed by any of the authors.

## Supporting information

Supporting Information S1

## Data Availability

All sequencing reads were deposited in the National Center for Biotechnology Information (NCBI) Sequence Read Archive under the BioProject accession PRJNA1004068.
