# Supplementary material for: High‐throughput quantitative assessment of ABA‐responsive elements at single‐nucleotide resolution
Source: Quant Biol. 2025 Jan 30;13(2):e87. doi: 10.1002/qub2.87 (PMC12805990; doi:10.1002/qub2.87)
Supplement: Supplementary file 1 — Supporting Information S1 [file QUB2-13-e87-s001.pdf]

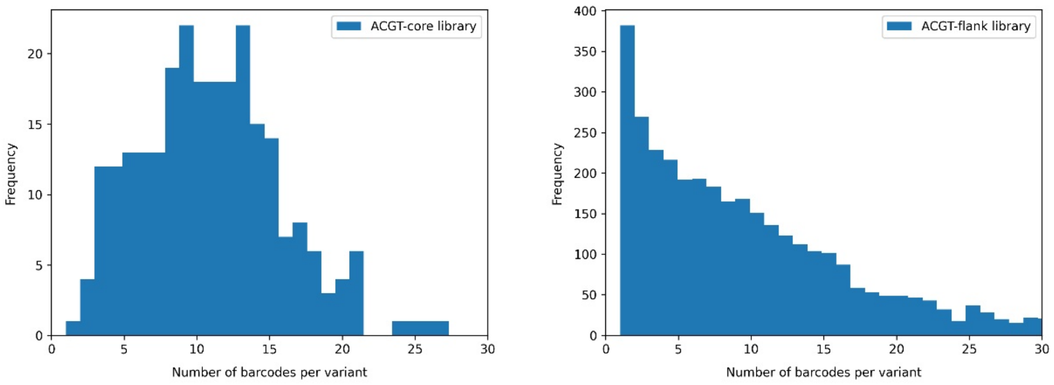

Figure S1. Distribution of observed unique barcode per ABRE.

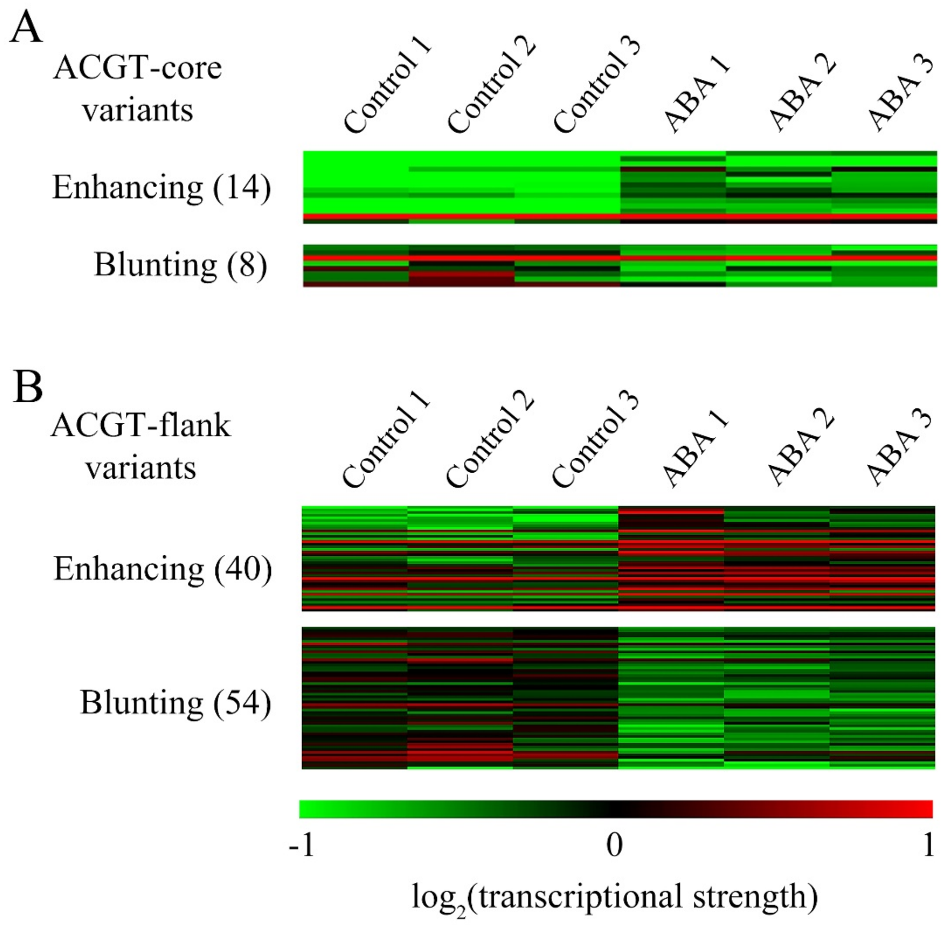

Figure S2. Heatmaps of enhancing and blunting ABRE variants. (A) ACGT-core variants. (A) ACGT-flank variants.

Table S1. Primers and DNA templates used for STARR-seq library construction.

Library	Primer/DNA template	Sequence (5'→3')
	ABRE-core-F	ACGACTCACTATAGGGCGAATTGGGTACCTCTCCTTCCCATTTTCTTCG
ABRE-core	ABRE-core-R	TCTTTATGTTTTTGGCAAGCTTGTCTTCNNBCNBGNBNNBCATGGTTTAGTATACTAGAATTCCAGCGTGTCGTGTCCTCTCCAAATGAAATGAACTTC
	ABRE-core-template	CCTCTCCTTCCCATTTTCTTCGTCTNNNNGTCGACCATCCACCGGTTTTTGTGCAAGACCCTTCCTCTATATAAGGAAGTTCATTTCATTTGGAGAGGA
	ABRE-flank1-F	ACGACTCACTATAGGGCGAATTGGGTACCTCTCCTTCCCATTTTCTTCG
ABRE-flank1	ABRE-flank1-R	TCTTTATGTTTTTGGCAAGCTTGTCTTCNNBGNBTNBNNBCATGGTTTAGTATACTAGAATTCCAGCGTGTCGTGTCCTCTCCAAATGAAATGAACTTC
	ABRE-flank1-template	CCTCTCCTTCCCATTTTCTTCGNNNACGTNNNGACCATCCACCGGTTTTTGTGCAAGACCCTTCCTCTATATAAGGAAGTTCATTTCATTTGGAGAGGA
	ABRE-flank2-F	ACGACTCACTATAGGGCGAATTGGGTACCTCTCCTTCCCATTTTCTTCG
ABRE-flank2	ABRE-flank2-R	TCTTTATGTTTTTGGCAAGCTTGTCTTCNNBTNBGNBNNBCATGGTTTAGTATACTAGAATTCCAGCGTGTCGTGTCCTCTCCAAATGAAATGAACTTC
	ABRE-flank2-template	CCTCTCCTTCCCATTTTCTTCGNNNACGTNNNGACCATCCACCGGTTTTTGTGCAAGACCCTTCCTCTATATAAGGAAGTTCATTTCATTTGGAGAGGA
	ABRE-flank3-F	ACGACTCACTATAGGGCGAATTGGGTACCTCTCCTTCCCATTTTCTTCG
ABRE-flank3	ABRE-flank3-R	TCTTTATGTTTTTGGCAAGCTTGTCTTCNNBCNBANBNNBCATGGTTTAGTATACTAGAATTCCAGCGTGTCGTGTCCTCTCCAAATGAAATGAACTTC
	ABRE-flank3-template	CCTCTCCTTCCCATTTTCTTCGNNNACGTNNNGACCATCCACCGGTTTTTGTGCAAGACCCTTCCTCTATATAAGGAAGTTCATTTCATTTGGAGAGGA
	ABRE-flank4-F	ACGACTCACTATAGGGCGAATTGGGTACCTCTCCTTCCCATTTTCTTCG
ABRE-flank4	ABRE-flank4-R	TCTTTATGTTTTTGGCAAGCTTGTCTTCNNBTNBCNBNNBCATGGTTTAGTATACTAGAATTCCAGCGTGTCGTGTCCTCTCCAAATGAAATGAACTTC
	ABRE-flank4-template	CCTCTCCTTCCCATTTTCTTCGNNNACGTNNNGACCATCCACCGGTTTTTGTGCAAGACCCTTCCTCTATATAAGGAAGTTCATTTCATTTGGAGAGGA
	35S-F	ACGACTCACTATAGGGCGAATTGGGTACCTGTGATATCTCCACTGACGTAAG
35S	35S-R	TCTTTATGTTTTTGGCAAGCTTGTCTTCNNBGNBGNBNNBCATGGTTTAGTATACTAGAATTCCAGCGTGTCGTGTCCTCTCCAAATGAAATGAACTTC
	35S-template	CCTGTGATATCTCCACTGACGTAAGGGATGACGCACAATCCCACTATCCTTCGCAAGACCCTTCCTCTATATAAGGAAGTTCATTTCATTTGGAGAGGA

Note: Overhangs used for one step cloning are indicated by underlining. Sequences in red represent the ABRE elements, sequences in orange represent the 5'UTR (SynJ) and sequences in blue represent barcodes.

Table S2. Primers used for DNA and RNA sequencing.

Primer	Sequence (5’→3’)	Note
ABRE-barcode-F	CGACGGCCAGTGAATTGTAATACG	DNA sequencing
ABRE-barcode-R	CATCTTCCAGCGGATAGAATGGC	
LUC-specific-R	GTTCATGATCAGTGCAATTGTCTTG	Reverse transcription
barcode-F	ACACGCTGGAATTCTAGTATACTAAACC	RNA sequencing
barcode-R	CAACCGAACGGACATTTCGAAGTAC	

Table S3. Promoter strength determined by STARR-seq.

Library	ABREs	Transcriptional strength (RNA/DNA)					
		Control 1	Control 2	Control 3	ABA 1	ABA 2	ABA 3
ACGT-core	TCTAAAGGTC	0.6334	0.8726	0.4429	0.7310	0.6401	0.4602
ACGT-core	TCTAACAGTC	1.0366	1.0579	0.8896	0.6144	1.0396	1.0878
ACGT-core	TCTAACGGTC	0.9317	0.6553	0.7614	1.0898	0.9132	0.8213
ACGT-core	TCTAAGAGTC	0.5867	0.6418	0.7169	0.4131	0.5930	0.6783
ACGT-core	TCTAAGGGTC	0.5312	0.8457	0.6497	0.5358	0.6094	0.6135
ACGT-core	TCTAAGTGTC	1.2823	1.6671	1.6979	1.5908	1.2459	1.4146
ACGT-core	TCTAATCGTC	0.5772	0.7394	0.6754	0.7168	0.6167	0.6339
ACGT-core	TCTAATGGTC	0.4135	0.4380	0.6785	0.4306	0.3698	0.7278
ACGT-core	TCTAATTGTC	0.5321	0.7689	0.3279	0.3966	0.4969	0.3571
ACGT-core	TCTACAAGTC	0.4120	0.8377	0.6329	0.8323	0.7250	0.4895
ACGT-core	TCTACACGTC	0.5535	0.5971	0.5559	0.7264	0.6732	0.6958
ACGT-core	TCTACAGGTC	0.7427	1.2514	0.6179	0.5366	0.4629	0.6386
ACGT-core	TCTACATGTC	0.5825	0.5754	0.6492	0.3735	0.7082	0.6026
ACGT-core	TCTACCAGTC	0.4598	0.5025	0.4163	0.1572	0.1506	0.4090
ACGT-core	TCTACCCGTC	1.2956	1.2680	1.0661	0.7432	1.3525	1.4036
ACGT-core	TCTACCGGTC	0.7598	0.8812	0.9122	0.6092	0.7154	0.8300
ACGT-core	TCTACCTGTC	0.4541	0.3853	0.7300	0.2391	0.3991	0.7148
ACGT-core	TCTACGAGTC	1.0915	1.2371	1.1940	0.9479	1.0190	1.0634
ACGT-core	TCTACGCGTC	0.9386	0.8015	1.4437	0.9425	0.7760	0.8464
ACGT-core	TCTACGGGTC	1.1739	0.9183	1.2920	0.7770	1.0660	1.0898
ACGT-core	TCTACGTGTC	0.8487	1.0783	1.2586	1.0290	0.9364	1.0209
ACGT-core	TCTACTCGTC	0.3231	0.5399	0.3788	0.4729	0.5314	0.4444
ACGT-core	TCTACTGGTC	0.6334	0.7584	0.8244	0.8586	0.7503	0.7462
ACGT-core	TCTAGAAGTC	0.1287	0.2393	0.2839	0.5339	0.5065	0.3516
ACGT-core	TCTAGACGTC	0.5069	0.8063	0.5674	0.7483	0.6991	0.6574
ACGT-core	TCTAGAGGTC	0.5661	0.6098	0.5330	0.4067	0.7354	0.6683
ACGT-core	TCTAGATGTC	0.5701	0.5477	0.3786	0.7495	0.8221	0.6366
ACGT-core	TCTAGCGGTC	0.8314	0.7550	1.1678	0.9350	0.8216	0.9298
ACGT-core	TCTAGCTGTC	0.3371	0.5974	0.5967	1.1868	0.6869	1.0729
ACGT-core	TCTAGGAGTC	0.5580	0.4787	0.5721	0.3165	0.6883	0.3826
ACGT-core	TCTAGGCGTC	0.7502	0.4900	0.5883	0.6716	0.6000	0.7977
ACGT-core	TCTAGGGGTC	0.6775	0.6878	0.8060	0.5171	0.6190	0.6122
ACGT-core	TCTAGGTGTC	0.4678	0.5438	0.3158	0.5123	0.5317	0.7145
ACGT-core	TCTAGTCGTC	0.7079	0.5809	0.4383	0.8306	0.6406	0.5889
ACGT-core	TCTAGTGGTC	0.4791	0.3919	0.2532	0.5171	0.4535	0.5433
ACGT-core	TCTAGTTGTC	0.5198	0.6011	0.7278	0.6600	0.4414	0.3686
ACGT-core	TCTATAAGTC	0.8146	0.9752	0.7357	0.9433	1.0829	1.0623
ACGT-core	TCTATACGTC	0.2624	0.1694	0.2920	0.4961	0.3988	0.3611
ACGT-core	TCTATAGGTC	1.9425	1.2002	1.5965	1.6853	2.1634	1.8359
ACGT-core	TCTATCAGTC	0.8343	0.4773	0.8183	0.7258	0.5186	0.7531
ACGT-core	TCTATCCGTC	1.0514	0.8568	0.6669	1.1365	1.1559	0.8563

ACGT-core	TCTATCGGTC	0.4479	0.2304	0.2323	0.2009	0.2955	0.2436
ACGT-core	TCTATCTGTC	0.3004	0.2007	0.3430	0.3037	0.4468	0.3909
ACGT-core	TCTATGAGTC	0.3633	0.2267	0.2010	0.4990	0.7088	0.7458
ACGT-core	TCTATGCGTC	0.8499	0.6442	0.8085	0.9886	1.0695	0.9604
ACGT-core	TCTATGGGTC	0.5806	0.8769	0.6951	0.4777	0.6630	0.5331
ACGT-core	TCTATGTGTC	0.7373	1.5241	0.7688	0.6797	0.6225	0.6891
ACGT-core	TCTATTAGTC	0.3981	0.6220	0.6374	0.9303	0.4851	0.6886
ACGT-core	TCTATTCGTC	0.8900	0.8826	1.1497	1.0538	0.9764	1.0291
ACGT-core	TCTATTGGTC	1.0957	0.5503	0.7454	0.6537	0.7913	0.9959
ACGT-core	TCTATTTGTC	0.4235	0.5133	0.5751	0.5463	0.6024	0.6361
ACGT-core	TCTCAATGTC	0.5585	0.5671	0.4287	0.6426	0.6602	0.5129
ACGT-core	TCTCACCGTC	1.2689	1.2237	1.3012	0.9402	0.6812	0.6571
ACGT-core	TCTCACGGTC	0.4048	0.3030	0.3455	0.6800	0.3483	0.6040
ACGT-core	TCTCACTGTC	0.4434	0.4480	0.3596	0.7046	0.8801	0.6129
ACGT-core	TCTCAGCGTC	0.6638	0.9877	0.7393	0.7726	0.7160	0.8441
ACGT-core	TCTCAGGGTC	0.9626	0.8775	0.9208	0.8143	0.9610	0.8757
ACGT-core	TCTCAGTGTC	0.9167	0.8525	0.6893	0.8625	0.9212	0.7715
ACGT-core	TCTCATGGTC	0.5407	0.5134	0.5203	0.6745	0.4935	0.5657
ACGT-core	TCTCATTGTC	0.6487	0.6200	0.8214	0.3671	0.5152	0.7396
ACGT-core	TCTCCAGGTC	0.4792	0.7820	0.5754	0.5503	0.6819	0.8019
ACGT-core	TCTCCATGTC	0.1957	0.5249	0.4363	0.5482	0.4515	0.3239
ACGT-core	TCTCCCAGTC	1.5242	1.3668	1.6547	1.3276	1.3574	1.3780
ACGT-core	TCTCCCGGTC	0.5340	0.5189	0.5988	0.7347	0.6538	0.6285
ACGT-core	TCTCCCTGTC	0.4635	0.3762	0.5769	0.3769	0.6663	0.5772
ACGT-core	TCTCCGAGTC	0.7848	0.4238	1.4904	0.9066	1.0351	1.0002
ACGT-core	TCTCCGCGTC	0.4740	0.6028	0.8779	0.6861	0.6805	0.7209
ACGT-core	TCTCCGGGTC	0.9199	0.9018	0.7776	0.9542	0.7885	0.9816
ACGT-core	TCTCCGTGTC	0.8082	0.9857	0.6735	0.7129	0.9325	0.8553
ACGT-core	TCTCCTAGTC	0.9521	0.7231	0.7260	0.7501	0.7327	0.8730
ACGT-core	TCTCCTCGTC	0.9707	0.6929	0.6049	0.3475	0.8015	0.4518
ACGT-core	TCTCCTGGTC	0.8754	0.7708	0.7863	0.6601	0.7088	0.9285
ACGT-core	TCTCCTTGTC	0.6994	0.4159	0.5800	0.5697	0.6720	0.6112
ACGT-core	TCTCGAAGTC	0.7192	0.6629	1.8513	1.2847	0.9685	0.9475
ACGT-core	TCTCGACGTC	0.9879	0.5754	0.9394	0.9822	0.9240	0.9282
ACGT-core	TCTCGAGGTC	0.7256	0.5250	0.4877	0.7633	0.6303	0.6103
ACGT-core	TCTCGATGTC	0.9169	1.0728	1.6314	1.1207	0.9355	0.7856
ACGT-core	TCTCGCAGTC	0.4397	0.4170	0.2421	0.5106	0.4013	0.5434
ACGT-core	TCTCGCCGTC	3.4594	2.3552	2.9725	4.0453	2.7694	3.0562
ACGT-core	TCTCGCGGTC	0.7285	0.7327	0.5724	0.8683	0.6437	0.5034
ACGT-core	TCTCGCTGTC	0.5271	0.7511	0.5766	0.3486	0.6452	0.6541
ACGT-core	TCTCGGAGTC	0.4518	0.6775	1.0981	0.6938	0.6948	0.5317
ACGT-core	TCTCGGCGTC	0.7425	0.6274	0.5800	0.7909	0.5976	0.6300
ACGT-core	TCTCGGGGTC	0.8030	0.7818	0.7818	0.7246	0.7512	0.7592
ACGT-core	TCTCGGTGTC	0.6896	0.7606	0.5224	0.6868	0.8428	0.7006

ACGT-core	TCTCGTAGTC	0.6020	0.7822	0.8147	0.6291	0.8927	0.7380
ACGT-core	TCTCGTCGTC	0.4783	0.4225	0.3558	0.4910	0.5723	0.5482
ACGT-core	TCTCGTGGTC	0.9842	0.8031	0.7102	0.7254	0.7727	0.8607
ACGT-core	TCTCGTTGTC	0.3461	0.4675	0.1771	0.4453	0.3475	0.2921
ACGT-core	TCTCTACGTC	0.6143	0.5552	0.5149	0.4717	0.8045	0.6077
ACGT-core	TCTCTAGGTC	0.5284	0.7973	0.6892	0.5805	0.6410	0.7084
ACGT-core	TCTCTCCGTC	0.4941	0.7941	0.6188	0.6556	0.6880	0.6112
ACGT-core	TCTCTCGGTC	0.6861	0.5749	0.7569	0.6811	0.6739	0.6343
ACGT-core	TCTCTCTGTC	0.6257	0.6534	0.5543	0.8214	0.9282	0.9026
ACGT-core	TCTCTGAGTC	0.8614	0.7117	0.3526	0.8127	0.7755	0.8785
ACGT-core	TCTCTGCGTC	0.5744	0.6020	0.7013	0.4603	0.5268	0.7512
ACGT-core	TCTCTGGGTC	0.5869	0.4266	0.6998	0.5846	0.5447	0.5642
ACGT-core	TCTCTGTGTC	0.4980	0.7986	0.4937	0.6811	0.7605	0.5799
ACGT-core	TCTCTTAGTC	0.7423	0.9256	1.1947	1.0843	1.0175	0.8347
ACGT-core	TCTCTTCGTC	1.1586	0.7113	1.4594	0.8168	0.9914	0.9280
ACGT-core	TCTCTTGGTC	0.7616	0.3002	0.7626	0.5025	0.5561	0.6615
ACGT-core	TCTCTTTGTC	5.6165	5.5985	7.2858	6.7447	6.4621	5.7493
ACGT-core	TCTGAAAGTC	0.4785	0.5868	0.6552	0.3646	0.4363	0.4911
ACGT-core	TCTGAACGTC	0.8194	0.7218	1.0621	0.7615	0.8847	0.8444
ACGT-core	TCTGAAGGTC	1.0233	0.5588	0.6686	0.4145	0.5408	0.6904
ACGT-core	TCTGAATGTC	0.6314	0.4891	0.3221	0.3827	0.4724	0.2790
ACGT-core	TCTGACAGTC	0.3848	0.2644	0.7100	0.3588	0.5936	0.6357
ACGT-core	TCTGACCGTC	2.8450	3.4449	2.5101	1.7460	2.3075	2.4878
ACGT-core	TCTGACGGTC	0.5167	0.4792	0.5994	0.6323	0.6081	0.6138
ACGT-core	TCTGACTGTC	0.8302	1.0127	0.9645	0.5971	0.9601	1.1033
ACGT-core	TCTGAGAGTC	1.0413	0.8342	1.1788	1.1507	0.9695	1.2048
ACGT-core	TCTGAGCGTC	0.9785	0.3091	0.7705	0.5109	0.5295	0.7053
ACGT-core	TCTGAGGGTC	0.8181	0.7526	0.8339	0.8590	0.8134	0.6735
ACGT-core	TCTGAGTGTC	0.5266	0.6866	0.4629	0.5798	0.3863	0.5498
ACGT-core	TCTGATAGTC	0.5718	0.9488	0.6858	0.5907	0.5175	0.4929
ACGT-core	TCTGATCGTC	0.5177	0.6700	0.7010	0.6662	0.6203	0.4578
ACGT-core	TCTGATGGTC	0.7665	0.8961	0.6544	0.5551	0.6324	0.6230
ACGT-core	TCTGATTGTC	0.9998	0.5477	1.0474	0.2964	0.6767	0.6902
ACGT-core	TCTGCAAGTC	0.7602	0.7109	0.3554	0.5201	0.5029	0.4250
ACGT-core	TCTGCACGTC	0.8334	1.2130	0.8769	1.0013	0.7814	1.0092
ACGT-core	TCTGCAGGTC	0.8853	0.9879	1.0020	0.9526	0.7315	0.8782
ACGT-core	TCTGCATGTC	0.4784	0.5583	0.4077	0.5313	0.3737	0.4074
ACGT-core	TCTGCCAGTC	1.1201	0.9444	0.9846	1.0193	0.6952	0.9552
ACGT-core	TCTGCCCGTC	7.9368	8.3540	8.5909	6.8016	7.8287	9.4241
ACGT-core	TCTGCCGGTC	0.6013	0.8174	0.7964	0.7405	0.5181	0.8518
ACGT-core	TCTGCCTGTC	0.6797	0.7805	0.4850	0.7004	0.7300	0.8473
ACGT-core	TCTGCGAGTC	0.9921	0.9642	0.6829	0.5595	0.7260	0.6856
ACGT-core	TCTGCGCGTC	0.5026	0.4969	0.7806	0.6944	0.6754	0.7583
ACGT-core	TCTGCGGGTC	0.7532	0.7697	0.6859	0.6164	0.6657	0.5440

ACGT-core	TCTGCGTGTC	0.5910	0.6487	0.4548	0.4281	0.4396	0.4960
ACGT-core	TCTGCTAGTC	0.7370	0.8257	0.6522	0.6480	0.7825	0.7614
ACGT-core	TCTGCTCGTC	0.7441	0.3484	0.6713	0.7314	0.4718	0.5216
ACGT-core	TCTGCTGGTC	0.4821	0.5426	0.5901	0.2611	0.4121	0.5204
ACGT-core	TCTGCTTGTC	0.4163	0.5380	0.3744	0.6519	0.6290	0.4298
ACGT-core	TCTGGAAGTC	0.5380	0.7096	0.9068	0.7938	0.8374	0.7150
ACGT-core	TCTGGACGTC	1.2489	1.4579	1.3531	1.1806	0.8842	1.3551
ACGT-core	TCTGGAGGTC	0.7339	0.5833	0.7154	0.5017	0.6421	0.7135
ACGT-core	TCTGGATGTC	0.3971	0.4953	0.4528	0.6997	0.6137	0.5525
ACGT-core	TCTGGCAGTC	0.4904	0.4461	0.9100	0.6010	0.4408	0.7006
ACGT-core	TCTGGCGGTC	0.7979	0.8976	0.8175	0.8834	0.8491	0.8319
ACGT-core	TCTGGCTGTC	0.4248	0.5083	0.4490	0.5697	0.5743	0.5162
ACGT-core	TCTGGGAGTC	0.3292	0.5023	0.4915	0.5223	0.4430	0.3271
ACGT-core	TCTGGGCGTC	0.8755	1.0970	0.4980	1.0143	0.6463	0.9842
ACGT-core	TCTGGGGGTC	0.6987	0.6076	0.8579	0.7516	0.7117	0.7338
ACGT-core	TCTGGGTGTC	0.6212	0.4100	0.4840	0.4193	0.5575	0.6673
ACGT-core	TCTGGTAGTC	0.5710	0.8962	0.4675	0.4447	0.4795	0.4457
ACGT-core	TCTGGTCGTC	0.5816	0.7490	0.6035	0.6694	0.6356	0.5913
ACGT-core	TCTGGTGGTC	0.5314	0.6888	0.5503	0.5307	0.6674	0.5540
ACGT-core	TCTGGTTGTC	0.6064	0.6668	0.7519	0.6924	0.5786	0.7362
ACGT-core	TCTGTAAGTC	0.6068	1.1719	0.4746	0.5191	0.6731	0.5807
ACGT-core	TCTGTACGTC	0.4605	0.4458	0.4922	0.6300	0.6633	0.6944
ACGT-core	TCTGTAGGTC	0.4180	0.7200	0.4054	0.4271	0.6007	0.6096
ACGT-core	TCTGTATGTC	0.4509	0.9029	0.7509	1.0022	0.7900	0.6077
ACGT-core	TCTGTCAGTC	0.7022	1.0073	0.6278	0.7432	0.6090	0.5936
ACGT-core	TCTGTCCGTC	3.5374	3.8126	3.3504	4.5771	5.3961	4.3393
ACGT-core	TCTGTCGGTC	0.4899	0.4626	0.3512	0.5860	0.5857	0.6325
ACGT-core	TCTGTCTGTC	0.5190	0.6106	0.6228	0.7429	0.6186	0.5735
ACGT-core	TCTGTGAGTC	0.5291	1.0245	0.7535	0.7038	0.6935	0.7419
ACGT-core	TCTGTGCGTC	0.6276	0.7061	0.6839	0.6661	0.6124	0.5650
ACGT-core	TCTGTGGGTC	0.6649	0.5690	0.8377	0.4253	0.6574	0.7135
ACGT-core	TCTGTGTGTC	0.7209	0.7951	0.7570	0.6522	0.6063	0.5002
ACGT-core	TCTGTTAGTC	0.5687	0.7636	0.9647	0.5305	0.7024	0.7326
ACGT-core	TCTGTTCGTC	0.6353	0.7027	0.8345	0.8113	0.6234	0.6084
ACGT-core	TCTGTTGGTC	0.9360	0.7686	0.9181	0.8763	0.7806	0.8548
ACGT-core	TCTGTTTGTC	0.9266	0.9653	1.0506	0.8299	1.1330	1.0144
ACGT-core	TCTTAAAGTC	0.4776	0.6440	0.3474	0.4080	0.7203	0.7278
ACGT-core	TCTTAAGGTC	0.6221	0.3608	0.9852	0.5943	0.5860	0.7130
ACGT-core	TCTTACCGTC	0.6055	0.5803	0.4341	0.7024	0.5877	0.4819
ACGT-core	TCTTACGGTC	0.4585	0.6607	0.3696	0.4421	0.2814	0.5727
ACGT-core	TCTTACTGTC	0.8153	1.1362	0.5705	0.6915	0.7958	0.6934
ACGT-core	TCTTAGAGTC	1.2317	0.8299	0.9408	0.5489	0.8879	0.7176
ACGT-core	TCTTAGGGTC	0.6736	0.9033	0.7515	0.7552	0.7687	0.9169
ACGT-core	TCTTATAGTC	0.6781	1.1778	0.9798	1.0774	1.1494	1.2578

ACGT-core	TCTTATCGTC	0.4455	0.5879	0.2525	0.6473	0.4818	0.4042
ACGT-core	TCTTATGGTC	0.8317	1.2522	0.8829	0.7032	0.8649	0.8351
ACGT-core	TCTTATTGTC	0.5431	0.2809	0.8369	0.7262	0.8059	0.7688
ACGT-core	TCTTCAAGTC	0.9768	0.8281	1.1126	0.9996	0.8145	1.0692
ACGT-core	TCTTCACGTC	0.6519	0.4959	0.4598	0.5793	0.6181	0.7107
ACGT-core	TCTTCAGGTC	0.8470	0.8663	0.6826	0.8126	0.8742	0.9208
ACGT-core	TCTTCATGTC	0.5002	0.4603	0.8320	0.7638	0.6486	0.6305
ACGT-core	TCTTCCAGTC	1.0574	0.7755	1.4381	0.9244	0.9283	1.0546
ACGT-core	TCTTCCCGTC	0.4137	0.8612	0.5305	0.5663	0.4514	0.8031
ACGT-core	TCTTCCGGTC	0.9592	0.6385	0.8428	0.4541	0.8651	0.8226
ACGT-core	TCTTCCTGTC	0.4307	0.4562	0.5105	0.8126	0.6496	0.6208
ACGT-core	TCTTCGAGTC	0.6071	0.2255	0.6441	0.3867	0.7648	0.7093
ACGT-core	TCTTCGCGTC	0.3861	0.3577	0.5107	0.7276	0.3979	0.4600
ACGT-core	TCTTCGGGTC	0.4100	0.7067	0.4582	0.6082	0.5957	0.4957
ACGT-core	TCTTCGTGTC	0.9768	0.7028	0.5831	0.8233	0.6265	0.6081
ACGT-core	TCTTCTAGTC	0.2372	0.3369	0.1182	0.7357	0.4867	0.4261
ACGT-core	TCTTCTCGTC	0.4984	0.2487	0.2994	0.4682	0.3598	0.4575
ACGT-core	TCTTCTGGTC	0.8039	0.6502	0.8786	0.7420	0.6448	0.8823
ACGT-core	TCTTCTTGTC	1.1797	1.0220	0.9056	0.8571	1.0606	0.9395
ACGT-core	TCTTGAAGTC	0.7253	0.6575	0.5881	0.6932	0.7904	0.6693
ACGT-core	TCTTGACGTC	0.6957	0.9098	1.1804	0.7061	0.9827	0.8846
ACGT-core	TCTTGAGGTC	0.7114	0.7370	0.6664	0.6750	0.8241	0.7357
ACGT-core	TCTTGATGTC	1.7444	0.8149	1.2190	1.3125	1.3862	1.1075
ACGT-core	TCTTGCCGTC	3.2081	3.1734	3.3715	3.8491	3.0262	2.3336
ACGT-core	TCTTGCGGTC	0.8579	0.7138	0.6042	0.6977	0.8543	0.7439
ACGT-core	TCTTGCTGTC	0.5295	0.8908	0.4522	0.4785	0.4712	0.5049
ACGT-core	TCTTGGAGTC	0.5382	0.7499	0.7391	0.6595	0.7894	0.6217
ACGT-core	TCTTGGCGTC	0.5162	0.5678	0.5288	0.5992	0.6144	0.6364
ACGT-core	TCTTGGGGTC	0.7150	0.9778	0.9173	0.7043	0.8776	0.7518
ACGT-core	TCTTGGTGTC	0.9170	0.5733	0.9161	0.7862	0.6008	0.6878
ACGT-core	TCTTGTAGTC	0.5797	0.6774	0.5708	0.5850	1.0276	0.6423
ACGT-core	TCTTGTCGTC	1.0151	1.5344	0.7545	1.2161	0.8947	1.0517
ACGT-core	TCTTGTGGTC	0.8255	0.8738	0.9161	0.7315	0.8584	0.7963
ACGT-core	TCTTGTTGTC	0.7997	0.6357	0.6790	0.8252	0.7876	0.6160
ACGT-core	TCTTTACGTC	0.2401	0.1878	0.4129	0.5131	0.3195	0.4513
ACGT-core	TCTTTAGGTC	1.2685	0.9368	1.0568	1.1253	0.9564	0.9695
ACGT-core	TCTTTATGTC	0.6181	0.5393	0.9182	0.7049	0.7957	0.6459
ACGT-core	TCTTTCAGTC	0.5900	0.3400	0.4085	0.1718	0.4549	0.3620
ACGT-core	TCTTTCCGTC	0.4034	0.6411	0.3257	0.5507	0.5418	0.6018
ACGT-core	TCTTTCGGTC	0.8003	0.9146	0.9197	0.6024	0.5910	0.8235
ACGT-core	TCTTTGAGTC	0.8333	0.6875	0.9610	0.6832	0.4249	0.8573
ACGT-core	TCTTTGCGTC	0.1784	0.5011	0.9188	0.5145	0.5275	0.5683
ACGT-core	TCTTTGGGTC	0.7791	0.6808	0.4153	0.3994	0.5647	0.6451
ACGT-core	TCTTTGTGTC	0.8188	1.0822	0.7699	0.8632	0.7138	0.7667

ACGT-core	TCTTTTCGTC	0.7670	0.5762	0.8265	0.5945	0.5211	0.6062
ACGT-core	TCTTTTGGTC	1.1579	0.7667	1.2263	1.0164	1.0876	1.1453
ACGT-core	TCTTTTTGTC	0.7252	0.5819	0.5822	0.8967	0.7534	0.6458
ACGT-flank	AAAACGTAAA	0.8658	0.8004	0.7832	0.6672	0.8698	0.8392
ACGT-flank	AAAACGTAAG	0.9816	1.0823	1.0027	0.9038	0.9217	0.9034
ACGT-flank	AAAACGTACG	0.6245	0.6591	0.5757	0.5575	0.5309	0.6390
ACGT-flank	AAAACGTAGA	0.7252	0.8009	0.9045	0.7873	0.7859	0.7916
ACGT-flank	AAAACGTAGC	0.8207	1.1116	0.8040	0.7903	0.8286	0.8345
ACGT-flank	AAAACGTAGG	0.7977	0.8841	0.7725	0.7782	0.8121	0.8200
ACGT-flank	AAAACGTAGT	0.4784	0.7190	0.5597	0.6434	0.7992	0.7887
ACGT-flank	AAAACGTCAA	0.7751	0.9544	0.8029	0.6867	0.6533	0.7285
ACGT-flank	AAAACGTCAG	1.2917	0.8470	0.9556	1.0260	0.8997	1.0145
ACGT-flank	AAAACGTCCA	0.9494	0.9269	1.0286	0.7158	0.7818	0.6815
ACGT-flank	AAAACGTCCC	0.5264	0.6046	0.6059	0.5034	0.4961	0.5458
ACGT-flank	AAAACGTCCG	0.8878	0.8210	0.9187	1.0187	1.0179	0.9881
ACGT-flank	AAAACGTCCT	0.9197	0.8932	1.0661	1.0059	0.8745	0.9635
ACGT-flank	AAAACGTCGA	1.0712	0.8676	0.8235	0.8221	0.6597	0.7541
ACGT-flank	AAAACGTCGC	0.6212	0.5944	0.5568	0.5214	0.7474	0.6511
ACGT-flank	AAAACGTCGG	0.9613	0.8861	1.0104	0.7505	0.7922	0.8192
ACGT-flank	AAAACGTCGT	0.5879	0.5969	0.5813	0.6845	0.6608	0.6173
ACGT-flank	AAAACGTCTG	0.7391	0.8911	0.6926	0.7847	0.8442	0.7534
ACGT-flank	AAAACGTGAA	0.9173	0.9854	0.8984	0.8354	1.0067	0.9297
ACGT-flank	AAAACGTGAC	0.6468	0.8532	0.6868	0.6474	0.7077	0.7104
ACGT-flank	AAAACGTGAG	0.7890	0.8330	0.9817	0.9124	0.7755	0.8050
ACGT-flank	AAAACGTGAT	0.9849	0.8872	1.0267	0.8785	0.8791	1.0374
ACGT-flank	AAAACGTGCA	0.8220	0.9104	0.8116	0.8006	0.9845	0.9919
ACGT-flank	AAAACGTGCG	0.7782	0.6109	0.4847	0.5308	0.6443	0.6612
ACGT-flank	AAAACGTGGA	0.8477	0.8606	0.8684	0.8533	0.8616	0.9577
ACGT-flank	AAAACGTGGC	0.8878	0.9529	0.8295	0.9292	0.8374	0.8801
ACGT-flank	AAAACGTGGG	1.0724	1.4285	1.2295	1.2182	1.2217	1.2174
ACGT-flank	AAAACGTGGT	0.8363	0.8304	0.7863	0.7152	0.6865	0.7156
ACGT-flank	AAAACGTGTA	0.8283	0.4256	0.6324	0.9514	0.8750	0.8047
ACGT-flank	AAAACGTGTC	1.1085	1.0697	0.9590	0.9776	1.0490	0.9198
ACGT-flank	AAAACGTGTG	2.1861	2.0037	2.0457	2.2180	2.0663	2.0473
ACGT-flank	AAAACGTTCG	1.0398	1.5368	1.0250	1.1625	1.2841	1.0717
ACGT-flank	AAAACGTTGA	0.8631	0.9181	0.6720	0.7476	0.7366	0.6677
ACGT-flank	AAAACGTTGC	1.3538	0.6554	0.6477	0.5356	0.5920	0.7218
ACGT-flank	AAAACGTTGG	0.7436	0.7702	0.9344	0.9127	0.7935	0.8240
ACGT-flank	AACACGTAAG	0.9920	0.7344	1.2498	0.8177	1.1102	0.7704
ACGT-flank	AACACGTACC	0.9624	0.9727	0.8758	0.7595	0.9002	0.8464
ACGT-flank	AACACGTACG	0.7594	0.8614	0.8573	0.9074	0.8709	0.8065
ACGT-flank	AACACGTAGA	0.9687	0.7765	0.7040	0.9203	0.9074	0.9509
ACGT-flank	AACACGTAGG	0.9892	0.9086	0.8914	0.9499	1.0375	0.9859
ACGT-flank	AACACGTAGT	0.9197	0.9416	0.8212	0.7854	1.0428	0.9598

ACGT-flank	AACACGTATG	0.6066	0.6916	0.7721	0.6238	0.9543	0.4155
ACGT-flank	AACACGTCAG	0.8635	0.9093	0.9299	0.8667	0.8792	0.8411
ACGT-flank	AACACGTCCG	1.5545	1.2008	1.7549	0.9174	1.3239	1.3467
ACGT-flank	AACACGTCGA	1.0425	1.1514	1.0336	0.9692	1.1569	0.9705
ACGT-flank	AACACGTCGC	0.6518	0.9195	0.6685	0.7678	0.5969	0.7340
ACGT-flank	AACACGTCGG	0.7724	1.0103	0.9289	0.8553	0.9394	0.9319
ACGT-flank	AACACGTCGT	0.8387	0.8303	0.9642	0.6417	0.7827	0.7862
ACGT-flank	AACACGTCTG	0.9359	0.8253	0.7350	0.7448	0.6397	0.7253
ACGT-flank	AACACGTGAA	0.6371	1.0323	0.9048	0.8077	0.9931	0.8083
ACGT-flank	AACACGTGAG	0.9963	0.8517	0.5319	0.9572	0.7565	0.6393
ACGT-flank	AACACGTGCA	1.4239	1.4643	1.2918	1.6397	1.4914	1.2654
ACGT-flank	AACACGTGCG	1.1467	0.9560	0.9749	0.7962	0.8116	0.8069
ACGT-flank	AACACGTGCT	1.1469	1.1838	0.9557	1.1187	0.9442	0.7319
ACGT-flank	AACACGTGGA	1.2076	1.1029	1.1342	1.3422	1.1511	1.0653
ACGT-flank	AACACGTGGC	1.6294	1.7427	1.9079	2.2194	2.1459	1.9406
ACGT-flank	AACACGTGGG	1.6217	1.6131	1.5026	1.6030	1.4234	1.3690
ACGT-flank	AACACGTGGT	1.0312	0.9503	1.0946	1.2645	1.3098	0.9766
ACGT-flank	AACACGTGTC	1.6550	1.7739	1.7047	2.1536	2.0002	1.7010
ACGT-flank	AACACGTGTG	1.0331	1.1005	0.9759	1.0734	1.0628	1.1865
ACGT-flank	AACACGTTAG	0.9530	0.7852	0.8892	0.6486	0.7289	0.8313
ACGT-flank	AACACGTTGA	0.8028	1.4265	0.7675	1.0167	1.2223	0.8013
ACGT-flank	AACACGTTGG	0.6809	0.7139	0.7171	0.6935	0.6696	0.6693
ACGT-flank	AAGACGTAAA	0.8371	0.8095	0.8251	0.8676	0.8485	0.7837
ACGT-flank	AAGACGTAAC	0.9376	0.9733	0.7894	0.8626	0.8890	0.8454
ACGT-flank	AAGACGTAAG	1.0433	0.9144	1.1129	0.9829	1.0502	1.0589
ACGT-flank	AAGACGTAAT	0.6624	0.9886	0.4738	0.8074	0.7538	0.8271
ACGT-flank	AAGACGTACA	0.8140	0.9727	0.8429	0.9043	0.8606	0.8514
ACGT-flank	AAGACGTACG	1.1495	0.8467	0.8915	0.9610	1.0615	0.9333
ACGT-flank	AAGACGTACT	0.8585	0.4156	1.0619	0.8757	0.9770	0.7080
ACGT-flank	AAGACGTAGA	0.8355	0.6391	0.8343	0.9666	0.8952	0.7962
ACGT-flank	AAGACGTAGC	1.2088	1.0860	1.4775	1.0872	1.2148	1.4048
ACGT-flank	AAGACGTAGG	0.6132	0.5953	0.6803	0.6970	0.5567	0.5223
ACGT-flank	AAGACGTAGT	0.6194	0.9545	0.7933	0.9760	0.7980	0.7714
ACGT-flank	AAGACGTATG	1.0060	0.8483	0.8294	0.9613	0.8780	0.8534
ACGT-flank	AAGACGTCAA	0.8657	1.0798	0.7156	1.0125	0.8283	0.7618
ACGT-flank	AAGACGTCAG	1.0249	0.8683	0.9248	0.9900	0.9249	0.7459
ACGT-flank	AAGACGTCCA	0.9300	0.8260	0.7698	0.8141	0.8818	0.6944
ACGT-flank	AAGACGTCCG	0.8362	0.8689	0.7661	0.8468	0.5852	0.9851
ACGT-flank	AAGACGTCCT	0.7647	0.6891	0.7902	1.3579	0.8642	0.7649
ACGT-flank	AAGACGTCGA	1.0859	0.6256	0.9697	0.9942	0.9431	1.1495
ACGT-flank	AAGACGTCGC	1.0391	1.3614	1.2470	1.4928	0.8036	1.3911
ACGT-flank	AAGACGTCGG	0.7458	0.6884	0.8207	0.8168	0.7739	0.8761
ACGT-flank	AAGACGTCGT	1.0908	1.4071	1.4576	1.0130	1.1572	1.2607
ACGT-flank	AAGACGTCTA	0.8341	0.9239	0.8301	0.7842	0.7776	0.9119

ACGT-flank	AAGACGTCTC	0.9574	0.6428	0.8438	0.8797	0.7143	0.8941
ACGT-flank	AAGACGTCTG	1.1372	1.3105	0.9448	1.0490	0.9482	1.0262
ACGT-flank	AAGACGTGAA	1.0601	0.7499	1.1543	1.1426	1.0439	1.0726
ACGT-flank	AAGACGTGAC	0.8588	1.1753	1.0553	0.9469	1.0074	0.9004
ACGT-flank	AAGACGTGAG	0.9442	1.2161	0.9155	0.9975	0.9543	0.8586
ACGT-flank	AAGACGTGAT	0.7660	0.9497	0.5430	0.9596	0.7383	0.7285
ACGT-flank	AAGACGTGCG	0.8879	0.6312	0.8064	0.6453	0.9740	1.0622
ACGT-flank	AAGACGTGCT	0.9335	0.7286	1.0661	0.8659	0.9857	0.7699
ACGT-flank	AAGACGTGGA	1.0308	0.8710	0.7834	0.8484	0.8820	0.8801
ACGT-flank	AAGACGTGGC	0.6202	0.9052	0.9689	0.9705	0.8679	1.1190
ACGT-flank	AAGACGTGGG	1.3217	1.4208	1.2094	1.1874	1.2024	1.1601
ACGT-flank	AAGACGTGGT	0.9045	0.7885	0.9280	0.6991	0.7278	0.7606
ACGT-flank	AAGACGTGTA	0.9367	0.8621	0.9979	0.8115	0.7725	1.0251
ACGT-flank	AAGACGTGTC	0.9779	0.9555	1.1481	1.1893	1.0079	1.0672
ACGT-flank	AAGACGTGTG	1.3425	1.3622	1.2209	1.4055	1.3864	1.2885
ACGT-flank	AAGACGTTAC	0.7591	0.3561	0.4073	1.0079	0.5750	0.6648
ACGT-flank	AAGACGTTAG	0.8872	0.8714	0.8390	0.9303	0.8700	0.9079
ACGT-flank	AAGACGTTCA	1.0187	0.9614	1.3238	1.0107	1.0234	1.0885
ACGT-flank	AAGACGTTCG	0.9639	0.8038	0.9076	0.7618	0.9081	0.8034
ACGT-flank	AAGACGTTGA	0.8251	0.8884	0.7554	0.6164	0.7089	0.8336
ACGT-flank	AAGACGTTGC	0.8576	0.8032	0.9434	0.7956	0.7741	0.7787
ACGT-flank	AAGACGTTGG	1.0464	0.9672	0.9316	0.8578	1.0619	0.9452
ACGT-flank	AAGACGTTGT	1.1895	0.8570	1.2798	0.8668	1.3123	1.0571
ACGT-flank	AAGACGTTTG	0.8299	0.6627	1.0439	0.8795	0.9167	0.8152
ACGT-flank	AATACGTAGC	0.8905	0.9821	0.6740	0.8651	0.9148	0.5446
ACGT-flank	AATACGTAGG	1.0614	1.0127	1.2949	1.1354	1.4420	1.2662
ACGT-flank	AATACGTCCA	0.8765	0.6656	0.4331	0.2501	0.6533	0.4099
ACGT-flank	AATACGTCCG	1.0147	1.0562	0.9440	0.9955	1.0948	1.0014
ACGT-flank	AATACGTCGA	1.0184	1.3788	1.0271	1.0430	1.0671	1.0538
ACGT-flank	AATACGTCGG	1.0398	1.0608	0.7814	1.0315	0.9315	0.8251
ACGT-flank	AATACGTGAG	0.6296	0.6013	0.5469	0.5700	0.8389	0.8034
ACGT-flank	AATACGTGCA	0.4919	0.6851	0.5769	0.7698	0.6094	0.6286
ACGT-flank	AATACGTGCC	0.7537	0.7649	0.6805	0.6496	0.7067	0.7515
ACGT-flank	AATACGTGCG	0.9651	1.0285	0.9616	0.9639	0.8873	0.8493
ACGT-flank	AATACGTGGA	1.0636	0.9514	0.8759	0.8516	0.7722	0.8896
ACGT-flank	AATACGTGGC	1.3117	1.3385	1.2207	1.3573	1.2426	1.4149
ACGT-flank	AATACGTGGG	1.2521	1.0371	1.2762	1.2768	1.1562	1.1872
ACGT-flank	AATACGTGTC	1.1736	0.9176	0.9550	1.3142	1.1843	0.9929
ACGT-flank	AATACGTGTG	1.3897	1.7881	1.2889	1.9706	1.5095	1.4548
ACGT-flank	ACAACGTAAA	0.6650	1.3191	0.7424	0.7954	0.7940	0.8614
ACGT-flank	ACAACGTAAG	0.9653	0.7682	1.0675	0.7706	0.8078	0.9186
ACGT-flank	ACAACGTACA	0.6316	0.7125	0.7052	0.8441	0.8405	0.7587
ACGT-flank	ACAACGTACG	0.7593	0.6860	0.9406	0.7109	0.7424	0.7681
ACGT-flank	ACAACGTACT	0.7436	0.8165	0.4602	0.3085	0.5581	0.5129

ACGT-flank	ACAACGTAGA	0.6721	0.7368	0.5948	0.7613	0.7911	0.7058
ACGT-flank	ACAACGTAGG	0.7468	0.9572	0.8480	0.8430	0.5451	1.0614
ACGT-flank	ACAACGTATG	0.3181	0.5462	0.5110	0.5163	0.5414	0.4334
ACGT-flank	ACAACGTCAG	0.7390	0.8708	0.7673	0.7825	0.6120	0.9135
ACGT-flank	ACAACGTCCA	0.6461	0.4674	1.1710	0.7352	0.6746	0.9586
ACGT-flank	ACAACGTCCG	0.9533	0.8286	0.7657	0.7313	0.6900	0.8498
ACGT-flank	ACAACGTCGC	0.9241	0.6016	0.9031	0.7732	0.5629	0.7381
ACGT-flank	ACAACGTCGG	0.8315	1.0141	0.7368	0.8223	0.8781	0.8193
ACGT-flank	ACAACGTCGT	0.6250	0.4081	0.7404	1.3908	0.8734	0.9924
ACGT-flank	ACAACGTCTG	0.7531	0.8953	0.5608	0.7417	0.8741	0.7601
ACGT-flank	ACAACGTGAC	0.7943	0.4860	0.8491	0.7248	0.7821	0.7237
ACGT-flank	ACAACGTGAG	0.9598	0.8189	0.8322	0.8506	0.8413	0.9392
ACGT-flank	ACAACGTGAT	0.7886	0.7275	0.7056	0.7629	0.8534	0.9205
ACGT-flank	ACAACGTGCA	1.2226	1.1425	1.0294	0.7186	0.9636	0.8898
ACGT-flank	ACAACGTGCC	0.8379	0.8622	1.0169	0.7671	0.8517	0.7739
ACGT-flank	ACAACGTGCG	1.1477	1.1610	1.2460	1.1184	1.0834	1.3587
ACGT-flank	ACAACGTGCT	0.7891	0.5816	0.4693	0.7592	0.7071	0.6859
ACGT-flank	ACAACGTGGA	0.7076	1.1118	0.7274	0.8044	0.7327	0.8039
ACGT-flank	ACAACGTGGC	0.9772	1.0620	0.9529	0.8266	1.0793	1.1240
ACGT-flank	ACAACGTGGG	1.3199	1.1347	1.1051	1.2784	1.2884	1.1359
ACGT-flank	ACAACGTGGT	0.7506	0.7711	0.9243	0.8440	0.7488	0.8530
ACGT-flank	ACAACGTGTA	0.8715	0.8698	0.8961	0.7668	0.8531	0.9211
ACGT-flank	ACAACGTGTC	0.8160	0.8771	0.7320	0.8625	1.0382	0.9181
ACGT-flank	ACAACGTGTG	1.1169	1.0663	1.2455	1.3291	1.3412	1.1669
ACGT-flank	ACAACGTTAG	0.5398	0.6063	0.4664	0.4936	0.6275	0.6107
ACGT-flank	ACAACGTTCC	0.9603	0.9060	0.7534	0.7924	0.8935	1.2698
ACGT-flank	ACAACGTTCG	0.9255	1.0541	1.1904	0.9525	1.0535	0.8908
ACGT-flank	ACAACGTTGA	0.4279	0.5588	0.4528	0.5248	0.5544	0.4513
ACGT-flank	ACAACGTTGG	0.9250	0.9999	1.1204	1.0668	0.9819	1.0577
ACGT-flank	ACCACGTAAA	0.9546	0.9262	0.8822	0.6829	0.6364	1.0631
ACGT-flank	ACCACGTAAG	0.6491	0.7263	0.7937	0.5836	0.5745	0.7432
ACGT-flank	ACCACGTACG	0.5582	0.9414	0.7734	0.7046	0.7402	0.7209
ACGT-flank	ACCACGTAGG	1.0682	1.0387	0.8160	0.9620	1.0467	0.9704
ACGT-flank	ACCACGTAGT	0.9261	0.6907	0.6406	0.9835	0.8092	0.8276
ACGT-flank	ACCACGTATC	1.1529	0.9053	1.0988	0.6949	0.5538	0.7128
ACGT-flank	ACCACGTCAC	1.2942	0.8947	0.8592	1.2437	1.0950	0.8458
ACGT-flank	ACCACGTCCA	0.5722	0.6432	0.6217	1.0681	0.9552	0.7141
ACGT-flank	ACCACGTCCG	0.7333	0.6288	0.7424	0.7265	0.6616	0.7408
ACGT-flank	ACCACGTCCT	0.9446	1.5271	1.2302	0.6860	0.9694	1.1094
ACGT-flank	ACCACGTCGA	0.7185	1.0281	0.8705	1.0090	0.8032	0.8653
ACGT-flank	ACCACGTCGC	0.5606	0.8184	0.8010	0.7797	0.7963	0.6493
ACGT-flank	ACCACGTCGG	1.0736	0.9839	0.8880	1.0640	1.0829	0.9704
ACGT-flank	ACCACGTCGT	0.9228	1.2749	0.9845	1.0826	0.9423	1.0290
ACGT-flank	ACCACGTCTG	0.7697	0.6924	0.8136	0.9130	0.8428	0.8724

ACGT-flank	ACCACGTGAC	0.6821	0.8208	0.7702	0.9511	0.8214	0.8213
ACGT-flank	ACCACGTGAG	0.9312	1.2105	0.9579	1.0792	0.9760	0.9334
ACGT-flank	ACCACGTGAT	1.0361	1.0372	1.2075	0.7701	0.9223	0.9051
ACGT-flank	ACCACGTGCG	1.1151	1.0575	1.1112	0.8569	1.0528	1.0896
ACGT-flank	ACCACGTGGA	0.9166	1.0631	1.0132	1.0495	0.9012	1.0056
ACGT-flank	ACCACGTGGC	1.7232	1.5437	1.6609	1.5815	1.6636	1.2581
ACGT-flank	ACCACGTGGG	1.3069	1.1652	1.3260	1.1869	1.2285	1.2358
ACGT-flank	ACCACGTGGT	0.7868	0.9791	0.8251	0.7494	0.9331	0.9982
ACGT-flank	ACCACGTGTA	0.8972	1.0257	0.8994	0.9149	0.9985	0.8604
ACGT-flank	ACCACGTGTC	1.5759	1.4370	1.3937	1.8993	1.7813	1.6678
ACGT-flank	ACCACGTGTG	0.9874	0.8306	0.8960	0.8615	1.1373	0.8507
ACGT-flank	ACCACGTTCG	0.9068	1.0546	0.9516	0.8802	0.9274	1.1274
ACGT-flank	ACCACGTTGA	0.8527	0.9134	0.9571	0.7441	0.7938	0.9183
ACGT-flank	ACCACGTTGC	0.8252	1.1302	0.8508	1.1932	0.9101	0.9956
ACGT-flank	ACCACGTTGG	0.8153	0.9272	0.8555	0.7637	0.8136	0.8659
ACGT-flank	ACGACGTAAA	0.9326	0.9454	0.9683	0.7008	1.2757	0.9524
ACGT-flank	ACGACGTAAC	0.6729	0.9080	0.6026	0.8911	0.8414	0.8408
ACGT-flank	ACGACGTAAG	1.0777	1.3383	0.7977	1.1334	0.9094	1.0625
ACGT-flank	ACGACGTAAT	0.5883	0.8093	0.7656	0.6685	0.6375	0.8836
ACGT-flank	ACGACGTACG	0.4328	0.7586	0.7932	0.7430	0.6819	0.7324
ACGT-flank	ACGACGTACT	0.6363	0.5624	0.5950	0.8697	0.7585	0.7956
ACGT-flank	ACGACGTAGA	1.0713	0.9226	1.0523	0.9358	0.8761	1.0321
ACGT-flank	ACGACGTAGG	1.0147	0.6913	0.8750	0.8540	0.9002	0.8143
ACGT-flank	ACGACGTAGT	0.8316	1.0649	0.8065	0.9236	0.7279	0.9625
ACGT-flank	ACGACGTATA	0.7772	1.1975	0.7230	0.9144	0.8849	0.6107
ACGT-flank	ACGACGTATC	1.1286	1.0880	1.0511	0.9262	1.1429	0.9617
ACGT-flank	ACGACGTATG	0.8313	1.2383	1.0217	0.8946	0.8650	0.9681
ACGT-flank	ACGACGTCAA	0.7644	0.8573	0.7614	1.1038	0.7835	0.8179
ACGT-flank	ACGACGTCAC	1.0505	0.9905	0.8537	0.6094	1.0503	1.0300
ACGT-flank	ACGACGTCAG	1.0480	0.9171	1.1506	1.2977	1.2479	1.0700
ACGT-flank	ACGACGTCAT	0.8221	0.6927	0.7633	0.8894	0.7333	0.7875
ACGT-flank	ACGACGTCCC	0.8868	1.5199	0.8249	0.8228	0.7912	0.6773
ACGT-flank	ACGACGTCCG	0.9127	1.0407	0.7674	0.8526	0.8633	0.9517
ACGT-flank	ACGACGTCCT	0.9363	1.0997	1.1575	0.9388	0.8558	0.9874
ACGT-flank	ACGACGTCGA	0.6279	0.7889	0.6962	0.8759	0.8516	0.8181
ACGT-flank	ACGACGTCGC	0.8030	0.9252	0.8663	0.8606	0.7703	0.6805
ACGT-flank	ACGACGTCGG	0.8071	0.6432	0.6492	0.7685	0.7097	0.7566
ACGT-flank	ACGACGTCGT	0.7978	0.7244	0.6809	0.7281	0.7071	0.7905
ACGT-flank	ACGACGTCTA	0.6865	0.3978	0.5257	1.0708	0.7558	0.7572
ACGT-flank	ACGACGTCTG	1.0314	1.0269	1.1982	1.0277	1.1544	1.0205
ACGT-flank	ACGACGTGAA	0.7029	0.7233	0.6755	0.7516	0.5877	0.6500
ACGT-flank	ACGACGTGAG	0.6637	0.9363	0.8240	0.5518	0.8091	0.7705
ACGT-flank	ACGACGTGAT	0.7103	0.7905	0.6710	0.8416	0.6776	0.7835
ACGT-flank	ACGACGTGCA	0.6972	1.0655	1.1013	0.7072	1.0540	0.9360

ACGT-flank	ACGACGTGCC	0.6484	0.5212	0.4332	0.7068	0.4971	0.5047
ACGT-flank	ACGACGTGCG	1.0090	0.9606	0.9369	0.6195	0.8814	0.9383
ACGT-flank	ACGACGTGCT	0.8916	0.9173	0.8503	0.8585	0.9199	0.9719
ACGT-flank	ACGACGTGGA	0.9666	0.8488	0.9279	1.0712	1.0579	1.0349
ACGT-flank	ACGACGTGGG	1.1436	0.8621	1.0953	1.0619	1.1107	1.0434
ACGT-flank	ACGACGTGGT	0.9419	0.8912	0.8830	0.7674	0.5957	0.7125
ACGT-flank	ACGACGTGTA	1.0479	0.8071	1.3212	0.7971	0.7126	0.9242
ACGT-flank	ACGACGTGTC	1.1207	1.0280	0.9953	1.2344	1.0090	1.0374
ACGT-flank	ACGACGTGTG	1.0566	0.9123	0.9810	1.0853	0.9840	1.0988
ACGT-flank	ACGACGTGTT	1.1340	1.6477	1.2723	1.5347	1.4389	1.3091
ACGT-flank	ACGACGTTAC	0.7320	0.7207	0.7464	0.8399	0.7731	0.8094
ACGT-flank	ACGACGTTAG	0.9382	0.9644	0.9297	0.8803	0.8360	1.0310
ACGT-flank	ACGACGTTCC	0.7689	0.8063	0.8462	0.7846	0.7863	0.8493
ACGT-flank	ACGACGTTCG	1.0000	0.8893	0.8496	0.9618	1.0623	1.0207
ACGT-flank	ACGACGTTGA	0.9576	1.0630	0.7030	0.9311	0.9174	0.9017
ACGT-flank	ACGACGTTGC	1.1185	0.9617	1.2758	1.0254	1.0891	1.1739
ACGT-flank	ACGACGTTGG	0.8976	0.8511	1.1363	0.9394	0.8117	0.8672
ACGT-flank	ACGACGTTGT	0.7850	0.9722	0.7068	1.0365	0.8564	0.9893
ACGT-flank	ACGACGTTTG	1.1330	1.2016	0.8372	0.8405	0.9961	0.7204
ACGT-flank	ACTACGTACG	0.6192	0.5573	0.7745	0.5808	0.6370	0.6026
ACGT-flank	ACTACGTAGA	0.9628	1.2123	0.9621	1.0147	1.0946	1.1455
ACGT-flank	ACTACGTAGC	0.7122	0.8298	0.8058	0.8056	0.7045	0.7275
ACGT-flank	ACTACGTAGG	1.1022	1.1771	1.1952	1.2578	0.9853	1.0548
ACGT-flank	ACTACGTCAG	0.8156	0.7772	0.8363	0.9727	0.8690	0.8317
ACGT-flank	ACTACGTCCG	1.0651	0.8411	0.9790	1.0042	1.0923	0.9838
ACGT-flank	ACTACGTCGA	0.5572	1.0162	0.8672	0.9437	0.8313	0.7893
ACGT-flank	ACTACGTCGC	0.7003	0.8602	0.7253	0.6648	0.8149	0.7474
ACGT-flank	ACTACGTCGG	0.9950	0.8948	0.9002	0.9830	0.9354	0.9940
ACGT-flank	ACTACGTCTG	0.9894	0.9871	0.7516	0.8658	1.0031	0.9723
ACGT-flank	ACTACGTGAA	0.9494	0.9916	0.7236	0.8381	0.9142	0.8839
ACGT-flank	ACTACGTGAG	1.1827	1.0244	1.1837	0.8562	1.2281	1.0525
ACGT-flank	ACTACGTGCA	0.9528	1.0283	0.7575	0.7669	0.9477	0.7948
ACGT-flank	ACTACGTGCC	1.0980	1.5677	1.0930	0.7912	1.1605	0.9856
ACGT-flank	ACTACGTGCG	0.9106	0.6799	0.8419	0.8567	0.8342	0.9170
ACGT-flank	ACTACGTGGA	0.7790	0.9502	0.9446	0.5767	0.8571	0.7546
ACGT-flank	ACTACGTGGC	1.0387	1.0615	1.2108	1.0710	1.1128	1.1265
ACGT-flank	ACTACGTGGG	1.2241	1.3368	1.2290	1.0655	1.2280	1.2401
ACGT-flank	ACTACGTGGT	0.7987	0.7629	0.7109	0.7732	0.6499	0.7198
ACGT-flank	ACTACGTGTG	0.9018	0.9459	0.9342	1.0762	1.0285	0.8870
ACGT-flank	ACTACGTTGG	1.2806	1.2742	1.2030	1.0802	1.1636	1.2835
ACGT-flank	AGAACGTAAA	0.9270	0.6354	0.6426	0.9319	0.6838	0.7397
ACGT-flank	AGAACGTAAC	0.7671	0.7909	0.7998	0.9348	0.8498	0.6858
ACGT-flank	AGAACGTAAG	0.7588	0.7465	0.7398	0.9076	0.7805	0.8461
ACGT-flank	AGAACGTACA	1.1017	1.0019	0.8846	1.0683	0.9680	0.8995

ACGT-flank	AGAACGTACC	0.6974	0.9642	0.9119	1.1198	1.0620	1.0791
ACGT-flank	AGAACGTACG	0.8576	0.9457	0.8969	0.7872	0.7771	0.9284
ACGT-flank	AGAACGTAGA	0.8744	0.7180	0.8055	0.8053	0.9620	0.7748
ACGT-flank	AGAACGTAGG	1.1116	1.0644	1.2474	1.2265	0.9970	1.1400
ACGT-flank	AGAACGTAGT	0.9157	1.0489	1.0007	0.8221	0.9625	1.0893
ACGT-flank	AGAACGTATG	0.8461	0.8612	0.8653	0.5720	0.7698	0.8808
ACGT-flank	AGAACGTCAA	0.8408	0.7552	0.7689	0.7252	0.6833	0.7534
ACGT-flank	AGAACGTCAG	0.8524	0.8767	0.6475	0.8296	0.7650	0.7412
ACGT-flank	AGAACGTCCC	0.6617	0.6633	0.4388	1.1485	0.6740	0.8598
ACGT-flank	AGAACGTCCT	1.3606	1.0750	0.9621	0.9410	1.0130	1.0617
ACGT-flank	AGAACGTCGA	0.8134	0.8359	0.7416	0.6087	0.5906	0.7341
ACGT-flank	AGAACGTCGG	0.9741	1.0986	1.0875	1.0034	0.9258	1.0200
ACGT-flank	AGAACGTCGT	0.7090	0.7865	0.7523	0.5218	0.3065	0.7897
ACGT-flank	AGAACGTCTG	1.0722	0.9534	0.9603	0.9519	0.9533	0.9458
ACGT-flank	AGAACGTGAA	0.6361	0.6606	0.8056	0.8888	0.6481	0.7653
ACGT-flank	AGAACGTGAC	0.7038	0.6303	1.2742	0.9435	0.8174	0.6581
ACGT-flank	AGAACGTGAG	0.9565	0.9191	0.9398	1.0039	0.9485	0.9318
ACGT-flank	AGAACGTGAT	0.8028	0.7982	0.6853	0.8305	0.8910	0.7790
ACGT-flank	AGAACGTGCG	0.7429	0.6722	0.8385	0.8629	0.7915	0.9523
ACGT-flank	AGAACGTGGA	0.9814	0.8681	0.8798	0.8791	0.8982	0.8537
ACGT-flank	AGAACGTGGC	0.8207	0.7013	0.8815	0.9728	0.7384	0.8280
ACGT-flank	AGAACGTGGG	1.5666	1.4363	1.6368	1.5983	1.6362	1.5736
ACGT-flank	AGAACGTGGT	0.9876	0.8957	1.1943	0.8890	0.8464	0.8985
ACGT-flank	AGAACGTGTA	0.9713	1.1721	0.9106	1.0286	0.9967	0.9971
ACGT-flank	AGAACGTGTC	1.0670	0.8215	0.8183	0.9071	0.9041	1.0084
ACGT-flank	AGAACGTGTG	1.1770	1.1461	1.2982	1.5096	1.5675	1.4380
ACGT-flank	AGAACGTGTT	1.1605	1.2763	1.4963	1.0532	1.1623	1.1473
ACGT-flank	AGAACGTTAA	1.1631	1.0993	1.0128	1.1007	1.1834	1.0824
ACGT-flank	AGAACGTTAG	0.6548	0.6892	0.6862	0.8041	0.6954	0.6925
ACGT-flank	AGAACGTTCC	0.5465	0.8988	0.7763	0.6800	0.6828	0.7715
ACGT-flank	AGAACGTTCG	1.3148	1.2917	1.1579	1.1340	1.1620	1.3219
ACGT-flank	AGAACGTTGA	0.7262	0.8835	0.7516	0.6982	0.7699	0.9113
ACGT-flank	AGAACGTTGC	0.8042	0.8984	0.6240	0.8039	0.6517	0.6651
ACGT-flank	AGAACGTTGG	1.0866	1.1223	1.0235	1.1198	1.0678	1.1639
ACGT-flank	AGAACGTTGT	1.2270	1.1629	1.3162	0.9882	1.1031	1.0681
ACGT-flank	AGCACGTAAA	0.6335	1.1991	0.7713	0.8631	0.7872	0.7811
ACGT-flank	AGCACGTAAC	0.7999	0.7982	1.0860	0.5283	0.6457	0.8201
ACGT-flank	AGCACGTAAG	0.6171	0.8187	0.7839	0.7417	0.7662	0.7963
ACGT-flank	AGCACGTAAT	1.2616	1.1390	0.6626	0.8734	1.4685	0.9164
ACGT-flank	AGCACGTACG	0.7840	0.8733	0.9425	0.8458	0.7787	0.7872
ACGT-flank	AGCACGTAGA	0.9630	0.8493	0.9479	0.8843	0.8715	0.8473
ACGT-flank	AGCACGTAGG	0.9599	1.2024	1.0867	1.0037	1.0439	1.0613
ACGT-flank	AGCACGTAGT	0.7869	0.7502	0.7002	0.8339	0.9054	0.6934
ACGT-flank	AGCACGTATC	0.8020	0.6115	0.6633	0.8295	0.7265	0.7728

ACGT-flank	AGCACGTCAA	0.8243	0.3325	1.0634	0.4382	0.6994	0.4397
ACGT-flank	AGCACGTCAC	0.8634	0.7415	0.8040	0.9071	0.7358	0.8914
ACGT-flank	AGCACGTCAG	1.0568	0.9343	0.9429	0.9216	0.9282	1.0083
ACGT-flank	AGCACGTCAT	0.9732	0.8154	0.9927	1.0125	0.9112	1.1066
ACGT-flank	AGCACGTCCC	0.8499	0.7324	1.0861	0.6335	0.7624	0.5735
ACGT-flank	AGCACGTCCG	0.6972	0.5212	0.5729	0.8728	0.6340	0.7779
ACGT-flank	AGCACGTCGG	1.2283	1.2951	1.3378	1.0603	1.2324	1.1957
ACGT-flank	AGCACGTCGT	1.5766	1.2592	1.3490	1.6706	1.2982	1.5541
ACGT-flank	AGCACGTCTG	0.7475	1.0053	0.8420	0.8896	0.8136	0.9067
ACGT-flank	AGCACGTGAA	1.1806	0.9344	0.8437	0.6496	1.1313	1.0244
ACGT-flank	AGCACGTGAC	1.2813	1.0710	1.2360	0.9792	0.9766	1.1642
ACGT-flank	AGCACGTGAG	0.9233	0.8510	0.9488	0.8614	0.8894	0.8638
ACGT-flank	AGCACGTGAT	1.0152	1.1257	1.0210	1.1008	1.0802	1.1020
ACGT-flank	AGCACGTGCA	0.7377	0.8255	0.9638	0.6570	0.8237	0.6060
ACGT-flank	AGCACGTGCC	0.5835	1.2090	1.1936	0.5218	0.9080	0.6734
ACGT-flank	AGCACGTGCG	0.8671	0.7257	0.8793	0.8485	1.2485	0.8825
ACGT-flank	AGCACGTGGA	0.9771	0.7626	0.9935	0.9256	0.8552	0.9493
ACGT-flank	AGCACGTGGG	1.1476	1.2938	1.0313	1.0704	1.2365	1.1584
ACGT-flank	AGCACGTGGT	0.8621	0.8886	0.9318	0.9676	0.9726	0.9467
ACGT-flank	AGCACGTGTA	0.9865	0.9345	1.1205	0.8893	1.0592	1.0813
ACGT-flank	AGCACGTGTC	1.1169	1.1900	1.1808	1.2372	1.5223	1.2154
ACGT-flank	AGCACGTGTG	1.4510	0.9757	1.2367	1.2354	1.1863	1.2113
ACGT-flank	AGCACGTGTT	0.7571	0.9328	0.7856	0.7019	0.6541	0.5107
ACGT-flank	AGCACGTTAG	0.9333	1.1158	1.1020	0.7520	0.9176	0.9616
ACGT-flank	AGCACGTTCC	0.8148	0.8846	0.5179	0.8168	0.7272	0.7175
ACGT-flank	AGCACGTTCG	0.8767	0.8746	0.9032	0.9251	0.8151	0.7473
ACGT-flank	AGCACGTTGA	0.9307	1.0660	0.8092	0.8501	1.0229	0.9960
ACGT-flank	AGCACGTTGC	0.5740	0.5228	0.6285	0.7545	0.4843	0.6521
ACGT-flank	AGCACGTTGG	0.8595	0.9576	0.9939	0.9146	0.8546	0.8832
ACGT-flank	AGCACGTTGT	0.5320	0.9821	0.8228	0.7231	0.7807	0.7255
ACGT-flank	AGCACGTTTG	0.6635	1.0882	0.8515	1.0402	0.6550	0.8906
ACGT-flank	AGGACGTAAA	0.8390	1.0262	1.0690	0.9625	0.9145	0.8824
ACGT-flank	AGGACGTAAC	0.8004	0.9003	0.6665	0.6704	0.6727	0.8213
ACGT-flank	AGGACGTAAG	0.7207	0.8597	0.6947	0.6892	0.9173	0.7505
ACGT-flank	AGGACGTAAT	1.1468	1.3525	1.1832	0.9243	1.0341	1.0470
ACGT-flank	AGGACGTACA	0.8290	0.9214	0.7526	0.7156	0.8261	0.8044
ACGT-flank	AGGACGTACC	0.5347	0.7972	0.7491	0.6239	0.7570	0.8695
ACGT-flank	AGGACGTACG	0.6480	0.7602	0.5597	0.6222	0.6230	0.7201
ACGT-flank	AGGACGTACT	0.7205	0.8292	0.7806	0.7220	0.8143	0.7264
ACGT-flank	AGGACGTAGA	0.8350	0.9177	0.9999	0.9097	1.0554	0.9451
ACGT-flank	AGGACGTAGC	0.6671	0.9749	0.7050	0.7269	0.7388	0.8142
ACGT-flank	AGGACGTAGG	0.8819	0.9693	0.9454	1.2465	0.9939	1.0348
ACGT-flank	AGGACGTAGT	0.8116	0.7649	0.7664	0.9610	0.8246	0.8536
ACGT-flank	AGGACGTATA	0.7832	0.7621	1.0128	0.8515	0.8723	0.8676

ACGT-flank	AGGACGTATC	0.5939	0.5463	0.8492	0.6867	0.5362	0.5623
ACGT-flank	AGGACGTATG	0.7910	0.8389	0.7797	0.9002	0.8530	0.7737
ACGT-flank	AGGACGTCAA	1.1066	0.9613	0.9234	0.9553	0.9121	1.0575
ACGT-flank	AGGACGTCAC	1.0113	0.6796	0.9253	0.6223	0.6352	1.2241
ACGT-flank	AGGACGTCAG	0.6946	0.6913	0.7621	0.6395	0.8155	0.8052
ACGT-flank	AGGACGTCAT	0.7565	0.7056	0.8260	0.8851	0.8329	0.6544
ACGT-flank	AGGACGTCCA	1.0337	0.8255	1.0095	0.9337	0.7760	1.1004
ACGT-flank	AGGACGTCCG	1.4332	1.1619	0.7928	0.5468	1.0798	0.7952
ACGT-flank	AGGACGTCCT	0.7847	0.8194	0.8602	0.8249	0.7986	0.8359
ACGT-flank	AGGACGTCGA	0.7054	0.5577	0.6244	0.6270	0.6275	0.6433
ACGT-flank	AGGACGTCGC	1.0235	0.8357	0.8939	0.7963	0.8821	0.7938
ACGT-flank	AGGACGTCGG	1.0920	1.2048	0.8744	0.9574	0.9641	0.9515
ACGT-flank	AGGACGTCGT	0.5271	0.7417	0.5378	0.5460	0.6463	0.6192
ACGT-flank	AGGACGTCTA	0.9077	0.8194	0.8977	1.0648	1.0914	0.9489
ACGT-flank	AGGACGTCTC	0.8385	0.7059	0.6572	0.5978	0.7313	0.8144
ACGT-flank	AGGACGTCTG	0.8781	0.8250	0.9308	0.8657	0.9112	0.9152
ACGT-flank	AGGACGTGAA	0.8118	0.7513	0.6403	0.5683	0.7926	0.5494
ACGT-flank	AGGACGTGAC	1.1908	0.8421	0.9100	1.1972	0.9674	0.8306
ACGT-flank	AGGACGTGAG	0.7746	0.8513	0.7108	0.7416	0.7121	0.8336
ACGT-flank	AGGACGTGAT	0.9380	0.9603	0.9349	0.9569	1.0917	0.8108
ACGT-flank	AGGACGTGCA	0.8567	0.7693	0.7513	0.8237	0.7200	0.8051
ACGT-flank	AGGACGTGCC	0.6490	0.5452	0.4005	0.2569	0.6147	0.3881
ACGT-flank	AGGACGTGCG	0.7767	0.5537	0.5149	0.6458	0.7687	0.8479
ACGT-flank	AGGACGTGCT	0.9308	0.8842	1.0476	0.9462	1.0117	0.8654
ACGT-flank	AGGACGTGGA	1.0632	1.0093	0.9646	0.9902	1.0982	1.0017
ACGT-flank	AGGACGTGGC	1.3124	0.6898	1.3037	0.9917	0.8031	0.9726
ACGT-flank	AGGACGTGGG	1.0305	0.9470	1.1221	1.1567	1.1810	1.1416
ACGT-flank	AGGACGTGGT	0.8734	0.8268	0.8788	0.8858	0.8573	0.9470
ACGT-flank	AGGACGTGTA	1.2260	1.0596	0.8506	0.8501	0.8553	0.9116
ACGT-flank	AGGACGTGTC	0.8616	1.2620	1.1354	1.0731	1.1619	0.9903
ACGT-flank	AGGACGTGTG	1.1136	0.9836	1.0521	1.1789	1.0865	1.1335
ACGT-flank	AGGACGTGTT	1.1643	0.9485	0.9876	0.9946	1.0992	1.0629
ACGT-flank	AGGACGTTAA	1.0037	0.6667	1.0445	0.6692	0.8823	0.8146
ACGT-flank	AGGACGTTAC	0.8142	0.8600	1.0886	0.8717	0.7455	0.8871
ACGT-flank	AGGACGTTAG	0.7459	0.8473	0.6694	0.7493	0.7453	0.7484
ACGT-flank	AGGACGTTCA	0.9925	0.9071	1.0354	0.7503	0.8854	0.9437
ACGT-flank	AGGACGTTCC	0.9982	1.0033	1.1764	1.2730	1.1380	1.2257
ACGT-flank	AGGACGTTCG	0.6372	0.6213	0.5806	0.6361	0.7452	0.7563
ACGT-flank	AGGACGTTCT	0.8302	0.7489	0.7615	0.5829	0.9518	0.8906
ACGT-flank	AGGACGTTGA	0.7904	0.7971	0.7239	0.7209	0.8003	0.7336
ACGT-flank	AGGACGTTGC	0.7408	0.8006	0.6943	0.7725	0.6866	0.7090
ACGT-flank	AGGACGTTGG	0.8581	0.9446	0.8930	0.9070	0.8735	0.9840
ACGT-flank	AGGACGTTGT	0.8483	0.7804	0.7519	0.7974	0.7357	0.7977
ACGT-flank	AGGACGTTTG	0.9635	1.1369	1.0588	1.1419	1.0350	1.0759

ACGT-flank	AGTACGTAAA	0.8487	0.9331	0.9104	0.9833	0.9673	0.7983
ACGT-flank	AGTACGTAAG	0.8095	0.8624	0.8974	0.7170	0.8736	1.0236
ACGT-flank	AGTACGTACC	0.8135	0.7033	0.7370	0.7221	0.8061	0.7739
ACGT-flank	AGTACGTACG	0.9385	1.0509	1.0073	0.7888	0.7291	0.7968
ACGT-flank	AGTACGTAGA	0.8466	0.7440	0.8387	0.7842	0.8079	0.7932
ACGT-flank	AGTACGTAGC	0.8817	1.0895	0.8482	0.9833	0.7715	0.8552
ACGT-flank	AGTACGTAGG	1.0836	0.9168	1.1534	1.0293	1.1255	1.1435
ACGT-flank	AGTACGTAGT	0.9667	0.8520	0.8317	0.9660	0.9244	0.8806
ACGT-flank	AGTACGTATG	0.8674	1.2419	1.0843	1.2456	1.1841	1.0776
ACGT-flank	AGTACGTCAC	1.2853	1.1976	1.5865	1.3023	1.0612	0.5531
ACGT-flank	AGTACGTCAG	0.8918	0.8201	0.8052	0.9816	0.9519	1.0121
ACGT-flank	AGTACGTCCA	0.6324	0.6746	0.8711	0.7180	0.8230	0.7776
ACGT-flank	AGTACGTCCC	0.6968	0.7463	0.8538	0.8942	0.7974	0.8002
ACGT-flank	AGTACGTCCG	0.9466	0.9151	1.0009	0.7839	0.9643	0.9430
ACGT-flank	AGTACGTCCT	0.6092	0.6237	0.6021	0.8968	0.8148	0.7059
ACGT-flank	AGTACGTCGA	0.8287	0.8783	0.9676	0.7567	0.8422	0.8021
ACGT-flank	AGTACGTCGC	0.6605	0.7810	0.8178	0.6538	0.7800	0.7923
ACGT-flank	AGTACGTCGG	0.8250	1.0816	1.0139	0.8482	0.8862	0.9170
ACGT-flank	AGTACGTCTG	1.0904	0.9806	0.7657	0.7839	0.9618	1.0276
ACGT-flank	AGTACGTGAA	0.5424	0.8622	0.7735	0.8036	0.7606	0.6255
ACGT-flank	AGTACGTGAC	0.7836	0.7599	0.6380	0.7684	0.7298	0.8924
ACGT-flank	AGTACGTGAG	0.8252	0.7124	0.8041	0.7998	0.8628	0.7964
ACGT-flank	AGTACGTGAT	0.9139	1.2492	0.5678	1.1551	0.9515	1.0376
ACGT-flank	AGTACGTGCA	0.9363	0.9346	1.0263	0.5403	0.9132	0.8820
ACGT-flank	AGTACGTGCC	0.7112	0.6984	0.6372	0.7361	0.6385	0.5561
ACGT-flank	AGTACGTGCG	1.0018	1.0729	1.0986	0.9507	0.8456	1.0359
ACGT-flank	AGTACGTGCT	1.1933	0.9331	0.8983	0.8178	0.8472	0.9063
ACGT-flank	AGTACGTGGA	0.7795	0.9994	0.8120	0.7747	0.8564	0.9117
ACGT-flank	AGTACGTGGC	0.9468	1.0369	1.2694	1.1122	1.0231	0.9481
ACGT-flank	AGTACGTGGG	1.4174	1.3698	1.3536	1.4704	1.4263	1.3717
ACGT-flank	AGTACGTGGT	0.9604	0.9359	1.1256	0.9850	1.1089	1.0606
ACGT-flank	AGTACGTGTC	1.0348	1.3815	1.2312	1.1825	1.1348	1.2728
ACGT-flank	AGTACGTGTG	1.2512	0.8280	1.1719	1.0225	1.2447	1.3322
ACGT-flank	AGTACGTTCG	1.1366	1.0306	1.2643	1.0148	0.9108	0.9922
ACGT-flank	AGTACGTTGG	0.8243	0.9683	0.9361	0.8471	0.9425	0.9384
ACGT-flank	ATAACGTACG	0.8570	0.6107	0.8531	0.8095	0.7665	0.7750
ACGT-flank	ATAACGTAGC	0.9727	1.0399	0.7566	0.8407	0.8970	1.0386
ACGT-flank	ATAACGTAGG	1.0674	0.9905	1.2718	1.1357	1.2773	1.1656
ACGT-flank	ATAACGTCAG	0.8040	0.6831	0.9283	0.7196	0.8729	0.8692
ACGT-flank	ATAACGTCCG	0.8189	1.0273	0.7718	1.0589	0.8074	0.7934
ACGT-flank	ATAACGTCGA	0.6336	0.5801	0.7974	0.6881	0.6115	0.6290
ACGT-flank	ATAACGTCGG	0.9457	0.9857	0.8572	0.7130	0.8769	0.8942
ACGT-flank	ATAACGTGAG	1.0144	1.0664	1.0246	0.9657	1.0336	0.9078
ACGT-flank	ATAACGTGCA	0.8166	0.9563	0.7050	0.7173	0.9416	0.9344

ACGT-flank	ATAACGTGCC	0.6118	0.9029	0.6186	0.4615	0.7866	0.8527
ACGT-flank	ATAACGTGCG	0.9074	0.7047	0.7977	0.7123	0.7359	0.7876
ACGT-flank	ATAACGTGGA	0.7810	0.7816	0.7834	0.6727	0.7795	0.8237
ACGT-flank	ATAACGTGGC	0.8963	0.8080	0.9952	0.7137	0.8053	1.0045
ACGT-flank	ATAACGTGGG	1.3282	1.2672	1.2384	1.4786	1.4537	1.3088
ACGT-flank	ATAACGTGTC	1.0901	1.2243	1.3913	1.4691	0.9879	1.1011
ACGT-flank	ATAACGTGTG	1.4651	1.2087	1.5712	1.6073	1.4949	1.2398
ACGT-flank	ATCACGTAGC	0.8636	0.9372	0.9771	0.7737	0.8903	1.0529
ACGT-flank	ATCACGTAGG	1.0512	1.1087	1.2356	1.0136	0.9871	1.1471
ACGT-flank	ATCACGTCCC	1.0508	0.9773	0.8850	0.8938	1.0404	1.0519
ACGT-flank	ATCACGTCCG	0.8462	0.8206	0.9078	0.7711	0.8834	0.9307
ACGT-flank	ATCACGTCGC	0.9021	0.8681	0.8172	0.9518	0.9638	0.9373
ACGT-flank	ATCACGTCGG	0.9455	0.7664	1.0664	0.9781	0.9477	0.9643
ACGT-flank	ATCACGTGAG	1.0006	0.8288	0.7044	0.9293	0.8166	0.5749
ACGT-flank	ATCACGTGCG	0.8983	0.7436	0.9601	0.8500	0.8017	0.8076
ACGT-flank	ATCACGTGGA	0.6682	1.0424	0.6428	0.6429	0.5972	0.6011
ACGT-flank	ATCACGTGGC	0.7899	0.9202	0.9983	0.8549	0.9441	0.9794
ACGT-flank	ATCACGTGGG	1.3459	1.3510	1.1614	1.1969	1.2665	1.1329
ACGT-flank	ATCACGTGGT	0.8773	0.7601	0.9134	0.8647	0.8670	0.8974
ACGT-flank	ATCACGTGTG	1.2464	1.2799	1.2263	1.2670	1.4630	1.3316
ACGT-flank	ATGACGTAAA	0.7632	0.8087	0.8992	0.8294	0.8056	0.8783
ACGT-flank	ATGACGTAAC	0.7090	0.8243	1.0396	0.9397	0.9219	1.0554
ACGT-flank	ATGACGTAAG	0.5946	0.7881	0.7846	0.5935	0.7980	0.7310
ACGT-flank	ATGACGTACC	0.7770	0.7721	0.8222	0.8137	0.8579	1.0284
ACGT-flank	ATGACGTACG	0.5810	0.8857	0.6134	0.7673	0.7420	0.6923
ACGT-flank	ATGACGTAGA	1.1347	0.9727	1.2763	0.7926	1.1734	1.0117
ACGT-flank	ATGACGTAGC	1.1660	1.0222	0.9757	0.8726	0.8709	1.0737
ACGT-flank	ATGACGTAGG	1.0464	1.0125	0.9966	1.0653	1.0359	1.0894
ACGT-flank	ATGACGTAGT	0.9000	1.1934	1.1342	1.2310	0.8843	0.9493
ACGT-flank	ATGACGTCAC	0.8725	0.8158	0.6669	0.6607	0.6994	0.8831
ACGT-flank	ATGACGTCAG	0.8349	0.7788	0.8377	0.7375	0.8126	0.6906
ACGT-flank	ATGACGTCCA	1.3016	0.9138	0.8582	1.0053	1.1931	1.1426
ACGT-flank	ATGACGTCCC	0.8367	0.5819	0.6618	0.5297	0.6832	0.5463
ACGT-flank	ATGACGTCCG	0.9139	0.8127	0.8288	0.7071	0.8930	0.8495
ACGT-flank	ATGACGTCGA	0.7916	0.6015	0.7854	0.6117	0.7502	0.6739
ACGT-flank	ATGACGTCGC	0.6890	1.0339	0.5935	0.6473	0.4988	0.6918
ACGT-flank	ATGACGTCGG	0.8270	0.6916	0.7213	0.7507	0.7353	0.8069
ACGT-flank	ATGACGTCGT	0.8332	0.7643	0.7709	0.8409	0.9179	0.7709
ACGT-flank	ATGACGTCTA	0.9048	1.2666	0.7630	0.6768	0.9228	0.7566
ACGT-flank	ATGACGTCTG	0.7017	0.6142	0.6664	0.6578	0.7184	0.6433
ACGT-flank	ATGACGTGAA	0.7885	0.7726	0.8931	0.8900	0.7311	0.8792
ACGT-flank	ATGACGTGAC	0.9864	1.0733	0.9661	1.0359	1.2228	0.9048
ACGT-flank	ATGACGTGAG	1.3235	0.8876	1.1901	1.1883	1.1811	1.1624
ACGT-flank	ATGACGTGAT	0.8082	0.7662	1.0452	0.6771	0.7725	0.7160

ACGT-flank	ATGACGTGCA	0.6459	0.7360	0.5513	0.6837	0.6471	0.7810
ACGT-flank	ATGACGTGCC	0.7208	0.4454	0.6036	0.4841	0.6246	0.6198
ACGT-flank	ATGACGTGCG	0.6982	0.7061	0.7977	0.5898	0.6426	0.8056
ACGT-flank	ATGACGTGCT	0.6006	0.5928	0.6536	0.6590	0.5921	0.5663
ACGT-flank	ATGACGTGGA	0.9743	1.1623	0.9942	1.0010	0.9690	1.0298
ACGT-flank	ATGACGTGGC	1.0201	1.1264	0.9665	1.1111	1.1247	1.1602
ACGT-flank	ATGACGTGGG	1.1055	1.1895	1.1888	1.1842	1.0621	1.1237
ACGT-flank	ATGACGTGGT	1.2187	1.0935	1.1801	1.0619	1.1738	1.1084
ACGT-flank	ATGACGTGTA	0.9471	0.8729	0.6813	1.1720	1.0434	0.9950
ACGT-flank	ATGACGTGTC	1.3095	1.0603	1.0791	1.3135	1.2514	1.0386
ACGT-flank	ATGACGTGTG	1.1158	0.9405	1.0589	1.1055	1.1121	1.2043
ACGT-flank	ATGACGTTCG	0.5487	0.7984	0.7058	0.6978	0.7439	0.6174
ACGT-flank	ATGACGTTGA	0.8322	0.9133	0.8313	0.6616	0.6103	0.7937
ACGT-flank	ATGACGTTGC	0.7155	0.7841	0.8843	0.8083	0.8240	0.9018
ACGT-flank	ATGACGTTGG	0.7856	0.7939	0.7312	0.7730	0.8094	0.7333
ACGT-flank	ATTACGTGGC	1.1917	1.3188	1.2468	1.4103	1.2681	1.3341
ACGT-flank	ATTACGTGGG	1.3015	1.2573	1.3091	1.5391	1.2534	1.3393
ACGT-flank	CAAACGTAAA	1.0550	1.1207	1.0493	1.2135	1.0991	0.9679
ACGT-flank	CAAACGTAAC	0.6971	0.9555	0.9655	0.6995	0.8586	0.7950
ACGT-flank	CAAACGTAAG	1.0697	1.0822	1.2823	1.0717	1.4294	1.0672
ACGT-flank	CAAACGTACA	0.9166	0.7330	0.6584	0.7221	0.7358	0.7956
ACGT-flank	CAAACGTACG	0.9058	0.6243	0.7264	0.6896	0.8349	0.8673
ACGT-flank	CAAACGTAGA	1.1777	0.7639	1.1729	0.6406	1.0137	0.8876
ACGT-flank	CAAACGTAGC	1.0829	1.0089	0.8785	1.0509	0.7489	1.2360
ACGT-flank	CAAACGTAGG	1.0133	0.9909	1.0197	0.9821	0.8819	0.8527
ACGT-flank	CAAACGTAGT	1.0484	0.9874	0.9340	0.9182	0.9832	1.0387
ACGT-flank	CAAACGTATG	1.0527	1.0047	0.8889	0.6879	0.7739	0.7783
ACGT-flank	CAAACGTCAG	0.8569	0.9210	1.0578	0.8284	0.9231	0.9513
ACGT-flank	CAAACGTCAT	0.9719	0.5519	0.4149	0.2564	0.4409	0.4769
ACGT-flank	CAAACGTCCA	0.9328	0.9991	0.7901	0.5913	0.6247	0.7376
ACGT-flank	CAAACGTCCC	0.7349	0.8982	1.0721	1.0287	0.8961	0.9443
ACGT-flank	CAAACGTCCG	0.9063	0.8336	0.6731	0.7827	0.7868	0.7278
ACGT-flank	CAAACGTCGA	0.8629	0.7732	0.7878	0.6254	0.7196	0.7476
ACGT-flank	CAAACGTCGG	0.9069	0.8838	0.8553	1.0772	0.9464	0.9614
ACGT-flank	CAAACGTCGT	0.8605	0.9408	1.0553	1.0132	0.9150	0.8591
ACGT-flank	CAAACGTGAA	0.7885	0.9607	0.8968	0.7159	0.6112	0.7799
ACGT-flank	CAAACGTGAC	0.5675	0.6008	1.0446	0.6698	0.6666	0.7066
ACGT-flank	CAAACGTGAG	0.7565	0.6890	0.7493	0.8041	0.8415	0.7162
ACGT-flank	CAAACGTGAT	0.6328	1.0079	0.6783	0.7191	0.8744	0.8419
ACGT-flank	CAAACGTGCA	1.1086	1.0548	0.8904	0.7140	0.6920	0.8241
ACGT-flank	CAAACGTGCC	0.8554	0.9784	0.3655	0.9857	0.5587	0.3162
ACGT-flank	CAAACGTGCG	0.8983	1.1026	0.9191	0.7280	0.9405	0.8593
ACGT-flank	CAAACGTGCT	0.9864	1.0847	0.6251	1.0241	0.7946	1.0041
ACGT-flank	CAAACGTGGA	0.6954	0.6883	0.6950	0.8000	0.7065	0.6147

ACGT-flank	CAAACGTGGC	0.8926	1.1097	1.0137	1.2641	1.2645	0.8712
ACGT-flank	CAAACGTGGG	1.2221	1.1178	1.4097	1.5601	1.3679	1.2376
ACGT-flank	CAAACGTGGT	0.9514	0.9420	0.6585	0.8220	0.8003	0.8208
ACGT-flank	CAAACGTGTA	1.0182	0.5652	0.8946	1.0501	0.8528	0.8977
ACGT-flank	CAAACGTGTC	0.8554	0.9896	1.0127	0.8619	0.8831	0.8930
ACGT-flank	CAAACGTGTG	1.2926	1.0125	1.2605	1.4234	1.1630	1.1944
ACGT-flank	CAAACGTTCG	0.7609	0.7367	1.0099	0.9191	0.9982	0.8650
ACGT-flank	CAAACGTTGA	0.7641	0.6269	1.0015	0.6496	0.9406	0.9547
ACGT-flank	CAAACGTTGC	0.8192	0.9700	0.9280	0.8815	1.0689	0.8820
ACGT-flank	CAAACGTTGG	0.9714	0.8435	0.9521	1.0379	1.0231	1.0520
ACGT-flank	CAAACGTTGT	0.9832	0.8484	0.7752	0.9754	1.0591	0.9306
ACGT-flank	CAAACGTTTG	0.8029	0.9715	0.9409	0.8333	0.9683	0.8989
ACGT-flank	CACACGTAAA	0.9699	0.8146	0.6969	0.8042	0.7636	0.8079
ACGT-flank	CACACGTACG	0.4603	0.6143	0.4617	0.5595	0.5959	0.4771
ACGT-flank	CACACGTAGA	0.8080	0.9742	0.8480	0.9431	0.9816	0.9298
ACGT-flank	CACACGTAGC	0.8459	0.8620	0.8444	0.8058	0.7340	0.8587
ACGT-flank	CACACGTAGG	1.2416	0.9920	1.0512	1.2028	1.2126	1.2790
ACGT-flank	CACACGTATG	0.7769	1.0687	0.8365	0.9702	0.7889	0.7153
ACGT-flank	CACACGTCAG	1.0363	1.1507	0.8096	1.1925	0.8421	0.8351
ACGT-flank	CACACGTCCA	0.9301	1.3737	1.1819	0.9365	0.9489	1.0205
ACGT-flank	CACACGTCCC	0.9975	1.0058	1.0105	0.6809	1.0692	0.9784
ACGT-flank	CACACGTCCG	0.9403	0.8686	1.0099	0.6726	0.6633	1.0032
ACGT-flank	CACACGTCCT	0.9217	1.1098	1.0632	0.9140	0.9405	0.9195
ACGT-flank	CACACGTCGA	0.9058	0.7954	0.7481	0.8055	0.9271	0.9004
ACGT-flank	CACACGTCGC	0.8815	0.8971	0.8339	0.8876	0.5668	0.9432
ACGT-flank	CACACGTCGG	0.9670	0.9784	0.9096	0.9394	0.7205	0.8532
ACGT-flank	CACACGTCGT	0.8399	0.8245	0.6972	0.7538	0.8140	0.9344
ACGT-flank	CACACGTCTG	0.9867	0.7150	0.9350	0.9356	0.8664	0.8668
ACGT-flank	CACACGTGAA	1.3046	1.3768	1.0501	1.2226	1.4594	1.0868
ACGT-flank	CACACGTGAG	0.9948	0.7997	0.8997	0.8420	0.9085	0.9099
ACGT-flank	CACACGTGCA	1.0507	1.2171	0.9061	0.9473	1.0497	1.0030
ACGT-flank	CACACGTGCC	0.6734	0.6136	0.6633	0.8165	0.8610	0.6920
ACGT-flank	CACACGTGCG	0.8698	0.9079	1.0396	1.0831	1.1012	1.0840
ACGT-flank	CACACGTGCT	1.1698	0.9471	1.0912	1.0373	1.0672	1.0065
ACGT-flank	CACACGTGGA	0.9695	1.1184	0.9309	1.1374	0.8922	0.9586
ACGT-flank	CACACGTGGC	1.3337	1.3374	1.1029	1.4411	1.5538	1.3556
ACGT-flank	CACACGTGGG	1.5861	1.4625	1.4279	1.4793	1.5299	1.4712
ACGT-flank	CACACGTGGT	0.8707	0.8473	0.8393	0.9091	0.9013	0.9040
ACGT-flank	CACACGTGTA	0.9942	1.4485	1.4401	1.2193	1.2792	1.1909
ACGT-flank	CACACGTGTG	1.1256	1.2964	1.2195	1.1947	1.2860	1.0909
ACGT-flank	CACACGTTCC	0.4645	1.3533	0.4983	0.5666	0.6437	0.8924
ACGT-flank	CACACGTTCG	1.5675	1.6559	1.3750	1.2877	1.4029	1.3019
ACGT-flank	CACACGTTGA	1.1535	1.0951	0.9232	1.1128	0.9927	1.0089
ACGT-flank	CACACGTTGC	0.9010	0.7471	0.7262	0.7275	0.8307	0.8098

ACGT-flank	CACACGTTGG	1.1240	1.0167	1.0274	1.1515	0.9467	1.1730
ACGT-flank	CAGACGTAAC	0.8203	0.7868	0.7381	0.8503	0.9938	0.9297
ACGT-flank	CAGACGTAAG	0.9930	1.1652	0.7375	0.8909	0.8974	0.9545
ACGT-flank	CAGACGTACA	0.8789	0.8217	0.8868	0.9228	0.7230	0.8398
ACGT-flank	CAGACGTACC	0.8148	0.6407	0.5288	0.7161	0.6804	0.7910
ACGT-flank	CAGACGTACG	0.8223	0.8136	0.8945	0.8545	0.8868	0.6786
ACGT-flank	CAGACGTACT	0.8169	0.9870	0.8763	0.6803	0.7031	0.7796
ACGT-flank	CAGACGTAGA	0.5488	0.6753	0.9391	0.5521	0.6901	0.7029
ACGT-flank	CAGACGTAGC	0.8380	1.0234	0.7993	0.4505	0.8331	0.7936
ACGT-flank	CAGACGTAGG	1.1833	0.9100	0.9347	1.0365	1.0791	1.0378
ACGT-flank	CAGACGTAGT	0.9739	0.9217	0.8033	0.8061	0.7270	0.8178
ACGT-flank	CAGACGTATC	0.6551	0.7311	0.6703	0.5056	0.6680	0.7269
ACGT-flank	CAGACGTATG	0.9373	1.0597	0.9446	0.7935	0.9112	0.8565
ACGT-flank	CAGACGTCAC	0.7426	0.9665	0.5750	0.5475	0.6273	0.6869
ACGT-flank	CAGACGTCAT	0.7888	0.6374	1.0682	1.1514	0.7251	0.7888
ACGT-flank	CAGACGTCCG	0.7785	0.8039	0.9279	1.0212	0.8099	0.8148
ACGT-flank	CAGACGTCCT	1.1535	0.9856	1.0671	1.1591	0.9919	1.1582
ACGT-flank	CAGACGTCGC	1.3816	1.5254	0.8488	0.5244	0.9763	0.9406
ACGT-flank	CAGACGTCGG	0.9876	1.2479	0.7723	1.0228	0.8988	0.9032
ACGT-flank	CAGACGTCGT	0.9266	0.9161	0.9529	0.8366	1.0182	1.0776
ACGT-flank	CAGACGTCTA	1.6189	1.1909	0.8428	0.8221	1.2523	1.2531
ACGT-flank	CAGACGTCTC	0.9845	0.9375	1.1215	0.8575	1.0550	1.2209
ACGT-flank	CAGACGTCTG	0.8942	1.1148	1.1424	0.9236	0.9299	0.8386
ACGT-flank	CAGACGTGAG	1.0875	0.9689	1.0131	0.9355	0.8864	1.0832
ACGT-flank	CAGACGTGAT	0.8478	1.0553	1.1765	0.9711	0.9154	1.0336
ACGT-flank	CAGACGTGCA	0.8472	1.0096	0.7292	0.9750	0.8503	0.7811
ACGT-flank	CAGACGTGCG	0.4544	0.4733	0.4590	0.5216	0.8392	0.7812
ACGT-flank	CAGACGTGCT	0.9070	1.4127	0.9071	1.0117	0.9491	1.0023
ACGT-flank	CAGACGTGGA	0.9932	1.4025	1.3333	0.8486	1.1139	1.1215
ACGT-flank	CAGACGTGGC	1.1056	0.9682	1.0089	0.7029	1.1285	0.8963
ACGT-flank	CAGACGTGGG	1.2622	1.4014	1.2415	1.3965	1.2974	1.1972
ACGT-flank	CAGACGTGGT	1.1050	0.9169	0.8879	0.9933	0.9362	0.9304
ACGT-flank	CAGACGTGTA	1.1921	1.2128	1.1729	1.1472	1.2119	1.0615
ACGT-flank	CAGACGTGTC	1.0457	1.1623	0.8913	0.8317	0.8675	0.9977
ACGT-flank	CAGACGTGTG	1.1497	1.0685	1.2261	1.2193	1.2682	1.2450
ACGT-flank	CAGACGTGTT	1.3960	1.1143	1.2181	1.0878	1.5016	1.2720
ACGT-flank	CAGACGTTAG	0.7204	0.8370	0.9138	0.7987	0.7882	0.7904
ACGT-flank	CAGACGTTCA	0.7147	0.3891	0.9063	0.9939	0.9315	0.7841
ACGT-flank	CAGACGTTCC	0.8800	0.7064	0.7322	0.8239	0.8804	0.8066
ACGT-flank	CAGACGTTCG	1.0338	0.8098	0.9855	0.8999	0.8764	1.1205
ACGT-flank	CAGACGTTCT	1.1028	1.1299	1.0711	1.1048	1.0181	1.0089
ACGT-flank	CAGACGTTGA	0.9204	1.1773	0.6417	0.7360	0.6912	0.9412
ACGT-flank	CAGACGTTGC	1.0704	1.5421	1.3203	1.1134	1.2792	1.1112
ACGT-flank	CAGACGTTGG	1.1315	1.0806	1.1635	0.8416	1.0668	1.0713

ACGT-flank	CAGACGTTGT	0.8603	0.9886	0.8469	0.7526	0.7206	0.8663
ACGT-flank	CAGACGTTTG	1.0790	0.6647	1.1177	1.0924	0.9671	0.7977
ACGT-flank	CATACGTACG	1.0878	0.8846	0.8433	0.8550	0.9700	0.7950
ACGT-flank	CATACGTAGC	0.9371	0.9022	0.9213	0.7221	0.4387	0.6088
ACGT-flank	CATACGTAGG	1.2709	1.1638	1.1917	1.2452	1.1304	1.2955
ACGT-flank	CATACGTAGT	0.7754	0.5826	0.8269	0.5589	0.6737	0.8221
ACGT-flank	CATACGTCCC	0.8634	1.0857	0.7883	0.8136	0.8834	0.8800
ACGT-flank	CATACGTCCG	0.9616	1.0569	0.9268	1.0340	0.9654	0.9839
ACGT-flank	CATACGTCGA	2.5185	1.9406	1.8033	1.3913	1.5696	1.9291
ACGT-flank	CATACGTCGC	0.7236	0.8901	0.8346	0.5582	0.7222	0.7875
ACGT-flank	CATACGTCGG	0.5916	0.7213	0.5555	0.5892	0.6361	0.6485
ACGT-flank	CATACGTCTG	0.7017	0.8397	0.7478	0.8843	0.9819	0.8818
ACGT-flank	CATACGTGAA	0.8256	1.0736	1.2594	1.1465	1.0004	1.0923
ACGT-flank	CATACGTGAC	1.1277	0.6906	1.1685	1.1323	0.9120	0.8213
ACGT-flank	CATACGTGAG	0.8435	0.9552	0.6669	0.8513	0.5928	0.5299
ACGT-flank	CATACGTGCA	0.9527	0.7346	0.7200	0.9553	0.9121	0.8978
ACGT-flank	CATACGTGCC	0.8824	0.6332	1.0724	1.0698	0.7616	0.7291
ACGT-flank	CATACGTGCG	0.9040	0.8466	0.8010	0.8187	0.8798	0.9060
ACGT-flank	CATACGTGGA	1.0198	1.0231	1.2325	1.0750	1.1064	0.9719
ACGT-flank	CATACGTGGC	1.1501	1.0089	1.1836	1.2093	1.1595	1.0012
ACGT-flank	CATACGTGGG	1.4666	1.3678	1.4413	1.3978	1.4176	1.3922
ACGT-flank	CATACGTGGT	0.7326	0.7713	0.6393	0.8937	0.6885	0.6789
ACGT-flank	CATACGTGTC	1.1111	1.6949	1.2417	1.3982	1.4996	1.6967
ACGT-flank	CATACGTGTG	1.0985	1.1156	1.5097	1.0862	1.1471	1.1119
ACGT-flank	CATACGTTGG	1.3159	1.0773	1.0678	1.2373	1.1674	1.1611
ACGT-flank	CCAACGTACG	1.1644	0.8787	1.3927	1.0423	0.9160	1.1118
ACGT-flank	CCAACGTACT	1.1363	1.2477	1.1992	1.0819	1.0576	1.0435
ACGT-flank	CCAACGTAGA	1.1513	1.0329	1.1639	1.2067	0.9975	1.1051
ACGT-flank	CCAACGTAGG	1.2624	1.2093	1.2977	1.1779	1.2606	1.1759
ACGT-flank	CCAACGTATG	1.0740	1.0810	0.9398	1.0777	1.2272	1.1331
ACGT-flank	CCAACGTCGG	0.9455	0.9739	1.0514	1.3173	1.1916	1.0970
ACGT-flank	CCAACGTCTG	0.8552	0.8103	0.9345	0.9398	0.9131	1.0275
ACGT-flank	CCAACGTGAA	0.8862	0.9083	0.8543	0.7816	0.9267	1.0130
ACGT-flank	CCAACGTGAG	1.1124	0.8228	0.8146	0.9506	0.7263	0.8436
ACGT-flank	CCAACGTGAT	1.1004	0.8388	0.7575	0.8997	1.0201	0.8435
ACGT-flank	CCAACGTGCA	0.9386	1.0022	1.0637	0.8942	1.1621	0.9683
ACGT-flank	CCAACGTGGA	0.9897	1.1407	0.9591	1.0662	1.0479	1.0268
ACGT-flank	CCAACGTGGC	0.7159	1.0284	0.8439	0.8189	0.8338	0.9369
ACGT-flank	CCAACGTGGG	1.2594	1.1857	1.1508	1.2784	1.2644	1.3644
ACGT-flank	CCAACGTGGT	0.9152	0.9693	0.9536	0.8471	0.9620	1.0436
ACGT-flank	CCAACGTGTA	0.7146	0.7780	1.0974	0.7144	0.6271	0.6901
ACGT-flank	CCAACGTGTC	1.3306	1.8041	1.2411	1.2986	1.1986	1.6625
ACGT-flank	CCAACGTGTG	1.0316	1.0702	1.2143	1.1104	1.1329	1.0744
ACGT-flank	CCAACGTGTT	1.3091	1.3908	0.9736	0.9815	1.2540	0.9749

ACGT-flank	CCAACGTTAC	0.7421	0.9585	0.7293	0.7425	0.7132	0.7374
ACGT-flank	CCAACGTTAG	0.8860	0.7825	0.8566	0.9833	1.0902	0.9122
ACGT-flank	CCAACGTTCG	1.2197	0.7406	1.3515	1.0277	1.0054	0.9875
ACGT-flank	CCAACGTTGA	1.2332	0.9964	1.1600	1.2019	1.3527	1.2265
ACGT-flank	CCAACGTTGC	0.9767	0.9834	1.0870	0.8493	0.6708	0.6886
ACGT-flank	CCAACGTTGG	0.8832	1.0289	0.9324	0.7770	0.8500	0.8777
ACGT-flank	CCAACGTTGT	0.7497	0.6764	0.8382	0.7931	0.7279	0.6909
ACGT-flank	CCCACGTACC	0.8981	0.9055	0.9052	0.9158	0.8898	0.9066
ACGT-flank	CCCACGTACG	0.8128	0.7573	0.5988	0.7427	0.7610	0.9275
ACGT-flank	CCCACGTACT	0.8685	0.7879	0.8462	0.8998	0.9706	0.8205
ACGT-flank	CCCACGTAGG	1.0189	1.1965	0.9664	1.1833	1.0955	0.9339
ACGT-flank	CCCACGTAGT	0.8192	0.7719	0.8564	0.6613	0.7615	0.7361
ACGT-flank	CCCACGTATC	1.1766	1.3128	1.3003	1.1564	1.2235	1.3767
ACGT-flank	CCCACGTATG	0.8720	1.1342	0.9613	1.0058	0.9342	1.0785
ACGT-flank	CCCACGTCCC	1.3980	0.8435	1.1567	0.9880	1.0179	1.0566
ACGT-flank	CCCACGTCCT	0.8807	1.0889	1.1128	1.0405	0.9139	0.9345
ACGT-flank	CCCACGTCGC	0.9156	0.8225	0.8331	0.6666	0.7243	0.9229
ACGT-flank	CCCACGTCGG	0.7909	1.0404	0.7368	0.9592	0.8840	0.8439
ACGT-flank	CCCACGTCGT	0.7777	0.9025	0.8697	0.8527	0.8714	0.8006
ACGT-flank	CCCACGTCTA	1.0023	0.9631	1.0127	0.7539	0.9054	0.9893
ACGT-flank	CCCACGTCTC	0.5303	0.4457	0.4213	0.4583	0.4741	0.5167
ACGT-flank	CCCACGTCTG	0.9040	0.9890	1.0889	0.7659	0.8999	0.8464
ACGT-flank	CCCACGTCTT	1.2488	1.6844	1.2648	1.1639	1.4087	1.4280
ACGT-flank	CCCACGTGAG	0.9448	1.1090	0.7367	0.5772	0.7171	0.6375
ACGT-flank	CCCACGTGAT	0.7755	0.9541	0.9046	0.8804	0.7270	0.9412
ACGT-flank	CCCACGTGCG	1.0531	1.0555	1.1281	1.4113	1.2368	1.0598
ACGT-flank	CCCACGTGGG	1.3461	1.5264	1.0029	1.6318	1.3748	1.3254
ACGT-flank	CCCACGTGGT	0.9197	0.8429	0.8536	0.8034	0.8540	0.8471
ACGT-flank	CCCACGTGTC	1.3346	1.5098	1.6130	1.6539	1.6741	1.4431
ACGT-flank	CCCACGTGTG	0.7302	0.8143	1.0382	0.8135	1.0160	0.9219
ACGT-flank	CCCACGTTCA	1.0880	0.8869	0.7467	1.2105	0.6175	0.7470
ACGT-flank	CCCACGTTCC	1.0018	0.9036	0.9655	0.9220	0.9128	0.9215
ACGT-flank	CCCACGTTCG	0.9832	0.8807	0.8417	0.9308	0.8683	0.9124
ACGT-flank	CCCACGTTGA	0.6879	0.8520	0.8043	0.8751	0.8198	0.8368
ACGT-flank	CCCACGTTGC	0.8966	0.5741	0.7594	0.6010	0.7074	0.7557
ACGT-flank	CCCACGTTGG	3.0035	2.8430	2.9723	3.1196	2.9642	2.9413
ACGT-flank	CCCACGTTGT	0.7409	0.5803	0.6469	0.5116	0.7251	0.7772
ACGT-flank	CCCACGTTTC	0.7240	0.7883	1.0884	0.8860	1.0479	0.9223
ACGT-flank	CCCACGTTTG	0.9904	0.8872	0.8493	1.2740	0.9952	1.0279
ACGT-flank	CCGACGTAAA	1.1922	1.0316	0.8768	0.7448	0.9529	1.2018
ACGT-flank	CCGACGTAAC	0.9763	0.9760	1.2769	0.8270	0.9354	0.8899
ACGT-flank	CCGACGTACA	1.1532	1.4655	1.3779	1.3906	1.3666	1.5395
ACGT-flank	CCGACGTACC	0.6814	0.9221	0.7520	0.7220	0.8432	0.8711
ACGT-flank	CCGACGTACT	0.8464	0.7869	1.0018	1.1002	1.0010	0.8633

ACGT-flank	CCGACGTAGA	0.9971	0.7395	0.9585	0.7951	0.7845	0.6177
ACGT-flank	CCGACGTAGG	1.1860	1.1005	1.0348	1.0245	0.9529	1.0152
ACGT-flank	CCGACGTAGT	0.6413	0.5843	0.7321	0.7750	0.8771	0.6951
ACGT-flank	CCGACGTATA	0.5045	0.4758	0.4313	0.4959	0.3752	0.2533
ACGT-flank	CCGACGTATC	1.2802	1.0989	1.2726	1.0182	1.3454	1.1296
ACGT-flank	CCGACGTATG	1.0493	1.0773	1.0634	1.1716	1.0190	1.0155
ACGT-flank	CCGACGTCAC	0.6897	0.5076	0.5894	0.7939	0.4180	0.9714
ACGT-flank	CCGACGTCAG	0.9548	0.9204	1.0201	0.7870	0.8717	0.7975
ACGT-flank	CCGACGTCAT	0.8848	0.5838	0.5050	0.6999	0.8202	0.6986
ACGT-flank	CCGACGTCCT	0.8307	0.6704	0.8683	0.7180	0.8218	0.7628
ACGT-flank	CCGACGTCGG	0.9034	0.7559	1.0125	0.7961	0.8818	0.9121
ACGT-flank	CCGACGTCGT	0.8154	0.7718	0.8286	0.6195	0.7825	0.7585
ACGT-flank	CCGACGTCTA	0.9233	0.9326	1.0530	0.6689	0.8824	0.8413
ACGT-flank	CCGACGTCTC	1.0423	0.9468	0.9409	0.9732	0.9669	0.9148
ACGT-flank	CCGACGTCTG	0.7633	0.8467	0.9227	0.8574	0.8238	0.9584
ACGT-flank	CCGACGTCTT	1.1603	1.3740	1.4323	1.4981	1.5638	1.4037
ACGT-flank	CCGACGTGAC	0.8454	1.0774	0.6973	1.0787	0.8090	0.9555
ACGT-flank	CCGACGTGAG	1.0552	0.7885	0.8554	0.8508	0.8811	0.7175
ACGT-flank	CCGACGTGAT	0.8978	0.5747	0.7111	0.7918	0.6081	0.8396
ACGT-flank	CCGACGTGCT	0.9208	0.7339	0.7682	0.7707	0.8134	0.9296
ACGT-flank	CCGACGTGGC	0.9237	0.7088	0.9296	1.0748	0.7745	0.8803
ACGT-flank	CCGACGTGGG	1.1603	1.4139	1.2340	1.2129	1.1922	1.2251
ACGT-flank	CCGACGTGGT	0.8886	0.9188	1.0318	0.9757	1.0122	0.9400
ACGT-flank	CCGACGTGTA	1.2477	1.2046	1.2121	1.3820	1.0149	1.1318
ACGT-flank	CCGACGTGTC	0.7054	0.7667	0.5132	0.8307	0.9048	0.7810
ACGT-flank	CCGACGTGTG	1.1674	1.5202	1.1937	1.2913	1.2322	1.1446
ACGT-flank	CCGACGTGTT	0.7235	1.0710	0.8780	0.9485	0.9101	0.9629
ACGT-flank	CCGACGTTAA	0.7269	1.1402	0.7312	0.7878	0.6805	0.7465
ACGT-flank	CCGACGTTAG	0.9023	0.8715	0.9255	0.9002	1.0423	1.2108
ACGT-flank	CCGACGTTCA	0.9186	1.0581	0.7051	0.8574	0.8633	0.9014
ACGT-flank	CCGACGTTCC	0.9066	1.0331	0.8888	1.0614	0.9291	0.9457
ACGT-flank	CCGACGTTCG	1.1484	1.0217	0.9833	0.6223	0.9256	0.8770
ACGT-flank	CCGACGTTCT	0.7565	0.7878	0.9981	1.1487	1.0439	0.9930
ACGT-flank	CCGACGTTGA	1.0076	0.8652	1.1955	0.9124	0.8986	0.8465
ACGT-flank	CCGACGTTGC	1.3072	0.9614	0.9221	1.1659	1.0608	0.9901
ACGT-flank	CCGACGTTGG	0.8944	0.8279	0.8302	0.8704	0.8547	0.8522
ACGT-flank	CCGACGTTGT	1.1192	0.9854	1.0099	1.1049	1.0096	1.0591
ACGT-flank	CCGACGTTTC	1.0362	1.0761	0.8558	0.8163	0.9647	0.7435
ACGT-flank	CCGACGTTTG	0.9809	0.8265	1.0759	0.9881	0.9298	1.0231
ACGT-flank	CCTACGTAAG	1.1540	0.8646	0.9420	1.0281	0.9305	0.9208
ACGT-flank	CCTACGTACC	0.8218	0.6569	0.7678	0.6471	0.8268	0.7838
ACGT-flank	CCTACGTACG	0.9301	1.0124	0.8801	0.9440	0.9306	0.8210
ACGT-flank	CCTACGTACT	0.9498	1.1924	0.8845	0.7331	0.8899	1.1264
ACGT-flank	CCTACGTAGG	0.9876	0.9230	0.9557	0.9976	1.0167	0.9106

ACGT-flank	CCTACGTAGT	1.0437	0.7375	0.8267	1.1607	0.9183	0.8908
ACGT-flank	CCTACGTATG	0.8924	1.0517	1.1498	0.9151	0.8598	0.9708
ACGT-flank	CCTACGTCAC	0.9609	1.0911	0.9250	1.0226	0.9839	0.8944
ACGT-flank	CCTACGTCAG	1.3127	0.9657	1.2061	0.9870	1.2980	1.1255
ACGT-flank	CCTACGTCCA	0.8962	1.0827	0.9933	0.8692	1.1691	0.7580
ACGT-flank	CCTACGTCCC	0.9182	0.9517	0.7424	0.8844	0.8953	0.8930
ACGT-flank	CCTACGTCCG	0.8241	1.2760	1.2192	1.2509	1.0562	1.0961
ACGT-flank	CCTACGTCCT	0.8878	1.0128	0.9780	0.7872	0.9371	0.9330
ACGT-flank	CCTACGTCGC	0.5559	0.5938	0.4381	0.4100	0.5858	0.5870
ACGT-flank	CCTACGTCGG	0.8023	0.7435	0.8341	0.7421	0.8575	0.7070
ACGT-flank	CCTACGTCGT	0.8840	0.9051	0.8960	0.7859	0.9316	0.8569
ACGT-flank	CCTACGTCTA	0.9600	1.0451	1.0622	0.5950	1.0204	0.8258
ACGT-flank	CCTACGTCTC	0.9441	1.0527	0.9549	0.9069	0.9582	0.9962
ACGT-flank	CCTACGTCTG	1.0178	0.8619	0.8971	0.7831	0.8041	0.9895
ACGT-flank	CCTACGTGAA	0.7711	1.2454	0.9423	0.9078	0.6377	0.7709
ACGT-flank	CCTACGTGAC	0.7325	1.0525	0.8584	1.1548	0.9300	0.9681
ACGT-flank	CCTACGTGAG	0.8198	0.7954	0.8073	0.7850	0.8249	0.8906
ACGT-flank	CCTACGTGAT	0.9212	1.4927	1.0700	1.0559	1.0633	0.8896
ACGT-flank	CCTACGTGCC	0.6527	0.8673	0.8749	0.7383	0.7759	0.7232
ACGT-flank	CCTACGTGCG	0.9062	0.8846	0.7772	0.8236	0.8827	0.9109
ACGT-flank	CCTACGTGCT	0.9865	1.4016	1.1721	0.6806	1.0500	0.9972
ACGT-flank	CCTACGTGGA	1.2720	1.1072	1.2400	1.3510	1.2697	1.2014
ACGT-flank	CCTACGTGGC	1.0009	1.2725	1.3458	1.2108	1.1696	1.2009
ACGT-flank	CCTACGTGGG	1.5684	1.1570	1.3349	1.5846	1.4017	1.4251
ACGT-flank	CCTACGTGGT	0.8015	0.6744	0.7350	0.9841	0.7591	0.8196
ACGT-flank	CCTACGTGTC	0.9703	1.0209	1.0048	1.1602	1.1455	1.1212
ACGT-flank	CCTACGTGTG	1.1195	1.2957	0.9893	1.0773	1.1628	1.0065
ACGT-flank	CCTACGTGTT	1.3358	1.4561	1.3537	1.3508	1.2363	1.4514
ACGT-flank	CCTACGTTCC	1.0721	0.9050	1.0066	0.8079	0.9789	0.9955
ACGT-flank	CCTACGTTCG	0.7772	0.6851	0.6787	0.8286	0.7570	0.6192
ACGT-flank	CCTACGTTGA	0.9153	1.0495	1.0965	0.6862	1.0107	0.7915
ACGT-flank	CCTACGTTGC	0.8745	1.0732	0.7942	0.9065	0.9453	0.8051
ACGT-flank	CCTACGTTGG	0.9063	0.8407	1.0079	0.9427	0.7946	0.8869
ACGT-flank	CCTACGTTGT	1.4062	0.8419	1.2329	0.8902	1.3825	1.0815
ACGT-flank	CGAACGTAAC	0.6104	1.1436	0.6595	1.1170	0.9935	1.0361
ACGT-flank	CGAACGTAAG	0.8960	0.7206	0.7580	0.8837	0.7806	0.7926
ACGT-flank	CGAACGTACA	1.0036	0.8394	0.8492	0.7178	0.9272	0.7369
ACGT-flank	CGAACGTACC	0.7970	0.8144	0.6101	0.7352	0.9701	0.8404
ACGT-flank	CGAACGTACG	0.8149	0.7550	0.8888	0.7677	0.8172	0.4898
ACGT-flank	CGAACGTACT	1.4775	1.2306	0.9947	1.0590	0.8936	1.1976
ACGT-flank	CGAACGTAGA	0.6538	0.7794	0.6750	0.6505	0.6723	0.6289
ACGT-flank	CGAACGTAGC	0.8489	0.5617	0.8564	0.8011	0.8407	0.9341
ACGT-flank	CGAACGTAGG	1.1161	0.8771	1.0596	1.0199	1.0991	1.0607
ACGT-flank	CGAACGTAGT	0.7369	0.8259	0.8934	0.8370	0.8400	0.9220

ACGT-flank	CGAACGTATC	0.7161	0.9510	0.8777	0.7591	0.8478	0.8797
ACGT-flank	CGAACGTATG	1.0122	1.1468	1.0992	0.8466	0.9702	0.9976
ACGT-flank	CGAACGTCAA	0.6609	0.8325	0.7340	0.9052	0.5907	0.5868
ACGT-flank	CGAACGTCAC	0.9520	1.0636	0.8774	0.9060	0.8707	0.9311
ACGT-flank	CGAACGTCAG	0.8300	0.7469	0.7262	0.9677	0.8433	0.8464
ACGT-flank	CGAACGTCAT	0.6352	0.4766	0.6378	0.6936	0.5036	0.5725
ACGT-flank	CGAACGTCCA	1.0716	1.0178	1.0836	1.1127	1.0209	0.9236
ACGT-flank	CGAACGTCCG	1.2381	0.7576	0.7971	0.7851	1.0745	0.9229
ACGT-flank	CGAACGTCCT	0.5507	0.5348	0.6329	0.8733	0.5958	0.6563
ACGT-flank	CGAACGTCGA	0.8748	0.6378	0.8197	0.9113	0.8208	0.9096
ACGT-flank	CGAACGTCGC	0.7412	0.6793	0.9822	0.7976	0.7519	0.7288
ACGT-flank	CGAACGTCGG	1.0822	1.0876	0.9865	0.9306	1.0235	0.9379
ACGT-flank	CGAACGTCGT	0.9190	0.7515	0.9240	0.8520	0.8772	0.8373
ACGT-flank	CGAACGTCTA	0.9238	0.9084	0.7684	0.8159	0.7679	0.7315
ACGT-flank	CGAACGTCTC	0.6933	0.4795	0.5827	0.6382	0.7526	0.6827
ACGT-flank	CGAACGTCTG	0.7273	0.7719	0.9229	0.8478	0.8816	0.8874
ACGT-flank	CGAACGTGAA	0.9325	0.8754	0.8819	0.8704	0.8649	0.9566
ACGT-flank	CGAACGTGAC	0.6829	0.9889	0.8087	1.2453	1.0927	1.1269
ACGT-flank	CGAACGTGAG	0.9653	0.8069	0.8019	0.8564	0.7433	0.8368
ACGT-flank	CGAACGTGAT	0.8432	0.9190	0.8972	0.8035	0.7285	0.8220
ACGT-flank	CGAACGTGCA	1.1057	1.0525	0.9206	0.9050	1.0183	0.9583
ACGT-flank	CGAACGTGCC	0.6126	0.8413	0.8305	0.6187	0.7156	0.8955
ACGT-flank	CGAACGTGCG	0.7010	1.0151	0.7875	0.7272	0.7061	0.5717
ACGT-flank	CGAACGTGCT	0.8659	0.8686	0.8531	0.8794	0.7825	0.8217
ACGT-flank	CGAACGTGGA	0.8867	0.9125	0.8313	0.9040	0.8390	0.8850
ACGT-flank	CGAACGTGGC	0.5556	0.5719	0.5952	0.9301	0.5618	0.6886
ACGT-flank	CGAACGTGGG	1.6637	1.6055	1.6838	1.6897	1.5687	1.4199
ACGT-flank	CGAACGTGGT	0.7352	0.7898	0.8028	0.6695	0.7724	0.7801
ACGT-flank	CGAACGTGTA	1.2813	1.2182	1.4129	1.0309	1.2199	1.2826
ACGT-flank	CGAACGTGTC	0.9973	0.9993	1.0848	0.8981	0.8898	1.0411
ACGT-flank	CGAACGTGTG	1.2321	1.1689	1.1995	1.1684	1.1673	1.1127
ACGT-flank	CGAACGTGTT	1.2292	1.0772	1.1433	1.2663	1.3793	1.3670
ACGT-flank	CGAACGTTAG	0.8734	0.8670	0.9254	0.7900	0.8797	0.9182
ACGT-flank	CGAACGTTCA	1.1318	0.6108	0.7096	0.7811	0.7154	0.5189
ACGT-flank	CGAACGTTCC	1.2873	1.2290	1.3378	1.1163	1.3086	1.2386
ACGT-flank	CGAACGTTCG	0.8631	1.0531	1.0890	0.7571	0.8659	0.8940
ACGT-flank	CGAACGTTCT	0.9441	0.6767	0.8657	0.6390	1.2032	1.3594
ACGT-flank	CGAACGTTGA	0.8678	0.9101	0.7789	0.8431	0.7350	0.7923
ACGT-flank	CGAACGTTGC	0.9700	0.9902	0.8888	0.7745	0.9735	1.0091
ACGT-flank	CGAACGTTGG	0.9748	1.0273	0.9514	0.9281	1.0956	0.9830
ACGT-flank	CGAACGTTGT	0.8750	0.7426	0.8146	0.9006	0.8731	0.9384
ACGT-flank	CGAACGTTTG	0.9132	0.7555	0.8380	0.8888	0.7431	0.8295
ACGT-flank	CGCACGTAAC	0.5092	0.6409	0.5683	0.3045	0.6643	0.5943
ACGT-flank	CGCACGTAAG	0.4736	0.5484	0.7391	0.6643	0.7510	0.5741

ACGT-flank	CGCACGTAAT	0.8238	0.7286	0.5867	0.7679	0.7460	0.7930
ACGT-flank	CGCACGTACA	1.0502	0.7227	0.7528	0.7324	0.9184	0.8157
ACGT-flank	CGCACGTACC	1.0379	1.1246	1.2595	0.9621	1.3112	1.0459
ACGT-flank	CGCACGTACG	1.0638	1.2749	0.7820	1.0422	1.0576	0.9496
ACGT-flank	CGCACGTACT	1.0632	0.9231	0.9189	0.7301	0.9320	0.9386
ACGT-flank	CGCACGTAGA	1.0728	1.1254	1.1768	0.8896	0.8303	0.8176
ACGT-flank	CGCACGTAGC	0.8166	0.9570	0.7508	0.7919	0.7793	0.7870
ACGT-flank	CGCACGTAGG	1.1361	1.0926	0.9743	0.9944	1.1404	1.2167
ACGT-flank	CGCACGTAGT	0.7473	0.7363	0.7727	0.6564	0.8046	0.8713
ACGT-flank	CGCACGTATG	0.9369	0.8645	0.8684	0.9602	0.8223	1.0686
ACGT-flank	CGCACGTCAA	1.1008	0.7126	0.7231	1.2117	0.8900	1.0097
ACGT-flank	CGCACGTCAG	0.8497	0.8998	0.8706	0.7780	0.7383	0.8216
ACGT-flank	CGCACGTCAT	1.3161	1.1073	1.0327	1.2647	1.2339	1.1958
ACGT-flank	CGCACGTCCA	0.7529	1.1705	1.2858	0.8371	1.0420	0.8969
ACGT-flank	CGCACGTCCC	0.6877	0.6542	0.6581	0.6996	0.7471	0.8161
ACGT-flank	CGCACGTCCG	0.9374	0.7474	0.9791	0.9838	1.3328	0.9793
ACGT-flank	CGCACGTCCT	0.7273	0.8408	0.9229	0.4718	0.9112	0.7821
ACGT-flank	CGCACGTCGA	0.8832	0.7483	0.7825	0.5930	0.9577	0.9747
ACGT-flank	CGCACGTCGC	0.9952	0.8069	0.8955	0.7268	0.8732	0.8162
ACGT-flank	CGCACGTCGG	1.2161	1.1333	1.1532	0.9062	1.2114	1.1215
ACGT-flank	CGCACGTCGT	0.7358	0.8092	0.7633	0.6053	0.5902	0.6989
ACGT-flank	CGCACGTCTA	0.6305	0.8105	1.0239	0.8795	0.8882	0.8863
ACGT-flank	CGCACGTCTC	0.8898	1.1349	0.9972	0.9091	1.0581	1.0830
ACGT-flank	CGCACGTCTG	0.8632	0.7061	0.8177	1.0618	0.9255	0.8638
ACGT-flank	CGCACGTCTT	0.8779	1.0548	0.8281	1.0060	1.2316	1.2031
ACGT-flank	CGCACGTGAC	0.8902	0.6230	0.7601	0.6371	0.6050	0.7215
ACGT-flank	CGCACGTGAG	0.9677	0.9276	1.0678	1.0516	1.0847	1.0750
ACGT-flank	CGCACGTGAT	0.9208	0.7393	0.9108	0.9632	0.8812	0.8857
ACGT-flank	CGCACGTGCA	0.8930	1.1039	0.7393	0.8353	0.8828	0.8268
ACGT-flank	CGCACGTGCC	0.9332	1.5573	1.1338	1.0273	1.0620	0.9157
ACGT-flank	CGCACGTGCG	0.9019	1.2736	1.2381	1.5043	1.5137	1.2813
ACGT-flank	CGCACGTGCT	0.9484	0.8398	0.8894	0.9583	0.8454	1.1691
ACGT-flank	CGCACGTGGA	1.1594	1.0912	1.1520	1.0814	1.1822	1.1843
ACGT-flank	CGCACGTGGG	1.1640	1.3877	1.2977	1.0164	1.1543	1.2629
ACGT-flank	CGCACGTGGT	1.1010	1.1854	1.0669	0.9032	1.1011	1.0791
ACGT-flank	CGCACGTGTA	1.2545	1.1826	1.4478	1.1260	1.4027	1.1290
ACGT-flank	CGCACGTGTC	1.2280	1.1183	1.2212	1.2878	1.3238	1.3423
ACGT-flank	CGCACGTGTG	0.9593	1.0920	1.3046	1.0206	1.1654	1.1745
ACGT-flank	CGCACGTGTT	1.1749	1.2355	1.2279	1.2516	1.1446	1.2759
ACGT-flank	CGCACGTTAC	1.1330	1.0158	1.1538	1.0219	1.2845	1.0535
ACGT-flank	CGCACGTTAG	0.9152	0.6636	0.8083	0.8895	0.9446	0.9558
ACGT-flank	CGCACGTTCA	0.9462	0.6852	0.8234	0.8412	0.7884	1.0535
ACGT-flank	CGCACGTTCC	0.9066	1.0886	1.0343	1.1079	1.0096	0.9412
ACGT-flank	CGCACGTTCG	1.0245	0.9400	0.8698	0.9720	0.9055	0.9789

ACGT-flank	CGCACGTTCT	0.7388	0.8812	0.7836	1.0035	0.4237	0.6797
ACGT-flank	CGCACGTTGA	0.9581	1.0229	0.8815	0.8476	0.8991	0.9971
ACGT-flank	CGCACGTTGC	0.7590	0.5570	0.6435	0.8095	0.7044	0.9368
ACGT-flank	CGCACGTTGG	0.9614	0.9339	0.8843	0.8884	0.8787	0.8742
ACGT-flank	CGCACGTTGT	1.2801	1.3455	1.5438	1.2408	1.2817	1.2676
ACGT-flank	CGCACGTTTG	0.7953	0.9539	0.8962	0.6342	0.7197	0.6654
ACGT-flank	CGGACGTAAA	0.8690	0.9348	1.0822	0.9478	0.8567	0.8516
ACGT-flank	CGGACGTAAC	0.6986	0.7603	0.8515	0.9299	0.8145	0.9203
ACGT-flank	CGGACGTAAG	0.7831	0.6431	0.8509	0.8132	0.8178	0.8543
ACGT-flank	CGGACGTAAT	0.7769	0.7663	0.7929	0.7894	0.7924	0.7351
ACGT-flank	CGGACGTACA	0.9039	0.9137	0.6209	0.8733	0.8928	0.9507
ACGT-flank	CGGACGTACC	0.7780	0.9044	0.8804	0.9046	0.8610	0.9521
ACGT-flank	CGGACGTACG	0.7881	0.8136	0.9754	0.8341	0.9377	0.9299
ACGT-flank	CGGACGTACT	0.9391	0.9151	0.9284	0.8307	0.9442	0.9270
ACGT-flank	CGGACGTAGA	0.7793	0.7086	0.7435	0.7464	0.8365	0.8477
ACGT-flank	CGGACGTAGC	0.6283	0.7004	0.7588	0.8092	0.8141	0.7772
ACGT-flank	CGGACGTAGG	1.0842	0.8698	1.2780	1.2058	1.2327	1.3008
ACGT-flank	CGGACGTAGT	0.8441	0.8783	0.8420	0.8743	0.8728	0.8363
ACGT-flank	CGGACGTATA	0.9473	0.9052	0.8751	0.8451	0.7353	0.8294
ACGT-flank	CGGACGTATC	0.7770	0.7669	0.7413	0.7440	0.7242	0.8192
ACGT-flank	CGGACGTATG	0.9637	1.0337	0.9975	0.9673	0.9037	0.9903
ACGT-flank	CGGACGTATT	1.0446	1.1954	1.2935	1.0819	1.1216	1.2779
ACGT-flank	CGGACGTCAA	0.6617	0.7196	0.5954	0.6163	0.6879	0.6598
ACGT-flank	CGGACGTCAC	1.0762	0.9702	0.8529	0.9522	1.0486	1.0662
ACGT-flank	CGGACGTCAG	1.0833	0.9144	0.9695	0.9917	0.8518	0.7677
ACGT-flank	CGGACGTCAT	0.8130	0.5779	0.8372	0.6474	0.7105	0.7662
ACGT-flank	CGGACGTCCA	0.7281	0.5376	0.8547	0.8107	0.8158	0.7678
ACGT-flank	CGGACGTCCC	0.9190	1.1192	0.7793	0.9286	0.8772	0.9366
ACGT-flank	CGGACGTCCG	0.9267	0.6050	0.8738	0.6674	0.7067	0.7209
ACGT-flank	CGGACGTCCT	0.9477	0.7605	0.9168	0.8322	0.8774	0.9579
ACGT-flank	CGGACGTCGA	0.7777	1.0400	0.8692	1.0957	0.9525	0.9775
ACGT-flank	CGGACGTCGC	0.7683	0.7699	0.7956	0.8945	0.7138	0.7284
ACGT-flank	CGGACGTCGG	0.7674	0.8756	0.8672	0.7146	0.8489	0.8753
ACGT-flank	CGGACGTCGT	0.7577	0.7294	0.7124	0.7469	0.7101	0.6978
ACGT-flank	CGGACGTCTA	0.9118	0.9754	0.8899	0.7506	0.7437	0.9510
ACGT-flank	CGGACGTCTC	0.7621	0.7966	0.8235	0.6963	0.7609	0.7763
ACGT-flank	CGGACGTCTG	1.0802	0.9714	1.0497	0.9699	0.9179	0.9747
ACGT-flank	CGGACGTCTT	1.4772	1.3388	1.5562	1.4225	1.4093	1.3213
ACGT-flank	CGGACGTGAA	0.8697	0.8743	0.9618	0.7550	0.7132	0.8398
ACGT-flank	CGGACGTGAC	0.7865	0.8505	0.8835	0.8750	0.7959	0.7187
ACGT-flank	CGGACGTGAG	0.9668	0.7582	0.7787	0.9613	0.8414	0.9432
ACGT-flank	CGGACGTGAT	1.0917	1.0569	0.9145	1.0466	1.0314	0.9516
ACGT-flank	CGGACGTGCC	0.8705	0.6825	0.6583	1.0439	0.9073	0.8336
ACGT-flank	CGGACGTGCG	0.9145	0.7897	0.8667	0.7216	0.8263	0.8769

ACGT-flank	CGGACGTGCT	0.9000	0.9850	0.9501	0.9354	0.7933	0.8021
ACGT-flank	CGGACGTGGA	1.3330	1.2845	1.2041	1.2671	1.3564	1.3314
ACGT-flank	CGGACGTGGC	0.9409	0.7622	0.6311	0.9504	1.0370	0.8912
ACGT-flank	CGGACGTGGG	1.5746	1.4510	1.6449	1.6680	1.5631	1.5607
ACGT-flank	CGGACGTGGT	0.9651	0.9435	1.0236	0.8932	0.9187	0.9750
ACGT-flank	CGGACGTGTA	0.8076	0.8012	0.8718	0.7923	0.8591	0.8294
ACGT-flank	CGGACGTGTC	0.8972	1.0047	0.8113	1.0301	0.9794	0.8909
ACGT-flank	CGGACGTGTG	1.0910	1.0517	0.9874	1.0832	1.1190	1.1327
ACGT-flank	CGGACGTGTT	0.9639	0.9822	0.9280	1.2337	1.0221	1.0494
ACGT-flank	CGGACGTTAA	0.6956	0.7717	0.8923	0.8638	0.9026	0.7833
ACGT-flank	CGGACGTTAC	0.9976	0.6601	0.7013	0.9385	0.7969	0.7984
ACGT-flank	CGGACGTTAG	0.9186	0.8520	0.8711	0.8061	0.7725	0.8388
ACGT-flank	CGGACGTTAT	0.6861	1.0450	0.7687	0.6106	0.6915	0.7405
ACGT-flank	CGGACGTTCA	0.9533	1.1228	1.1765	1.0459	0.9814	1.0410
ACGT-flank	CGGACGTTCC	0.8359	1.1698	1.0287	1.0181	0.9029	0.8449
ACGT-flank	CGGACGTTCG	0.9049	0.8070	0.8122	0.8300	0.8181	0.8867
ACGT-flank	CGGACGTTCT	1.0656	1.1123	1.2165	0.9617	1.0005	1.0603
ACGT-flank	CGGACGTTGA	0.7279	0.6132	0.7587	0.7342	0.7069	0.7227
ACGT-flank	CGGACGTTGC	0.8969	0.9772	0.8266	0.8156	0.9275	1.0184
ACGT-flank	CGGACGTTGG	1.0304	1.0287	0.8950	1.0261	1.0295	0.9551
ACGT-flank	CGGACGTTGT	0.9335	0.8199	0.9516	0.9236	0.9031	0.8157
ACGT-flank	CGGACGTTTC	0.7678	0.9550	0.8508	0.8994	0.9431	0.8625
ACGT-flank	CGGACGTTTG	0.9583	0.9999	1.0664	0.8418	0.8547	0.8673
ACGT-flank	CGTACGTAAC	0.7670	0.8637	0.8354	0.7268	0.6992	0.7348
ACGT-flank	CGTACGTAAG	0.8356	0.8498	0.7901	0.8105	0.6386	0.7717
ACGT-flank	CGTACGTACC	0.7843	0.6749	0.7945	0.6113	0.6755	0.7033
ACGT-flank	CGTACGTACG	0.9353	0.7496	0.8016	0.9067	0.8988	0.8874
ACGT-flank	CGTACGTAGA	0.8332	0.8509	0.7290	0.7693	0.7129	0.8146
ACGT-flank	CGTACGTAGC	1.3026	1.2550	0.9964	1.1373	1.0580	1.1160
ACGT-flank	CGTACGTAGG	1.0139	1.0298	0.9679	1.2091	1.0734	1.0362
ACGT-flank	CGTACGTAGT	0.7264	0.7832	0.8634	0.7247	0.9675	0.9938
ACGT-flank	CGTACGTATG	0.7722	0.9256	1.0986	0.9118	0.9614	0.9731
ACGT-flank	CGTACGTCAC	0.9848	1.2314	0.9689	0.8207	0.9004	0.8318
ACGT-flank	CGTACGTCAG	1.1603	1.0523	0.9013	1.3528	1.0871	0.8998
ACGT-flank	CGTACGTCCA	0.9621	1.1343	0.9937	0.8959	0.9892	1.1029
ACGT-flank	CGTACGTCCC	1.1794	1.3059	1.1903	1.3896	1.3685	1.2525
ACGT-flank	CGTACGTCCG	0.8059	0.9398	1.0580	0.8742	1.0051	0.9176
ACGT-flank	CGTACGTCCT	0.7094	0.9209	0.9535	0.8344	0.7985	0.6228
ACGT-flank	CGTACGTCGA	0.8283	0.9874	1.0812	0.7766	0.7698	0.7749
ACGT-flank	CGTACGTCGC	0.8438	1.0066	0.8820	0.8044	0.8037	0.8863
ACGT-flank	CGTACGTCGG	0.7958	0.8580	0.8898	0.8507	0.8188	0.8141
ACGT-flank	CGTACGTCGT	0.7637	0.7685	0.8912	0.7952	0.9051	0.8440
ACGT-flank	CGTACGTCTA	1.0104	1.0936	1.0110	0.9264	1.1302	1.0045
ACGT-flank	CGTACGTCTG	1.0339	1.2318	1.0043	1.1038	0.9681	1.0931

ACGT-flank	CGTACGTGAA	0.6863	0.7874	0.6733	0.6604	0.7549	0.7650
ACGT-flank	CGTACGTGAC	0.7948	1.0356	0.9008	0.7874	0.8111	0.8536
ACGT-flank	CGTACGTGAG	0.8126	0.7591	0.7612	0.9202	0.9174	0.9106
ACGT-flank	CGTACGTGAT	1.0461	1.2689	0.9668	0.7054	0.8105	1.1091
ACGT-flank	CGTACGTGCA	0.9705	0.7416	0.7967	0.8895	0.8152	0.7861
ACGT-flank	CGTACGTGCC	1.0168	0.8057	0.8697	0.8770	0.8162	0.9415
ACGT-flank	CGTACGTGCG	0.8991	1.0182	0.8872	0.8629	0.8963	0.8693
ACGT-flank	CGTACGTGCT	1.0153	0.9407	1.0044	0.9402	0.9469	0.9641
ACGT-flank	CGTACGTGGA	0.9114	0.9690	0.7959	0.9318	0.8797	0.9668
ACGT-flank	CGTACGTGGC	1.0012	1.1396	1.1264	0.9308	1.1420	1.0947
ACGT-flank	CGTACGTGGG	1.2997	1.4553	1.4382	1.4473	1.4089	1.4010
ACGT-flank	CGTACGTGGT	0.8345	0.9195	0.7028	0.7802	0.8895	0.8483
ACGT-flank	CGTACGTGTA	0.6428	0.8702	0.9257	0.8211	1.1671	0.8576
ACGT-flank	CGTACGTGTC	1.3517	1.4277	1.2384	1.4950	1.5609	1.5831
ACGT-flank	CGTACGTGTG	1.2516	1.2063	1.2556	1.1855	1.2757	1.1715
ACGT-flank	CGTACGTTAG	0.9570	0.9622	1.2418	1.0307	1.2265	1.1130
ACGT-flank	CGTACGTTCC	0.8879	0.9445	0.9101	0.7096	0.9093	0.9084
ACGT-flank	CGTACGTTCG	0.8023	0.7711	0.7323	0.9089	0.8610	0.8014
ACGT-flank	CGTACGTTGA	0.9156	0.7517	0.8608	0.8168	0.8605	0.7904
ACGT-flank	CGTACGTTGC	1.1745	1.0418	1.0976	0.9182	0.9984	1.2453
ACGT-flank	CGTACGTTGG	1.0146	0.9884	1.0196	1.1425	1.0836	1.0553
ACGT-flank	CTAACGTAAG	1.4164	1.4212	1.3904	1.2226	1.2845	1.3039
ACGT-flank	CTAACGTACA	0.7078	0.5612	0.8664	0.7751	0.8315	0.7404
ACGT-flank	CTAACGTACG	0.6214	0.4114	0.8260	0.5569	0.3637	0.5413
ACGT-flank	CTAACGTAGA	0.7792	1.1178	0.7905	0.6944	0.8298	0.9717
ACGT-flank	CTAACGTAGC	0.3849	0.5168	0.6501	0.5494	0.5158	0.7366
ACGT-flank	CTAACGTAGG	1.0403	0.9421	1.0896	0.9881	1.0344	1.0638
ACGT-flank	CTAACGTCAA	0.8180	0.6791	0.5778	0.8046	0.8446	0.7584
ACGT-flank	CTAACGTCCA	1.1840	1.0862	1.0579	0.8253	0.8431	0.9486
ACGT-flank	CTAACGTCCC	0.9168	1.0348	0.9925	0.9180	0.9336	0.8999
ACGT-flank	CTAACGTCCG	0.8634	0.9000	0.8560	0.8306	1.0562	0.8558
ACGT-flank	CTAACGTCGA	0.8462	0.9409	1.1089	0.5540	0.7974	0.7754
ACGT-flank	CTAACGTCGC	0.9936	0.8688	0.8662	0.8310	0.8282	0.9229
ACGT-flank	CTAACGTCGG	0.7523	0.7574	0.7898	0.9119	0.6900	0.7441
ACGT-flank	CTAACGTCGT	0.5975	1.0275	0.9680	0.6316	0.8111	0.7065
ACGT-flank	CTAACGTGAA	0.9740	0.9887	0.5028	0.8755	1.2786	1.1558
ACGT-flank	CTAACGTGAG	0.7636	0.7801	0.6533	0.7944	0.6715	0.7976
ACGT-flank	CTAACGTGCA	0.9720	0.7330	1.1599	1.0316	0.9119	1.0011
ACGT-flank	CTAACGTGCC	1.0016	0.9323	0.7118	0.8257	0.9766	0.9620
ACGT-flank	CTAACGTGCG	0.9191	0.9770	0.7695	0.9753	0.9294	0.9241
ACGT-flank	CTAACGTGCT	0.9693	1.1811	1.0774	1.0053	1.0524	1.0025
ACGT-flank	CTAACGTGGA	1.1313	1.1569	1.0398	1.0237	1.0778	1.0390
ACGT-flank	CTAACGTGGC	1.1113	1.0231	1.1222	1.1421	0.9200	0.9267
ACGT-flank	CTAACGTGGG	1.4557	1.4574	1.3603	1.4076	1.3714	1.3789

ACGT-flank	CTAACGTGGT	1.0514	0.8902	0.8664	0.8999	0.9434	0.9440
ACGT-flank	CTAACGTGTC	1.1391	1.0136	0.7344	0.8060	0.6800	0.9062
ACGT-flank	CTAACGTGTG	1.4352	1.4573	1.1219	1.3453	1.3373	1.3890
ACGT-flank	CTAACGTTCC	1.1701	1.3378	1.1745	0.7660	1.0397	1.2205
ACGT-flank	CTAACGTTGA	0.8777	1.3700	0.9425	0.8716	0.8946	0.9986
ACGT-flank	CTAACGTTGG	0.9017	0.9745	1.0919	1.0423	1.1147	1.0165
ACGT-flank	CTCACGTAAC	0.8350	0.7737	0.8600	0.9175	0.7770	0.7896
ACGT-flank	CTCACGTAAG	1.1575	1.3534	1.0617	0.8371	0.7884	0.9320
ACGT-flank	CTCACGTACA	0.6935	0.4450	0.7175	0.7845	0.7164	0.7429
ACGT-flank	CTCACGTACC	0.7650	0.9970	0.9077	0.9194	0.8827	0.8465
ACGT-flank	CTCACGTACG	0.9671	0.9928	0.9846	1.2059	1.0261	1.0543
ACGT-flank	CTCACGTAGA	1.1540	1.0329	1.0596	1.0086	1.1668	0.9718
ACGT-flank	CTCACGTAGC	0.8834	0.7400	0.9025	0.8574	1.0260	0.9468
ACGT-flank	CTCACGTAGG	1.3388	1.2169	1.2654	1.2821	1.5156	1.3979
ACGT-flank	CTCACGTAGT	0.8090	0.8067	0.6920	0.6369	0.8388	0.8920
ACGT-flank	CTCACGTATG	0.8996	0.9767	1.1437	1.0288	0.9530	0.9255
ACGT-flank	CTCACGTCAG	1.0738	1.1590	1.1800	0.8120	1.1477	1.0628
ACGT-flank	CTCACGTCCA	0.8359	0.7640	1.0433	0.9909	0.9162	1.1104
ACGT-flank	CTCACGTCCC	0.9859	0.9352	0.9347	1.0350	0.9344	0.9320
ACGT-flank	CTCACGTCCG	0.7128	0.9926	0.7536	0.9051	0.7965	0.8549
ACGT-flank	CTCACGTCCT	1.0573	1.0800	1.1481	0.9119	1.1112	1.0222
ACGT-flank	CTCACGTCGA	0.9733	0.8855	0.8291	0.7805	0.8464	0.9683
ACGT-flank	CTCACGTCGC	0.8814	0.8877	0.7673	0.7051	0.9608	0.7995
ACGT-flank	CTCACGTCGG	1.1009	0.9049	1.0778	1.0826	0.9846	1.1732
ACGT-flank	CTCACGTCGT	1.2436	1.2382	1.1324	1.2685	1.0283	1.2159
ACGT-flank	CTCACGTCTC	1.2352	0.9613	1.0843	0.9792	1.0569	0.8955
ACGT-flank	CTCACGTCTG	1.0303	1.3330	1.1702	1.0840	1.0602	1.0737
ACGT-flank	CTCACGTGAA	0.6919	0.8320	0.6666	0.9823	0.6186	0.7277
ACGT-flank	CTCACGTGAC	0.7918	0.9868	0.8872	0.9349	0.8320	0.8862
ACGT-flank	CTCACGTGAG	1.0768	1.0674	0.9444	1.0576	1.0061	1.1069
ACGT-flank	CTCACGTGCC	0.9455	0.8201	1.1638	1.0897	1.0263	0.9720
ACGT-flank	CTCACGTGCG	1.2229	1.0076	1.2587	1.1153	1.1245	1.2189
ACGT-flank	CTCACGTGCT	1.0913	1.0539	1.2893	1.1066	1.1335	1.0017
ACGT-flank	CTCACGTGGA	0.9729	1.0355	1.0684	1.1023	1.1178	1.1090
ACGT-flank	CTCACGTGGC	1.0133	1.1091	1.0277	1.1284	0.9835	1.1110
ACGT-flank	CTCACGTGGG	1.3682	1.2944	1.2536	1.3063	1.3912	1.1772
ACGT-flank	CTCACGTGGT	0.8783	1.1040	0.8812	1.0956	0.8576	0.9817
ACGT-flank	CTCACGTGTC	0.7756	0.9988	0.7729	0.8625	0.7664	0.7173
ACGT-flank	CTCACGTGTG	0.9215	1.0647	1.1139	0.9638	1.0605	0.9149
ACGT-flank	CTCACGTTAG	0.9874	1.3841	0.5193	0.8079	0.6424	1.1466
ACGT-flank	CTCACGTTCC	0.7695	0.7315	0.7580	0.8317	0.6422	0.7250
ACGT-flank	CTCACGTTCG	0.8290	0.8587	0.7249	0.7096	0.8738	0.9647
ACGT-flank	CTCACGTTGA	0.9839	1.2486	1.1825	0.8079	1.0565	1.0898
ACGT-flank	CTCACGTTGG	1.0524	0.9569	1.3065	1.0502	1.0378	1.0809

ACGT-flank	CTGACGTAAG	1.0369	0.9043	0.8543	0.8603	0.8882	0.8593
ACGT-flank	CTGACGTACA	0.8535	0.7624	0.6673	0.7264	0.6202	0.7437
ACGT-flank	CTGACGTACC	0.9456	1.1191	0.9506	0.8982	0.8580	0.8904
ACGT-flank	CTGACGTACG	1.2087	1.0483	0.8601	1.4111	0.9048	1.1594
ACGT-flank	CTGACGTACT	0.6490	1.0000	0.9329	1.0508	0.6948	0.8543
ACGT-flank	CTGACGTAGA	0.8837	0.9866	0.8641	0.7413	0.8169	0.7722
ACGT-flank	CTGACGTAGC	0.9478	1.0429	0.9483	0.9411	0.8553	0.8415
ACGT-flank	CTGACGTAGG	0.9223	0.8546	1.0053	1.1039	0.8944	0.9515
ACGT-flank	CTGACGTAGT	1.0134	0.8793	0.7990	0.9397	0.8366	0.8898
ACGT-flank	CTGACGTATC	0.9315	0.8403	0.9803	1.0469	0.9086	1.0273
ACGT-flank	CTGACGTATG	0.9881	0.8533	0.9526	0.9247	0.9302	0.9839
ACGT-flank	CTGACGTCAC	1.0849	0.9828	0.9511	0.8190	1.0246	0.8985
ACGT-flank	CTGACGTCAG	1.3889	1.2134	1.2894	1.5855	1.2639	1.3885
ACGT-flank	CTGACGTCAT	0.8885	1.0553	1.0344	1.0867	1.0137	0.8999
ACGT-flank	CTGACGTCCC	3.8562	4.4812	3.8674	4.0465	4.0418	3.5645
ACGT-flank	CTGACGTCCG	0.7847	0.8012	0.7824	0.7745	0.7865	0.8309
ACGT-flank	CTGACGTCCT	1.0250	1.0109	1.4192	0.9333	1.2613	1.3307
ACGT-flank	CTGACGTCGA	0.7702	0.6870	0.7748	0.7719	0.7384	0.8181
ACGT-flank	CTGACGTCGG	0.8712	0.8776	0.7848	0.8913	0.8592	0.9063
ACGT-flank	CTGACGTCGT	1.0310	1.2253	1.4871	1.2747	1.0903	1.2329
ACGT-flank	CTGACGTCTA	0.8003	1.0424	0.6536	0.6937	1.0541	0.8445
ACGT-flank	CTGACGTCTC	0.9781	0.9967	1.0096	0.9551	0.8359	0.9873
ACGT-flank	CTGACGTCTG	0.9456	1.0424	0.6049	0.8100	0.9120	0.9179
ACGT-flank	CTGACGTGAA	0.6506	0.8088	0.7196	0.6262	0.6488	0.8581
ACGT-flank	CTGACGTGAC	0.6199	0.7561	0.6551	0.8248	0.6673	0.8049
ACGT-flank	CTGACGTGAG	0.9420	0.9104	1.0848	0.9358	0.9537	1.0454
ACGT-flank	CTGACGTGAT	0.9344	1.1167	0.7732	0.8446	1.1537	0.9843
ACGT-flank	CTGACGTGCA	1.0152	0.8703	0.9759	0.7204	0.9228	0.9175
ACGT-flank	CTGACGTGCC	0.9234	1.1989	1.0574	0.9466	0.9416	0.9363
ACGT-flank	CTGACGTGCG	0.7006	0.9857	0.6995	0.9646	0.9541	0.8614
ACGT-flank	CTGACGTGCT	1.4421	1.0330	1.0943	1.0041	1.2508	1.0094
ACGT-flank	CTGACGTGGA	1.0262	1.2689	1.1527	1.1185	1.1863	0.9557
ACGT-flank	CTGACGTGGC	1.3710	1.1814	1.2776	1.6992	1.4532	1.3168
ACGT-flank	CTGACGTGGG	1.2547	1.2237	1.2198	1.0407	1.0990	1.0968
ACGT-flank	CTGACGTGGT	1.1307	1.2256	1.0716	1.1678	1.0889	1.1133
ACGT-flank	CTGACGTGTA	1.2123	0.9739	1.4178	1.3904	1.1883	1.1783
ACGT-flank	CTGACGTGTC	1.3215	1.3819	1.4334	1.4989	1.4179	1.4337
ACGT-flank	CTGACGTGTG	1.2081	1.2151	1.0950	1.3416	1.2521	1.1982
ACGT-flank	CTGACGTGTT	0.9525	0.9200	0.9344	1.3868	1.3153	1.0454
ACGT-flank	CTGACGTTAC	0.7669	0.8327	0.8908	0.6318	0.6851	0.6424
ACGT-flank	CTGACGTTAG	1.0854	0.7196	1.0487	0.8732	0.7881	0.7511
ACGT-flank	CTGACGTTCC	1.0756	0.9424	0.9245	0.9809	1.0480	0.9108
ACGT-flank	CTGACGTTCG	0.9874	0.7996	0.7549	0.9090	0.9406	0.9891
ACGT-flank	CTGACGTTGA	0.9912	0.8806	1.2176	1.0412	0.9320	1.0981

ACGT-flank	CTGACGTTGC	1.1745	0.7793	0.8871	1.0311	1.1508	1.1349
ACGT-flank	CTGACGTTGG	0.8557	0.8496	0.9114	0.8552	0.9304	0.8544
ACGT-flank	CTGACGTTGT	0.8630	0.6872	0.7994	0.7972	1.0342	0.8097
ACGT-flank	CTGACGTTTG	0.6312	0.9251	0.7935	0.9011	0.9626	0.8893
ACGT-flank	CTTACGTACG	0.7207	0.6555	0.7738	0.6755	1.0340	0.8128
ACGT-flank	CTTACGTAGG	1.1518	1.0967	0.9610	0.9001	0.9487	0.9483
ACGT-flank	CTTACGTCAG	1.1082	1.0017	0.7189	0.9342	0.7145	0.6926
ACGT-flank	CTTACGTCCC	0.7659	1.0098	0.9715	0.8630	0.8265	0.8778
ACGT-flank	CTTACGTCCG	0.8252	0.5759	0.7412	0.6490	0.6471	0.8247
ACGT-flank	CTTACGTCGA	1.2789	0.7933	0.9972	0.7215	1.1347	1.0003
ACGT-flank	CTTACGTCGC	0.7984	1.0097	0.8938	0.8054	0.8732	0.8999
ACGT-flank	CTTACGTCGG	0.7662	0.6560	0.8932	0.9099	0.8707	0.9413
ACGT-flank	CTTACGTGAG	0.7039	0.7420	0.5867	0.7822	0.7364	0.8094
ACGT-flank	CTTACGTGCC	0.9417	0.7838	1.1772	0.8048	0.9261	0.9003
ACGT-flank	CTTACGTGCG	0.9989	0.7829	1.0076	0.7233	1.0420	0.9305
ACGT-flank	CTTACGTGGA	1.0171	1.1355	0.9224	0.9757	0.9987	1.0733
ACGT-flank	CTTACGTGGC	1.1741	1.0645	1.1210	1.2561	1.2844	1.1802
ACGT-flank	CTTACGTGGG	1.3047	1.4122	1.3067	1.3746	1.3301	1.2481
ACGT-flank	CTTACGTGGT	0.6766	1.2779	1.0146	1.5241	1.1510	0.8798
ACGT-flank	CTTACGTGTG	1.0012	0.9949	0.9766	1.0747	1.0510	1.1837
ACGT-flank	CTTACGTTGG	0.8382	1.0984	0.7715	0.8881	0.8131	1.0290
ACGT-flank	GAAACGTAAA	0.8982	0.9861	0.9119	1.0090	0.8495	0.8697
ACGT-flank	GAAACGTAAC	0.6008	0.8602	0.7708	0.7347	0.7070	0.8217
ACGT-flank	GAAACGTAAG	0.8999	0.9086	0.8445	0.8356	0.7871	0.9596
ACGT-flank	GAAACGTACA	0.9329	0.8699	0.6859	0.7447	0.8520	0.8230
ACGT-flank	GAAACGTACC	0.8115	0.8571	0.6210	0.6655	0.6584	0.7917
ACGT-flank	GAAACGTACG	0.8022	0.8100	0.8419	0.7649	0.8144	0.8575
ACGT-flank	GAAACGTACT	0.7328	0.7161	0.6921	0.7138	0.9550	0.8196
ACGT-flank	GAAACGTAGA	1.0739	1.1914	1.1637	0.9247	1.1937	0.9887
ACGT-flank	GAAACGTAGC	1.1066	0.8958	1.0352	1.0225	1.2451	1.2368
ACGT-flank	GAAACGTAGG	0.8417	1.0523	0.9713	0.9356	0.8381	0.8053
ACGT-flank	GAAACGTAGT	0.7525	0.6468	0.9192	0.7936	0.7197	0.7781
ACGT-flank	GAAACGTATC	0.4646	0.7008	0.6821	0.4692	0.7198	0.5861
ACGT-flank	GAAACGTATG	0.8664	0.8075	0.9489	0.7468	0.7660	0.7941
ACGT-flank	GAAACGTCAA	0.8401	0.8240	0.9147	0.7270	1.0857	0.9448
ACGT-flank	GAAACGTCAC	0.7514	0.9466	0.6322	0.7540	0.7743	0.7723
ACGT-flank	GAAACGTCAG	0.6971	0.6476	0.5528	0.7075	0.7012	0.7208
ACGT-flank	GAAACGTCAT	0.4023	0.8606	0.6224	0.5123	0.6341	0.6482
ACGT-flank	GAAACGTCCG	0.5770	0.7965	0.7375	0.6526	0.8350	0.8016
ACGT-flank	GAAACGTCGA	0.7366	0.7995	0.7582	0.8534	0.9308	0.8908
ACGT-flank	GAAACGTCGC	0.7820	0.6931	0.7308	0.7827	0.6129	0.5579
ACGT-flank	GAAACGTCGG	1.0430	1.0135	1.0691	0.9257	0.8446	0.9388
ACGT-flank	GAAACGTCGT	0.6476	0.8753	0.6448	0.6992	0.9838	0.7178
ACGT-flank	GAAACGTCTA	1.4438	1.7491	1.2532	0.6508	1.1840	1.2513

ACGT-flank	GAAACGTCTC	0.7815	0.7705	0.7882	0.9024	0.7719	0.9139
ACGT-flank	GAAACGTCTG	1.1854	1.2603	1.2099	0.8407	1.0776	1.2581
ACGT-flank	GAAACGTGAA	0.7635	0.9961	0.8594	0.5724	0.8494	0.8606
ACGT-flank	GAAACGTGAC	0.5615	0.4392	0.4755	0.6116	0.5226	0.4624
ACGT-flank	GAAACGTGAG	0.7782	0.9362	0.9842	0.9707	0.9134	0.9371
ACGT-flank	GAAACGTGAT	0.7357	0.7657	0.5716	0.9352	0.7214	0.6928
ACGT-flank	GAAACGTGCA	0.7784	0.7817	1.0222	1.0615	0.8556	0.8169
ACGT-flank	GAAACGTGCC	0.6510	1.1136	0.8498	0.8970	0.7180	0.9152
ACGT-flank	GAAACGTGCG	0.7838	0.9097	0.9130	1.1512	0.8570	0.8855
ACGT-flank	GAAACGTGCT	0.7031	0.8661	0.7887	0.7226	0.6352	0.7239
ACGT-flank	GAAACGTGGA	0.9661	0.8432	0.7801	0.8334	1.0776	0.8556
ACGT-flank	GAAACGTGGC	1.0830	0.8374	0.9754	1.0951	0.9983	1.1176
ACGT-flank	GAAACGTGGG	1.2252	1.0247	1.1310	1.1862	1.1059	1.0906
ACGT-flank	GAAACGTGGT	0.9078	0.9345	0.8752	0.8358	0.8933	0.8279
ACGT-flank	GAAACGTGTA	0.9423	0.9087	0.8528	0.7983	0.8683	0.8530
ACGT-flank	GAAACGTGTC	0.8826	0.9199	0.9803	0.9057	0.8337	0.9280
ACGT-flank	GAAACGTGTG	1.2814	1.3632	1.3807	1.2193	1.3209	1.2941
ACGT-flank	GAAACGTGTT	0.9191	1.1803	1.1835	1.0495	1.1543	0.9553
ACGT-flank	GAAACGTTAA	0.5849	0.3253	0.8385	0.7095	0.6119	0.6622
ACGT-flank	GAAACGTTAG	0.8299	0.9322	0.8147	0.7573	0.7703	0.8216
ACGT-flank	GAAACGTTCC	0.6039	0.7427	0.6289	0.6745	0.6343	0.6854
ACGT-flank	GAAACGTTCG	0.7461	0.8713	0.7640	0.7807	0.7882	0.8152
ACGT-flank	GAAACGTTGA	0.7276	0.7696	0.9676	0.8593	0.8713	0.8735
ACGT-flank	GAAACGTTGC	0.9448	0.8962	1.1200	0.9152	0.8422	0.8594
ACGT-flank	GAAACGTTGG	0.8390	0.8523	0.8137	0.7757	0.7646	0.8190
ACGT-flank	GAAACGTTGT	1.3507	1.5312	1.4460	1.2975	1.3974	1.4045
ACGT-flank	GAAACGTTTG	0.7544	0.4216	0.6483	0.5178	0.6511	0.7019
ACGT-flank	GACACGTAAA	0.8780	1.1014	0.7667	0.8252	1.0957	0.8507
ACGT-flank	GACACGTAAC	0.7725	0.5786	0.6207	0.6972	0.9066	0.7891
ACGT-flank	GACACGTAAG	0.8295	0.7736	1.0566	0.8515	0.9921	0.9461
ACGT-flank	GACACGTACA	0.8823	0.9117	0.8265	0.8250	0.8550	1.0272
ACGT-flank	GACACGTACC	0.9582	1.3325	1.0418	1.0882	0.8479	0.8921
ACGT-flank	GACACGTACG	0.8173	0.9504	0.6608	0.8119	0.8318	0.8965
ACGT-flank	GACACGTACT	0.6377	0.5431	0.5687	0.8113	0.4543	0.6425
ACGT-flank	GACACGTAGA	0.9187	0.8239	1.0406	1.1328	1.0395	0.9589
ACGT-flank	GACACGTAGC	0.9252	1.0145	1.0215	0.8934	0.7947	0.9273
ACGT-flank	GACACGTAGG	1.0791	1.0182	1.1817	0.9614	1.0080	0.9863
ACGT-flank	GACACGTAGT	0.8484	1.0241	0.8306	0.7970	0.9634	0.9370
ACGT-flank	GACACGTATC	0.5612	0.7691	0.7607	1.0613	0.7532	0.8142
ACGT-flank	GACACGTATG	0.8676	1.0764	0.8511	0.8344	0.9271	0.9075
ACGT-flank	GACACGTCAA	0.6114	0.6101	0.8269	0.7584	0.6416	0.4727
ACGT-flank	GACACGTCAC	0.9465	0.6894	0.9396	0.8516	1.0137	0.8936
ACGT-flank	GACACGTCAG	1.0568	0.8107	1.0300	0.9154	0.8939	0.8897
ACGT-flank	GACACGTCAT	0.9082	0.9648	0.8314	1.0990	1.1293	0.9867

ACGT-flank	GACACGTCCA	0.8881	0.8258	1.1818	0.9309	0.8118	0.9616
ACGT-flank	GACACGTCCC	1.0803	0.7069	1.1031	0.6153	1.0266	0.9119
ACGT-flank	GACACGTCCG	0.6253	0.6871	0.7167	0.5985	0.8862	0.7125
ACGT-flank	GACACGTCCT	0.9784	1.1452	0.9004	1.0379	1.0414	0.9452
ACGT-flank	GACACGTCGA	0.8245	0.9732	0.7121	0.7672	0.9309	0.8653
ACGT-flank	GACACGTCGG	1.1060	0.8722	0.8500	1.0135	0.9656	0.8341
ACGT-flank	GACACGTCGT	1.0021	0.8306	1.0225	0.8026	0.8401	0.9180
ACGT-flank	GACACGTCTA	0.4327	0.9921	0.4729	0.6294	0.7081	0.7856
ACGT-flank	GACACGTCTC	0.9454	1.1774	1.5632	1.0534	1.0813	1.1810
ACGT-flank	GACACGTCTG	0.9965	0.8568	1.1673	1.0541	1.0054	0.9784
ACGT-flank	GACACGTGAA	0.9355	0.7735	0.9357	0.9183	0.9787	0.9600
ACGT-flank	GACACGTGAC	1.0624	0.8679	1.1687	0.9325	1.1818	0.9445
ACGT-flank	GACACGTGAG	0.8214	0.8183	1.0608	0.9052	0.8096	0.8487
ACGT-flank	GACACGTGAT	0.8227	0.8464	0.7984	0.7682	0.8897	0.7428
ACGT-flank	GACACGTGCA	0.9457	0.9409	0.9994	0.9840	1.0945	0.9823
ACGT-flank	GACACGTGCG	1.2070	1.2202	1.1161	1.0239	1.2034	1.1059
ACGT-flank	GACACGTGCT	1.1262	1.1373	0.7853	1.0586	0.9945	1.0293
ACGT-flank	GACACGTGGA	1.4974	1.6529	1.5516	2.0496	1.7449	1.3882
ACGT-flank	GACACGTGGC	2.1248	2.4300	2.1593	2.9304	2.6946	2.4054
ACGT-flank	GACACGTGGG	1.0852	1.2863	1.2562	1.2706	1.3299	1.2148
ACGT-flank	GACACGTGGT	1.1423	1.2556	1.1403	1.5960	1.5106	1.3324
ACGT-flank	GACACGTGTA	1.4240	1.2488	1.3849	1.8423	1.5111	1.5976
ACGT-flank	GACACGTGTC	1.7247	1.7506	1.6034	2.4341	2.2047	2.1594
ACGT-flank	GACACGTGTG	1.4601	1.3659	1.4687	1.5691	1.5923	1.4605
ACGT-flank	GACACGTGTT	1.5641	1.7173	1.6477	2.0871	1.9326	1.7712
ACGT-flank	GACACGTTAG	0.9062	0.8929	0.8713	0.8381	0.9247	0.8396
ACGT-flank	GACACGTTCG	0.7786	0.8586	0.8145	0.8954	0.8852	0.8558
ACGT-flank	GACACGTTGA	0.9521	0.7239	0.8025	0.8780	1.0685	0.9904
ACGT-flank	GACACGTTGC	0.8944	1.0091	0.9106	0.8070	1.1054	0.9820
ACGT-flank	GACACGTTGG	1.0129	0.9911	1.1375	0.9829	1.0590	0.9918
ACGT-flank	GACACGTTGT	0.9256	0.8880	0.9206	0.9918	1.0246	0.8917
ACGT-flank	GACACGTTTG	0.9935	0.9071	1.0317	1.0339	1.0880	1.0175
ACGT-flank	GAGACGTAAA	0.8391	0.9473	0.7717	0.7118	0.8206	0.8901
ACGT-flank	GAGACGTAAC	0.8992	0.9204	1.0640	0.8764	1.0788	0.9191
ACGT-flank	GAGACGTAAG	0.8350	1.0220	0.6692	0.8076	0.7905	0.8268
ACGT-flank	GAGACGTAAT	0.9434	1.1284	0.9414	0.9726	1.1736	0.9678
ACGT-flank	GAGACGTACA	0.6879	0.7251	0.9867	0.6952	0.7121	0.7251
ACGT-flank	GAGACGTACC	0.9662	0.8336	1.0333	0.8191	0.9259	0.9638
ACGT-flank	GAGACGTACG	0.7492	1.0767	0.9849	0.7574	1.1568	0.8663
ACGT-flank	GAGACGTACT	0.7394	0.8702	0.8592	0.6886	0.7510	0.7791
ACGT-flank	GAGACGTAGA	0.9140	0.9552	0.9626	1.1590	0.9871	1.0576
ACGT-flank	GAGACGTAGC	0.9583	0.7633	0.9570	0.6921	0.8580	0.9939
ACGT-flank	GAGACGTAGG	1.0806	1.0181	1.0949	0.9665	1.0101	0.9957
ACGT-flank	GAGACGTAGT	1.0768	0.8983	0.9389	0.8281	1.0241	0.9309

ACGT-flank	GAGACGTATA	0.8641	1.1150	0.8644	0.9396	0.9913	0.6511
ACGT-flank	GAGACGTATC	1.1217	1.1657	1.2264	1.0004	1.2447	1.1565
ACGT-flank	GAGACGTATG	0.9520	0.9519	1.0721	0.9632	0.9421	1.0332
ACGT-flank	GAGACGTCAA	0.5408	0.8839	0.5802	0.5876	0.7460	0.7725
ACGT-flank	GAGACGTCAC	0.8848	0.6665	0.7478	0.6565	0.8420	0.6535
ACGT-flank	GAGACGTCAG	0.9744	1.0471	1.3430	0.9984	1.2551	1.2303
ACGT-flank	GAGACGTCAT	1.0671	0.7367	0.7882	0.9151	0.7770	0.7899
ACGT-flank	GAGACGTCCC	0.7370	0.8411	1.1667	0.7628	0.8549	0.7432
ACGT-flank	GAGACGTCCG	0.7269	0.7458	1.0942	1.1095	0.7650	0.9400
ACGT-flank	GAGACGTCCT	1.5820	1.9021	1.4929	1.5804	1.6664	1.4720
ACGT-flank	GAGACGTCGA	0.8535	0.9632	0.7908	0.7726	0.7350	0.7180
ACGT-flank	GAGACGTCGC	0.7986	0.9693	0.5697	0.6917	0.8236	0.9539
ACGT-flank	GAGACGTCGG	0.7890	1.0970	0.6497	0.7511	0.8457	0.7826
ACGT-flank	GAGACGTCGT	0.9590	0.9617	0.7838	1.1240	0.9553	0.8386
ACGT-flank	GAGACGTCTA	0.9902	1.0442	0.9590	1.0787	0.8879	1.0003
ACGT-flank	GAGACGTCTC	0.9472	0.6717	0.6417	0.7781	0.8381	0.8405
ACGT-flank	GAGACGTCTG	0.6361	0.9109	0.6821	0.6445	0.6420	0.7191
ACGT-flank	GAGACGTGAA	0.6711	0.5931	0.7094	1.0909	1.0507	0.7706
ACGT-flank	GAGACGTGAC	1.0928	0.8499	0.7040	0.8135	0.8526	0.9811
ACGT-flank	GAGACGTGAG	1.2165	1.1487	1.1808	1.0415	1.1480	1.1133
ACGT-flank	GAGACGTGAT	0.8420	1.0483	0.8656	0.9581	1.0055	1.0450
ACGT-flank	GAGACGTGCA	0.6463	0.7027	0.9383	0.8646	0.9211	0.9751
ACGT-flank	GAGACGTGCG	0.9264	0.9463	0.8336	0.9027	0.8139	0.7626
ACGT-flank	GAGACGTGCT	0.8717	0.7992	1.0325	0.8189	0.9144	0.9151
ACGT-flank	GAGACGTGGA	1.1387	1.2218	1.0289	1.0757	1.0309	1.0253
ACGT-flank	GAGACGTGGC	0.5403	0.6236	0.7887	0.5987	0.7551	0.6559
ACGT-flank	GAGACGTGGG	1.0221	1.1386	1.0695	1.1982	1.0792	1.0611
ACGT-flank	GAGACGTGGT	1.0469	1.0227	1.0620	0.9451	1.0045	1.0007
ACGT-flank	GAGACGTGTA	1.0205	0.9200	0.8104	0.8827	0.8229	0.9767
ACGT-flank	GAGACGTGTC	0.7035	0.9626	0.8471	0.8846	0.8536	0.8477
ACGT-flank	GAGACGTGTG	1.0810	1.0466	0.8868	1.1910	1.0984	1.1537
ACGT-flank	GAGACGTGTT	1.2259	1.1294	1.1903	1.1064	1.1561	1.1413
ACGT-flank	GAGACGTTAA	0.7433	0.8088	1.0109	0.9454	0.8335	0.8700
ACGT-flank	GAGACGTTAC	1.1065	0.9489	0.8612	0.7518	0.6109	0.8800
ACGT-flank	GAGACGTTAG	0.8716	1.0035	0.9429	0.8549	0.8342	0.9071
ACGT-flank	GAGACGTTCA	1.1441	0.9820	1.1848	1.1423	1.1949	1.1455
ACGT-flank	GAGACGTTCC	1.4642	1.3397	0.8942	1.0545	1.3436	0.9583
ACGT-flank	GAGACGTTCG	1.0318	0.9344	0.9722	0.9503	0.8097	0.9241
ACGT-flank	GAGACGTTCT	0.8347	0.7783	0.9641	0.5835	0.7272	0.6586
ACGT-flank	GAGACGTTGA	0.6704	0.6179	0.8189	0.8504	0.8491	0.8107
ACGT-flank	GAGACGTTGC	0.9075	0.8130	0.6230	0.6622	0.7118	0.7657
ACGT-flank	GAGACGTTGG	1.0622	0.9935	1.0229	0.9310	1.0743	1.0523
ACGT-flank	GAGACGTTGT	0.9731	1.0883	1.0554	0.9675	0.9349	0.9658
ACGT-flank	GAGACGTTTG	1.1579	1.1515	1.1664	1.1647	1.1311	1.2669

ACGT-flank	GATACGTACG	0.4855	0.4659	0.4278	0.7004	0.5530	0.4349
ACGT-flank	GATACGTAGA	0.8217	1.0519	1.0691	1.0372	1.0185	0.9466
ACGT-flank	GATACGTAGC	0.8278	0.6643	0.9079	0.7920	0.8610	0.9157
ACGT-flank	GATACGTAGG	1.2612	1.2956	1.1719	1.4135	1.2968	1.1966
ACGT-flank	GATACGTAGT	0.7990	0.8644	1.0510	1.0305	1.1942	1.0757
ACGT-flank	GATACGTCAC	0.8522	1.1674	1.0948	1.0748	0.8657	0.8586
ACGT-flank	GATACGTCAG	0.9791	0.8590	1.0303	0.8876	1.0322	0.9548
ACGT-flank	GATACGTCCA	1.0268	1.2475	0.8103	1.0275	1.1045	1.0457
ACGT-flank	GATACGTCCC	0.7344	1.0162	0.8648	1.1256	0.7968	0.9804
ACGT-flank	GATACGTCCG	0.9990	1.0053	1.0555	0.9471	0.9077	0.8313
ACGT-flank	GATACGTCCT	0.7925	0.8708	0.6507	0.9788	0.5894	0.7772
ACGT-flank	GATACGTCGA	1.1138	1.3173	0.8166	0.6606	1.0241	0.7246
ACGT-flank	GATACGTCGC	0.8422	1.0089	0.8382	0.8356	0.8400	0.8515
ACGT-flank	GATACGTCGG	1.1199	1.1784	1.1323	1.2233	1.0525	1.0911
ACGT-flank	GATACGTCGT	0.9783	1.1102	0.8720	0.8947	0.9807	0.9803
ACGT-flank	GATACGTCTG	1.3539	1.2174	1.4036	1.1802	1.2675	1.3199
ACGT-flank	GATACGTGAA	0.7716	0.9993	0.9368	0.7577	0.9825	0.8420
ACGT-flank	GATACGTGAC	0.8841	1.0315	0.8073	0.7805	0.9359	0.9051
ACGT-flank	GATACGTGAG	0.7940	0.8467	0.6852	0.9105	0.7396	0.7800
ACGT-flank	GATACGTGCA	0.8790	0.6575	1.0321	1.1944	0.9847	0.9651
ACGT-flank	GATACGTGCC	0.9706	0.9136	0.8471	0.8095	0.8267	0.9122
ACGT-flank	GATACGTGCG	0.9985	1.0719	1.0792	1.0405	0.9509	1.0227
ACGT-flank	GATACGTGCT	0.9698	1.0495	1.0619	0.9911	1.0222	1.2083
ACGT-flank	GATACGTGGA	1.0023	0.9156	1.0385	1.0682	0.9923	1.1380
ACGT-flank	GATACGTGGC	1.2276	1.2390	1.3069	1.3591	1.2824	1.1933
ACGT-flank	GATACGTGGG	1.3453	1.3364	1.5560	1.3740	1.3962	1.4515
ACGT-flank	GATACGTGGT	0.9850	0.9114	0.9915	0.9292	0.9829	0.9292
ACGT-flank	GATACGTGTC	1.0168	1.0843	0.8160	1.2518	1.0279	1.0369
ACGT-flank	GATACGTGTG	1.4212	1.1180	1.2629	1.2391	1.1540	1.2645
ACGT-flank	GATACGTTCG	1.0893	0.9951	0.9769	0.7547	0.9895	0.9459
ACGT-flank	GATACGTTGA	0.9004	0.7248	0.7552	0.6795	0.6453	0.9838
ACGT-flank	GATACGTTGC	0.7023	0.8117	1.1368	0.6881	1.0035	0.9457
ACGT-flank	GATACGTTGG	1.2527	1.0894	1.0913	1.0122	1.2777	1.1659
ACGT-flank	GCAACGTAAA	0.5797	0.3702	0.4702	0.3877	0.4739	0.4340
ACGT-flank	GCAACGTAAG	0.8968	0.9372	0.9915	0.7897	0.9478	0.9228
ACGT-flank	GCAACGTACA	0.7608	0.9741	0.6417	0.5308	0.7573	0.5882
ACGT-flank	GCAACGTACC	0.9154	0.9911	0.9731	0.8842	0.9433	0.9237
ACGT-flank	GCAACGTACG	1.0345	0.9792	0.9683	0.9218	1.0056	1.2113
ACGT-flank	GCAACGTACT	1.3647	1.2978	1.5048	1.2559	1.5357	1.3154
ACGT-flank	GCAACGTAGA	1.0490	1.0041	1.0217	0.7923	0.8319	0.7557
ACGT-flank	GCAACGTAGG	1.2029	1.1071	1.0520	0.9875	1.0073	1.0315
ACGT-flank	GCAACGTAGT	0.9556	1.1589	0.9482	1.0400	0.9320	1.0483
ACGT-flank	GCAACGTATC	1.1105	1.0705	0.5949	0.7964	0.8694	0.8198
ACGT-flank	GCAACGTATG	0.9553	1.0093	0.9127	1.1053	0.9167	0.9617

ACGT-flank	GCAACGTCAC	0.5236	0.9925	0.8839	0.8253	1.0628	1.0858
ACGT-flank	GCAACGTCAG	0.9720	0.9443	0.6221	0.7921	0.7741	0.9974
ACGT-flank	GCAACGTCAT	0.8269	1.0628	1.1143	0.8293	0.9360	0.9401
ACGT-flank	GCAACGTCCA	1.0473	1.0037	1.0507	0.9660	0.9643	1.1909
ACGT-flank	GCAACGTCCG	0.7145	1.0377	0.7203	0.9217	0.8990	0.6900
ACGT-flank	GCAACGTCCT	0.7811	0.9769	0.9202	0.8410	0.8825	0.8619
ACGT-flank	GCAACGTCGA	0.9693	0.6154	0.8619	0.8209	0.9524	0.9563
ACGT-flank	GCAACGTCGG	1.1194	1.2787	0.8778	1.1024	1.0776	0.9978
ACGT-flank	GCAACGTCGT	0.7416	1.0279	0.8122	0.9610	0.8596	0.7760
ACGT-flank	GCAACGTCTA	0.6505	0.3195	0.4887	0.5863	0.5574	0.5395
ACGT-flank	GCAACGTCTC	1.0191	0.8253	0.9845	0.8947	0.9705	0.8873
ACGT-flank	GCAACGTCTG	0.8154	0.9822	0.8602	0.9127	0.8790	0.8737
ACGT-flank	GCAACGTGAA	0.8792	1.3588	1.3953	0.9072	0.9753	0.9760
ACGT-flank	GCAACGTGAC	0.6996	0.7586	0.7049	1.0711	0.6115	1.0614
ACGT-flank	GCAACGTGAG	0.9252	0.9456	1.0460	0.9278	1.0189	0.9616
ACGT-flank	GCAACGTGAT	0.8714	0.9209	1.1042	0.8013	0.9323	1.0629
ACGT-flank	GCAACGTGCA	0.9855	0.9428	0.8285	0.9124	0.9627	1.0367
ACGT-flank	GCAACGTGCC	0.5840	0.4796	0.5496	0.5237	0.6359	0.6833
ACGT-flank	GCAACGTGCG	0.4060	0.7982	0.8159	0.6076	1.0193	0.7180
ACGT-flank	GCAACGTGCT	0.8253	1.1268	0.8448	1.1724	0.8835	0.8126
ACGT-flank	GCAACGTGGA	1.0978	0.8619	1.1053	1.0038	0.9693	1.0486
ACGT-flank	GCAACGTGGC	0.7484	1.2518	0.7742	0.8617	0.9729	0.8423
ACGT-flank	GCAACGTGGG	1.1371	1.1725	1.1378	1.2358	1.0518	1.1910
ACGT-flank	GCAACGTGGT	0.9793	0.8723	0.8571	0.9599	0.9767	0.7875
ACGT-flank	GCAACGTGTA	0.8352	1.0374	0.7336	0.8306	0.7769	0.7829
ACGT-flank	GCAACGTGTC	0.8812	0.9877	0.9559	1.4567	0.9304	1.0005
ACGT-flank	GCAACGTGTG	1.3546	1.0768	1.0207	1.3049	1.1134	1.0747
ACGT-flank	GCAACGTGTT	0.8660	0.6150	0.8605	0.8110	0.8555	0.8612
ACGT-flank	GCAACGTTAG	1.0061	0.7643	0.8775	0.8142	0.7806	0.8299
ACGT-flank	GCAACGTTCA	1.1439	0.6956	1.0093	0.6346	0.8705	0.9262
ACGT-flank	GCAACGTTCC	0.8652	0.9372	0.9670	1.0753	0.9580	1.0628
ACGT-flank	GCAACGTTCG	1.1025	0.8398	1.0994	0.8561	1.1341	0.9796
ACGT-flank	GCAACGTTGA	0.7695	0.8690	0.9185	0.9690	0.9380	0.9036
ACGT-flank	GCAACGTTGC	0.9229	1.0198	1.1819	0.8459	1.0002	0.8159
ACGT-flank	GCAACGTTGG	0.9105	0.9035	0.8074	0.9368	1.0178	0.9753
ACGT-flank	GCAACGTTGT	1.2473	1.1693	1.1766	1.0577	1.1554	1.2417
ACGT-flank	GCAACGTTTG	0.7908	1.4181	0.9239	1.1402	0.7082	0.8589
ACGT-flank	GCCACGTAAT	0.6050	0.9701	1.1622	0.6945	0.7138	0.8396
ACGT-flank	GCCACGTACA	0.8014	1.1336	0.6679	0.8137	0.7795	0.7871
ACGT-flank	GCCACGTACC	0.8131	1.0301	0.8543	0.7982	0.8483	0.9671
ACGT-flank	GCCACGTACG	0.8780	0.6768	0.8612	0.6835	0.7406	0.7479
ACGT-flank	GCCACGTACT	0.8665	0.8330	0.8158	0.8208	0.9873	0.9315
ACGT-flank	GCCACGTAGA	0.7013	0.7178	1.0305	0.4633	0.8200	0.9020
ACGT-flank	GCCACGTAGG	1.1134	0.9968	1.0391	1.0943	1.2233	1.1545

ACGT-flank	GCCACGTAGT	0.8416	1.1100	1.0702	0.9696	1.3098	1.2106
ACGT-flank	GCCACGTATA	0.8900	1.2926	0.8304	1.1776	1.1339	1.0217
ACGT-flank	GCCACGTATC	0.8254	0.9261	0.8522	0.8604	0.7163	0.7646
ACGT-flank	GCCACGTATG	0.9488	1.0803	1.0665	1.0922	1.0010	0.7837
ACGT-flank	GCCACGTCAA	1.0608	0.6537	0.7696	0.6987	0.9208	0.9862
ACGT-flank	GCCACGTCAC	1.0812	1.1492	1.2761	0.8358	1.0034	1.0391
ACGT-flank	GCCACGTCAG	0.8660	0.9229	1.1180	0.9791	0.9687	1.1059
ACGT-flank	GCCACGTCAT	1.0280	0.8587	0.8107	0.7778	0.8348	0.7516
ACGT-flank	GCCACGTCCG	0.9625	0.8393	0.9374	0.7449	0.9989	0.8861
ACGT-flank	GCCACGTCCT	1.1332	0.7669	0.7647	1.0065	0.9172	0.8961
ACGT-flank	GCCACGTCGA	0.8395	0.6743	0.6519	0.6453	0.6883	0.6711
ACGT-flank	GCCACGTCGG	1.1796	1.0220	0.9496	0.8996	1.0146	1.1875
ACGT-flank	GCCACGTCGT	1.1396	1.0347	0.9189	1.0262	0.8779	0.9517
ACGT-flank	GCCACGTCTA	0.5801	0.4462	0.5207	0.4421	0.6252	0.4650
ACGT-flank	GCCACGTCTC	0.7705	0.5073	0.7644	1.2358	0.7411	0.8797
ACGT-flank	GCCACGTCTG	1.2021	1.3081	1.4066	1.3298	1.4903	1.5007
ACGT-flank	GCCACGTCTT	1.2577	1.3129	1.3071	1.4572	1.4483	1.3557
ACGT-flank	GCCACGTGAC	0.6273	0.9353	0.8810	0.8904	0.8743	0.8251
ACGT-flank	GCCACGTGAG	0.8775	0.6881	1.0848	1.0383	0.8485	1.0383
ACGT-flank	GCCACGTGAT	0.9505	1.0175	0.9251	1.0855	0.9442	0.9813
ACGT-flank	GCCACGTGCC	0.9055	1.0991	0.8271	1.0263	1.0548	1.0369
ACGT-flank	GCCACGTGCT	0.9899	0.8351	1.0991	1.1985	0.9178	1.1730
ACGT-flank	GCCACGTGGA	1.2867	1.2957	0.9395	1.2347	1.4782	1.6350
ACGT-flank	GCCACGTGGG	1.1623	1.2653	1.0449	1.1451	1.1909	1.1099
ACGT-flank	GCCACGTGGT	1.0495	0.7958	0.9305	0.9685	1.0321	1.0128
ACGT-flank	GCCACGTGTA	0.9834	0.9380	1.3848	1.3093	1.0706	1.1732
ACGT-flank	GCCACGTGTC	1.2847	1.4968	1.6618	1.5903	1.3881	1.3618
ACGT-flank	GCCACGTGTG	0.9973	0.9299	1.0233	1.0771	1.0819	1.0088
ACGT-flank	GCCACGTGTT	1.3149	1.3840	1.2429	1.4703	1.4853	1.2809
ACGT-flank	GCCACGTTAC	0.8541	0.6664	0.8276	0.8266	0.8892	0.8206
ACGT-flank	GCCACGTTAG	0.7655	0.9331	0.7261	0.9125	0.8722	0.8221
ACGT-flank	GCCACGTTCA	0.9401	1.0205	1.1089	0.6767	1.0452	0.8718
ACGT-flank	GCCACGTTCG	1.0785	1.1564	0.9879	0.8759	0.8639	0.9098
ACGT-flank	GCCACGTTCT	1.3320	1.2918	1.4425	1.2503	1.4553	1.1939
ACGT-flank	GCCACGTTGA	0.9721	0.9366	0.9371	0.9584	0.8838	0.9275
ACGT-flank	GCCACGTTGC	1.0274	0.8826	0.9308	0.7700	0.9508	0.7575
ACGT-flank	GCCACGTTGG	1.1119	1.1759	1.1351	1.2376	1.0771	0.9600
ACGT-flank	GCCACGTTGT	0.8111	0.8177	0.6867	0.7651	0.7047	0.8104
ACGT-flank	GCCACGTTTG	1.1754	1.1298	1.1597	1.0099	1.0865	1.1950
ACGT-flank	GCGACGTAAA	0.9187	0.7858	0.9426	0.8525	0.9663	0.8909
ACGT-flank	GCGACGTAAC	0.8138	0.8253	0.8147	0.7909	0.9071	0.7007
ACGT-flank	GCGACGTAAG	0.7198	0.7874	0.9456	0.8610	0.7514	1.0358
ACGT-flank	GCGACGTAAT	1.1365	1.0298	1.0379	1.0438	0.8913	1.0535
ACGT-flank	GCGACGTACA	0.6514	0.7153	0.5784	0.7473	0.6148	0.7326

ACGT-flank	GCGACGTACC	0.8647	0.7024	0.9224	1.0126	0.9073	0.9311
ACGT-flank	GCGACGTACG	0.8955	0.8489	0.7702	0.8036	0.8887	0.7011
ACGT-flank	GCGACGTACT	1.0414	1.3186	1.0790	1.1316	1.0214	0.9656
ACGT-flank	GCGACGTAGA	0.4440	0.6267	0.3737	0.8999	0.8772	0.8507
ACGT-flank	GCGACGTAGG	1.0408	0.8597	1.0402	1.1161	1.0520	0.9953
ACGT-flank	GCGACGTAGT	0.9132	0.9196	0.9565	0.7454	0.6294	0.7245
ACGT-flank	GCGACGTATA	0.9095	0.8594	0.7304	0.9603	0.8443	0.8500
ACGT-flank	GCGACGTATC	1.1375	1.4926	1.1500	1.1222	1.3711	1.1037
ACGT-flank	GCGACGTATG	0.9632	1.0185	1.0140	0.8904	0.9488	0.9110
ACGT-flank	GCGACGTCAC	0.9325	0.7367	0.6029	0.9831	1.0599	0.8683
ACGT-flank	GCGACGTCAG	0.8598	1.0618	1.0476	0.8636	1.0799	1.0652
ACGT-flank	GCGACGTCAT	1.2574	0.7798	0.9036	0.8762	0.8287	1.0323
ACGT-flank	GCGACGTCCA	1.2619	1.3198	0.9642	1.1835	0.9771	1.0406
ACGT-flank	GCGACGTCCC	0.9674	1.0006	1.1281	1.0858	1.0476	1.0229
ACGT-flank	GCGACGTCCG	1.0220	0.6622	0.7719	0.7040	0.8253	0.8788
ACGT-flank	GCGACGTCCT	0.9217	0.8775	0.9600	1.2378	0.8598	0.8732
ACGT-flank	GCGACGTCGG	0.8994	1.2365	1.0051	0.9971	1.0775	1.0258
ACGT-flank	GCGACGTCGT	0.8364	0.7697	0.8777	0.8570	0.7835	0.9012
ACGT-flank	GCGACGTCTA	0.9266	1.0397	0.4948	0.5203	1.0720	0.6964
ACGT-flank	GCGACGTCTC	0.9714	1.0490	1.2731	1.1605	1.0509	1.1173
ACGT-flank	GCGACGTCTG	0.8490	0.8471	0.7674	0.9489	1.1514	0.9508
ACGT-flank	GCGACGTCTT	0.9765	0.8549	1.0929	1.0491	1.0391	1.0568
ACGT-flank	GCGACGTGAC	0.9247	0.9039	0.7067	1.1167	0.8396	0.8326
ACGT-flank	GCGACGTGAG	0.8480	0.6206	0.8415	1.0064	0.6621	0.7864
ACGT-flank	GCGACGTGAT	0.6826	0.8289	0.7071	0.8239	0.8134	0.8935
ACGT-flank	GCGACGTGCC	0.8107	0.7191	0.8245	0.7843	0.7010	0.8011
ACGT-flank	GCGACGTGCG	0.8973	0.7785	0.7843	0.8761	0.9723	1.0764
ACGT-flank	GCGACGTGGA	1.2774	0.9654	1.1066	1.2791	1.0818	0.9810
ACGT-flank	GCGACGTGGC	0.9334	0.9751	1.1169	0.9690	0.9380	0.9372
ACGT-flank	GCGACGTGGG	1.0635	1.2653	1.3036	1.2747	1.2830	1.3471
ACGT-flank	GCGACGTGGT	1.1936	0.9388	1.1444	0.8655	0.9336	1.0538
ACGT-flank	GCGACGTGTA	1.0056	1.1911	0.8965	0.9202	0.9528	0.9620
ACGT-flank	GCGACGTGTC	0.8053	0.9490	0.9284	0.9040	0.9626	0.9070
ACGT-flank	GCGACGTGTG	1.1246	1.0830	1.0835	1.0996	1.1187	1.0388
ACGT-flank	GCGACGTGTT	1.2599	0.9886	0.8588	1.0790	1.0991	0.9820
ACGT-flank	GCGACGTTAA	1.2635	0.7653	0.9463	0.9926	1.0477	0.9531
ACGT-flank	GCGACGTTAC	0.9965	1.0118	0.9296	0.9070	0.9094	1.0603
ACGT-flank	GCGACGTTAG	0.8071	0.6966	0.7070	0.6539	0.7264	0.7869
ACGT-flank	GCGACGTTAT	0.8093	0.9756	0.4914	0.8697	0.7610	0.6367
ACGT-flank	GCGACGTTCA	0.8554	1.2499	0.6754	1.2452	0.9589	0.9652
ACGT-flank	GCGACGTTCC	0.8611	0.9832	0.8723	0.9560	0.8959	0.9801
ACGT-flank	GCGACGTTCG	0.8565	1.0963	0.7055	0.8722	0.8295	0.7127
ACGT-flank	GCGACGTTCT	1.1584	1.1153	0.9789	1.0647	1.0088	0.9753
ACGT-flank	GCGACGTTGA	0.8464	0.7850	0.8325	1.0643	0.8694	0.8763

ACGT-flank	GCGACGTTGC	1.2324	1.0410	1.0656	1.0331	1.0586	1.0575
ACGT-flank	GCGACGTTGG	0.9717	0.9487	0.9311	0.9074	0.9603	1.0400
ACGT-flank	GCGACGTTGT	0.8908	0.8708	0.9040	0.8835	0.9191	0.8926
ACGT-flank	GCGACGTTTA	1.4485	1.5742	1.3823	1.0136	0.8333	1.1635
ACGT-flank	GCGACGTTTC	0.9597	1.0691	1.0465	0.8620	1.0464	0.9904
ACGT-flank	GCGACGTTTG	1.1652	0.9219	1.1395	1.0833	1.1112	1.0276
ACGT-flank	GCTACGTAAA	0.9815	0.8549	0.8228	0.9691	1.1054	1.1847
ACGT-flank	GCTACGTAAG	0.6714	0.5153	0.6985	0.6186	0.6234	0.5309
ACGT-flank	GCTACGTACA	0.5857	1.0499	1.3337	0.9885	0.9821	1.0298
ACGT-flank	GCTACGTACC	0.7113	0.7232	0.7317	0.7868	0.7155	0.7788
ACGT-flank	GCTACGTACG	0.7135	0.6692	0.8629	0.9184	0.8075	0.7045
ACGT-flank	GCTACGTAGA	0.6444	0.6007	0.6469	0.6083	0.7253	0.7152
ACGT-flank	GCTACGTAGC	0.9849	0.9633	0.8814	0.9087	1.0031	0.9578
ACGT-flank	GCTACGTAGG	1.1080	1.0396	1.0871	0.9425	1.0816	0.9856
ACGT-flank	GCTACGTAGT	1.2030	0.9260	1.1201	1.1457	0.9623	1.2548
ACGT-flank	GCTACGTATC	1.1782	0.9226	0.9612	0.7581	0.9080	0.8731
ACGT-flank	GCTACGTATG	0.7981	1.0055	0.5235	0.6071	0.5500	0.6874
ACGT-flank	GCTACGTCAC	0.6383	0.4173	0.5208	0.5960	0.6040	0.7697
ACGT-flank	GCTACGTCAG	0.8979	0.8482	0.9221	0.8467	0.9240	1.0156
ACGT-flank	GCTACGTCCA	0.8130	0.8811	1.0627	0.8714	0.8729	0.9034
ACGT-flank	GCTACGTCCC	0.7533	0.8352	1.0380	0.9611	0.8671	0.9610
ACGT-flank	GCTACGTCCG	0.8843	0.8883	0.8821	1.1898	1.0015	1.0005
ACGT-flank	GCTACGTCCT	0.9442	0.8550	0.8515	0.9368	0.9641	0.9249
ACGT-flank	GCTACGTCGA	0.8609	0.6667	0.6667	0.9206	0.6749	0.7680
ACGT-flank	GCTACGTCGC	0.8612	0.7625	0.8265	0.9677	0.8539	0.7815
ACGT-flank	GCTACGTCGG	0.6975	0.7916	0.8421	0.7197	0.8472	0.9202
ACGT-flank	GCTACGTCGT	0.9491	0.9566	0.9912	0.9738	0.9179	1.0560
ACGT-flank	GCTACGTCTC	0.7558	0.8658	0.6851	0.8543	0.8699	0.9927
ACGT-flank	GCTACGTCTG	0.7863	0.9788	1.0887	1.0022	1.1147	0.9737
ACGT-flank	GCTACGTGAA	0.6348	0.7513	0.8409	0.7482	0.6160	0.8696
ACGT-flank	GCTACGTGAC	0.6002	0.8623	0.6534	0.9524	0.7345	0.7478
ACGT-flank	GCTACGTGAG	0.9351	0.9448	0.8523	0.8975	0.9816	0.9174
ACGT-flank	GCTACGTGAT	0.7724	0.7479	0.7277	0.5641	0.7929	0.7835
ACGT-flank	GCTACGTGCC	0.5822	0.7089	0.6781	0.7209	0.7157	0.7532
ACGT-flank	GCTACGTGCG	0.7767	0.8246	0.7942	0.9135	0.8599	0.8683
ACGT-flank	GCTACGTGCT	0.6960	0.9183	0.8335	0.7375	0.7820	0.8467
ACGT-flank	GCTACGTGGA	1.1500	1.2252	1.1460	1.2415	1.0829	1.0084
ACGT-flank	GCTACGTGGC	1.1038	0.8622	0.8493	1.2309	1.1800	1.0248
ACGT-flank	GCTACGTGGG	1.4198	1.4198	1.4072	1.4420	1.4265	1.3211
ACGT-flank	GCTACGTGGT	0.9200	1.0627	0.9257	0.9351	1.0146	1.1124
ACGT-flank	GCTACGTGTA	0.9845	1.1849	0.7871	1.2954	1.0416	0.8880
ACGT-flank	GCTACGTGTC	1.0148	1.0661	1.2390	1.2270	1.2225	1.1597
ACGT-flank	GCTACGTGTG	0.9185	0.9321	1.0650	1.0747	1.0204	1.0876
ACGT-flank	GCTACGTTAG	1.1823	1.3294	1.3127	1.4975	1.3104	1.2955

ACGT-flank	GCTACGTTCA	1.2006	1.3457	1.5168	1.0553	1.3345	1.1731
ACGT-flank	GCTACGTTCC	1.1001	0.9335	0.7508	0.8972	0.8917	0.8951
ACGT-flank	GCTACGTTCG	0.8879	1.2087	1.1174	0.9858	1.1148	1.1980
ACGT-flank	GCTACGTTGA	0.3791	1.0915	0.5701	0.6020	0.9474	0.5188
ACGT-flank	GCTACGTTGC	0.7071	0.3569	0.5110	0.6389	0.6804	0.5089
ACGT-flank	GCTACGTTGG	1.0232	0.9523	0.9898	1.0011	1.0016	0.9299
ACGT-flank	GGAACGTAAA	0.8676	0.8090	1.0164	0.9956	1.0048	0.8415
ACGT-flank	GGAACGTAAC	0.6446	0.5646	0.6876	0.6468	0.6329	0.6032
ACGT-flank	GGAACGTAAG	0.8210	0.8181	0.8333	0.8307	0.9366	0.8939
ACGT-flank	GGAACGTAAT	0.8679	0.6562	0.6759	0.6847	0.8153	0.7965
ACGT-flank	GGAACGTACA	1.0353	1.4082	1.0036	0.7904	1.0780	1.0627
ACGT-flank	GGAACGTACC	0.7649	0.8467	0.7268	0.6562	0.7418	0.6866
ACGT-flank	GGAACGTACG	0.7407	0.6918	0.7114	0.7475	0.6989	0.6418
ACGT-flank	GGAACGTACT	1.0749	0.7749	0.7873	0.8628	1.0104	0.9768
ACGT-flank	GGAACGTAGA	1.0235	0.8977	0.9799	1.0407	1.0308	1.0263
ACGT-flank	GGAACGTAGC	0.7340	0.8173	0.7586	0.7763	0.6458	0.7114
ACGT-flank	GGAACGTAGG	1.2099	1.1529	1.4836	1.1478	1.2535	1.3309
ACGT-flank	GGAACGTAGT	0.8503	0.8815	0.8900	0.8785	0.9043	0.9135
ACGT-flank	GGAACGTATA	1.0091	1.0329	0.7569	1.1893	1.0705	0.9733
ACGT-flank	GGAACGTATC	0.9681	1.1287	0.9641	0.9482	0.8503	0.9093
ACGT-flank	GGAACGTATG	1.0074	0.8705	1.0115	0.9756	1.0264	1.0619
ACGT-flank	GGAACGTCAA	0.7621	0.6574	1.0131	0.7835	0.7760	0.8364
ACGT-flank	GGAACGTCAC	0.8323	1.2018	0.9093	1.0758	1.0423	1.0420
ACGT-flank	GGAACGTCAG	0.8691	1.0263	1.0073	0.9850	0.9092	0.9114
ACGT-flank	GGAACGTCAT	0.6378	0.7168	0.7112	0.8547	0.8124	0.7687
ACGT-flank	GGAACGTCCA	0.7882	0.9336	0.6463	0.8647	0.7437	0.6838
ACGT-flank	GGAACGTCCC	0.5495	0.4960	0.5699	0.4891	0.4881	0.6052
ACGT-flank	GGAACGTCCG	1.0836	0.8937	0.9737	0.8000	0.9782	0.9872
ACGT-flank	GGAACGTCCT	0.8585	0.7556	0.4703	0.4705	0.6183	0.6548
ACGT-flank	GGAACGTCGA	0.6591	0.7231	0.7681	0.5507	0.6204	0.6551
ACGT-flank	GGAACGTCGC	0.5471	0.7664	0.7772	1.0327	0.6146	0.7773
ACGT-flank	GGAACGTCGG	0.7352	0.6522	0.7983	0.7570	0.7633	0.7900
ACGT-flank	GGAACGTCGT	0.9036	0.8650	0.8742	0.8732	0.7762	0.8808
ACGT-flank	GGAACGTCTA	0.6604	0.6010	0.6699	0.5296	0.6656	0.6696
ACGT-flank	GGAACGTCTC	0.8260	0.8145	0.8243	0.7741	0.7868	0.9611
ACGT-flank	GGAACGTCTG	0.9925	1.0138	0.9625	0.8884	0.9403	0.8600
ACGT-flank	GGAACGTCTT	1.4462	1.4952	1.0311	0.9937	1.5734	1.3348
ACGT-flank	GGAACGTGAA	0.9611	0.7781	0.9889	0.6790	0.8975	0.9094
ACGT-flank	GGAACGTGAC	0.6644	0.9681	0.7396	0.7819	0.6953	0.6921
ACGT-flank	GGAACGTGAG	0.9660	0.9624	1.2039	0.9774	1.2413	1.1561
ACGT-flank	GGAACGTGAT	0.8855	0.8619	0.8590	0.8490	0.7310	0.8239
ACGT-flank	GGAACGTGCA	0.8066	0.8654	0.9110	0.8148	0.7809	0.9056
ACGT-flank	GGAACGTGCC	1.0670	1.1700	0.9577	0.7576	1.1187	1.1269
ACGT-flank	GGAACGTGCG	1.2392	1.1823	0.9807	0.9136	1.0997	1.0781

ACGT-flank	GGAACGTGCT	1.0882	1.0332	0.9426	0.9378	0.8351	0.9571
ACGT-flank	GGAACGTGGA	1.0233	1.0285	0.9597	1.1062	1.0309	0.9986
ACGT-flank	GGAACGTGGC	0.6488	0.4273	0.3211	0.6565	0.5146	0.5258
ACGT-flank	GGAACGTGGG	1.2350	1.3276	1.4236	1.4301	1.2067	1.2501
ACGT-flank	GGAACGTGGT	0.6489	0.7840	0.6351	0.6946	0.6895	0.7255
ACGT-flank	GGAACGTGTA	1.0376	1.0359	0.9033	1.0398	1.0190	1.0243
ACGT-flank	GGAACGTGTC	0.8512	0.9001	0.8533	0.9377	0.8457	0.8418
ACGT-flank	GGAACGTGTG	0.9879	1.1360	1.1474	1.1605	1.0622	1.0648
ACGT-flank	GGAACGTGTT	0.9012	0.8529	0.8891	1.0318	1.0145	0.9316
ACGT-flank	GGAACGTTAA	0.7262	0.8361	0.5999	0.7852	0.6218	0.6831
ACGT-flank	GGAACGTTAC	0.6487	0.8526	0.5622	0.6313	0.6686	0.6881
ACGT-flank	GGAACGTTAG	0.7977	0.7586	0.8358	0.7143	0.7605	0.7787
ACGT-flank	GGAACGTTCA	0.8704	0.8604	0.6986	0.7851	0.6809	0.7195
ACGT-flank	GGAACGTTCC	0.8818	0.8260	0.8165	0.8339	0.8690	0.8223
ACGT-flank	GGAACGTTCG	0.9497	0.7926	0.8493	0.6841	0.7825	0.7700
ACGT-flank	GGAACGTTCT	0.7242	0.7328	0.6966	0.9985	0.8015	0.7838
ACGT-flank	GGAACGTTGA	0.9050	0.8548	0.9702	0.8917	1.0346	0.8976
ACGT-flank	GGAACGTTGC	0.5945	0.7357	0.8873	0.7854	0.4722	0.6446
ACGT-flank	GGAACGTTGG	0.9701	0.9493	1.0336	0.9109	0.9777	0.9602
ACGT-flank	GGAACGTTGT	0.7355	0.7448	0.8100	0.7718	0.7233	0.7767
ACGT-flank	GGAACGTTTC	0.8520	0.8315	0.8945	0.8542	0.9886	1.0211
ACGT-flank	GGAACGTTTG	0.8591	0.7875	0.8351	0.9581	0.8372	0.8944
ACGT-flank	GGCACGTAAA	0.8316	0.9482	0.5645	1.1214	0.9233	0.9114
ACGT-flank	GGCACGTAAC	0.8767	0.7109	0.8950	0.5039	0.7584	0.7067
ACGT-flank	GGCACGTAAG	0.8782	1.0117	0.8538	0.7883	0.9368	1.0207
ACGT-flank	GGCACGTAAT	0.9687	1.0632	0.9101	0.8056	0.9042	0.9820
ACGT-flank	GGCACGTACA	0.9119	0.6862	0.8849	0.9326	0.8630	0.8602
ACGT-flank	GGCACGTACC	0.7911	0.9342	0.9375	0.9232	0.9339	0.8441
ACGT-flank	GGCACGTACG	0.7379	0.9297	0.8098	0.7631	0.7978	0.8083
ACGT-flank	GGCACGTACT	0.6417	0.8189	0.7844	0.8432	0.9659	0.7309
ACGT-flank	GGCACGTAGA	0.9049	0.9355	0.8906	0.9792	0.8583	0.8977
ACGT-flank	GGCACGTAGC	0.8865	0.6590	1.0058	0.6653	0.9326	0.9935
ACGT-flank	GGCACGTAGG	0.8305	0.9585	0.9362	0.8872	0.9239	0.8505
ACGT-flank	GGCACGTAGT	0.9046	0.8526	0.8650	0.8152	0.8967	0.8786
ACGT-flank	GGCACGTATA	1.4708	0.9705	1.5028	1.2397	1.2464	1.3465
ACGT-flank	GGCACGTATC	0.7676	0.7896	0.8837	1.0711	0.8205	1.1190
ACGT-flank	GGCACGTATG	0.9001	1.0053	0.8378	0.8922	0.8668	0.9423
ACGT-flank	GGCACGTCAA	0.9550	0.9543	0.9620	0.9179	0.9206	0.8504
ACGT-flank	GGCACGTCAC	0.9101	1.3566	0.9510	0.9358	0.6647	0.7976
ACGT-flank	GGCACGTCAG	0.7462	1.0437	1.0609	0.9470	1.0518	1.1079
ACGT-flank	GGCACGTCAT	0.8192	0.8204	1.0156	0.8346	0.9790	0.8940
ACGT-flank	GGCACGTCCA	0.8039	0.6643	0.7564	0.7088	0.8309	0.6405
ACGT-flank	GGCACGTCCG	0.8615	0.7502	0.6490	0.6860	0.7054	0.6607
ACGT-flank	GGCACGTCCT	0.7088	0.7567	0.8151	0.8654	0.8345	0.7975

ACGT-flank	GGCACGTCGA	0.8451	1.1350	0.7535	0.7449	0.9292	0.8729
ACGT-flank	GGCACGTCGC	1.8789	2.1201	2.0311	1.9337	1.7665	1.9687
ACGT-flank	GGCACGTCGG	0.9318	0.7195	0.9642	0.9950	0.9858	0.8211
ACGT-flank	GGCACGTCGT	1.1002	1.0640	1.1523	1.0721	0.9783	0.9744
ACGT-flank	GGCACGTCTA	1.1250	0.8259	1.1030	0.9182	1.1008	1.1625
ACGT-flank	GGCACGTCTC	0.8421	0.8661	0.8210	0.8724	0.7931	0.8384
ACGT-flank	GGCACGTCTG	0.9382	0.7560	0.8842	0.9542	0.8267	0.9278
ACGT-flank	GGCACGTCTT	1.0146	0.8108	0.9956	1.2280	1.0545	1.0956
ACGT-flank	GGCACGTGAA	0.8187	0.7337	0.7903	0.8088	0.9971	0.9596
ACGT-flank	GGCACGTGAC	0.8431	0.9418	1.0081	1.0031	0.9348	0.9227
ACGT-flank	GGCACGTGAG	0.9272	1.4509	0.8349	0.8962	0.9645	0.8936
ACGT-flank	GGCACGTGAT	0.9664	0.8800	1.0416	0.8201	0.9060	0.9201
ACGT-flank	GGCACGTGCA	1.1557	1.2569	1.1424	1.0134	1.0418	1.0620
ACGT-flank	GGCACGTGCC	0.8682	0.8594	0.5542	1.1188	0.9272	1.0371
ACGT-flank	GGCACGTGCG	0.8230	0.9394	0.8961	1.0263	0.9460	0.9878
ACGT-flank	GGCACGTGCT	0.9662	1.0918	1.0369	0.8986	0.9700	0.9626
ACGT-flank	GGCACGTGGA	0.9029	0.9352	1.3481	0.8259	1.1836	1.1566
ACGT-flank	GGCACGTGGC	1.2025	1.1891	1.3984	1.2188	1.1595	1.3331
ACGT-flank	GGCACGTGGG	1.3338	1.3128	1.3172	1.5149	1.4857	1.4225
ACGT-flank	GGCACGTGGT	0.8986	0.8461	1.0056	0.8892	0.9799	0.9766
ACGT-flank	GGCACGTGTA	1.1538	1.2183	1.1403	1.2054	1.2256	1.3316
ACGT-flank	GGCACGTGTC	1.3055	1.2635	1.4001	1.3398	1.5198	1.3633
ACGT-flank	GGCACGTGTG	1.0751	1.0448	1.0761	1.1947	1.1229	1.1021
ACGT-flank	GGCACGTGTT	1.2409	1.2250	1.2547	1.0693	1.2362	1.1970
ACGT-flank	GGCACGTTAA	0.8379	0.9067	1.0051	0.8521	0.8910	0.8176
ACGT-flank	GGCACGTTAC	0.8470	0.8295	1.0150	0.6651	0.8700	0.8343
ACGT-flank	GGCACGTTAG	0.7603	0.9974	0.8957	1.0436	0.8451	0.9144
ACGT-flank	GGCACGTTCA	0.7434	0.8193	0.8247	0.7434	0.7613	0.7223
ACGT-flank	GGCACGTTCC	0.7151	1.0923	0.8370	0.7298	0.9771	1.1273
ACGT-flank	GGCACGTTCG	0.8598	0.7434	0.9098	0.8597	0.9469	0.8516
ACGT-flank	GGCACGTTCT	0.8195	1.1304	1.0059	0.8818	1.0707	0.9494
ACGT-flank	GGCACGTTGA	0.7927	0.7723	1.1445	1.0897	0.9093	0.8211
ACGT-flank	GGCACGTTGC	0.9721	0.9012	1.1178	0.7916	0.7713	0.9527
ACGT-flank	GGCACGTTGG	0.9357	0.9613	0.9864	0.9078	0.9287	0.9211
ACGT-flank	GGCACGTTGT	1.0192	0.8368	1.0271	0.9621	0.9491	1.0563
ACGT-flank	GGCACGTTTC	0.4748	0.6766	0.5074	0.7203	0.5853	0.6183
ACGT-flank	GGCACGTTTG	1.0019	0.9661	0.9495	1.0469	0.9357	0.9156
ACGT-flank	GGGACGTAAA	0.7503	0.7285	0.8005	0.7798	0.7922	0.8354
ACGT-flank	GGGACGTAAC	0.7746	0.7858	0.8478	0.7587	0.7436	0.8576
ACGT-flank	GGGACGTAAG	0.7818	0.8978	0.8657	0.8681	0.8400	0.8654
ACGT-flank	GGGACGTAAT	0.7243	0.8526	0.8605	0.7130	0.6981	0.8052
ACGT-flank	GGGACGTACA	0.8705	0.7202	0.8355	0.6778	0.7337	0.7901
ACGT-flank	GGGACGTACC	0.6234	0.9528	1.0946	1.1837	0.6252	0.8667
ACGT-flank	GGGACGTACG	0.7197	0.8663	0.7916	0.7244	0.7634	0.7207

ACGT-flank	GGGACGTACT	0.6155	0.7242	0.6539	0.7329	0.7374	0.6900
ACGT-flank	GGGACGTAGA	0.7477	0.7292	0.7724	0.7472	0.8032	0.8706
ACGT-flank	GGGACGTAGC	0.7110	0.8177	0.7491	0.6512	0.7324	0.7616
ACGT-flank	GGGACGTAGG	0.9645	1.0657	1.1196	0.8412	1.1869	1.0173
ACGT-flank	GGGACGTAGT	0.9110	0.8241	0.9261	0.8751	0.8533	0.9126
ACGT-flank	GGGACGTATA	1.0922	0.8340	0.9621	0.9588	1.0294	1.1108
ACGT-flank	GGGACGTATC	0.7834	0.7275	0.7232	0.8346	0.7052	0.8194
ACGT-flank	GGGACGTATG	0.8934	0.9342	1.0027	0.9895	0.8586	0.9605
ACGT-flank	GGGACGTATT	0.9827	0.9162	0.8748	1.1678	0.9223	1.1523
ACGT-flank	GGGACGTCAA	0.7371	0.7752	0.8067	0.6607	0.6855	0.7095
ACGT-flank	GGGACGTCAC	0.8207	0.7828	0.8770	0.9076	0.7673	0.7447
ACGT-flank	GGGACGTCAG	0.8242	0.9521	0.8488	0.9470	0.8377	0.9080
ACGT-flank	GGGACGTCAT	0.9873	0.8008	1.0681	1.0326	1.0224	0.9866
ACGT-flank	GGGACGTCCA	1.0118	0.8711	1.0531	0.9100	0.9638	0.9115
ACGT-flank	GGGACGTCCG	0.5931	0.5119	0.8194	0.5187	0.7199	0.6252
ACGT-flank	GGGACGTCCT	0.8185	1.0459	0.6791	0.7451	0.9663	0.7048
ACGT-flank	GGGACGTCGA	0.9783	0.7706	1.1500	0.7578	0.9291	1.0517
ACGT-flank	GGGACGTCGC	0.7966	0.9426	1.0691	1.0395	0.9696	0.8201
ACGT-flank	GGGACGTCGG	0.8368	0.8124	0.7893	0.8808	0.8475	0.8910
ACGT-flank	GGGACGTCGT	0.8594	0.9229	0.9866	0.8905	0.8256	0.8464
ACGT-flank	GGGACGTCTA	0.9162	0.9173	0.9683	0.7518	0.7488	0.7723
ACGT-flank	GGGACGTCTC	0.5663	0.8558	0.6445	0.7054	0.6801	0.6833
ACGT-flank	GGGACGTCTG	0.8469	0.9118	0.8564	0.8112	0.8289	0.8401
ACGT-flank	GGGACGTCTT	1.0861	1.2733	1.3284	1.1130	1.2017	1.2703
ACGT-flank	GGGACGTGAA	0.8877	0.9475	0.8975	0.8703	0.9413	0.8083
ACGT-flank	GGGACGTGAC	1.1589	1.1475	1.3114	0.9798	1.1367	1.0843
ACGT-flank	GGGACGTGAG	0.7571	0.8144	0.7390	0.7029	0.7332	0.7702
ACGT-flank	GGGACGTGAT	0.8596	0.9080	0.8564	0.8256	0.8305	0.8470
ACGT-flank	GGGACGTGCA	0.8279	0.7147	0.7991	0.6230	0.6916	0.7690
ACGT-flank	GGGACGTGCC	0.9092	0.9586	0.9104	0.9011	0.8383	0.8394
ACGT-flank	GGGACGTGCG	0.7702	0.8355	1.0534	0.8548	0.9647	0.9081
ACGT-flank	GGGACGTGCT	0.9198	1.0774	0.8623	0.9341	0.9060	0.8228
ACGT-flank	GGGACGTGGA	0.7666	0.8119	0.9510	0.8353	0.8994	0.8753
ACGT-flank	GGGACGTGGC	0.9511	0.9399	0.8504	0.9744	1.0764	0.9360
ACGT-flank	GGGACGTGGG	1.1856	1.1114	1.2287	1.2644	1.1478	1.1581
ACGT-flank	GGGACGTGGT	0.7594	0.7951	0.8001	0.7442	0.8374	0.8184
ACGT-flank	GGGACGTGTA	0.9205	0.9251	0.9985	0.8491	0.8966	0.8993
ACGT-flank	GGGACGTGTC	0.9588	0.9518	0.8448	0.8865	0.9041	0.9049
ACGT-flank	GGGACGTGTG	1.0100	0.9846	1.1179	1.0807	0.9927	1.0514
ACGT-flank	GGGACGTGTT	1.1393	1.2396	1.1672	1.1729	1.1057	1.0480
ACGT-flank	GGGACGTTAA	0.7444	0.7140	0.7427	0.7546	0.7626	0.7419
ACGT-flank	GGGACGTTAC	0.5696	0.7264	0.5875	0.6052	0.7310	0.6656
ACGT-flank	GGGACGTTAG	0.8543	0.8919	0.8666	0.7750	0.7703	0.7863
ACGT-flank	GGGACGTTAT	0.9792	0.9886	0.6964	1.0211	0.7664	0.8226

ACGT-flank	GGGACGTTCA	1.0823	0.9268	1.0224	0.9008	0.9367	0.9069
ACGT-flank	GGGACGTTCC	0.7630	0.8705	0.9459	0.8221	0.9115	0.8305
ACGT-flank	GGGACGTTCG	0.7708	0.7896	0.7292	0.7774	0.7173	0.7368
ACGT-flank	GGGACGTTCT	0.8199	0.7843	0.6691	0.7937	0.7857	0.8079
ACGT-flank	GGGACGTTGA	0.9356	0.9012	0.8913	0.8645	0.8575	0.9200
ACGT-flank	GGGACGTTGC	0.7716	0.6327	0.6614	0.7183	0.7127	0.7156
ACGT-flank	GGGACGTTGG	0.9824	0.9972	1.0155	0.9536	0.9889	0.9769
ACGT-flank	GGGACGTTGT	0.9373	0.9666	0.9632	0.8369	0.9678	0.9820
ACGT-flank	GGGACGTTTC	0.8002	0.7536	0.8038	0.8224	0.8346	0.8465
ACGT-flank	GGGACGTTTG	0.7985	0.5854	0.7959	0.7537	0.7113	0.6798
ACGT-flank	GGTACGTAAA	0.7951	0.7497	0.9221	0.7885	0.8009	0.9082
ACGT-flank	GGTACGTAAC	0.8393	0.8103	0.6816	0.6486	0.7212	0.6056
ACGT-flank	GGTACGTAAG	0.9181	0.9095	0.9736	0.9230	0.9475	0.9532
ACGT-flank	GGTACGTACA	0.9725	0.9368	0.8935	0.9181	0.8949	0.7890
ACGT-flank	GGTACGTACC	0.7455	0.8346	0.7125	0.8774	0.7960	0.8689
ACGT-flank	GGTACGTACG	0.9894	0.9518	0.9199	1.0679	0.9207	0.9678
ACGT-flank	GGTACGTACT	0.6866	0.8772	1.2019	0.8962	0.8927	0.7245
ACGT-flank	GGTACGTAGA	0.6981	0.8796	0.8035	0.8328	0.8472	1.0106
ACGT-flank	GGTACGTAGC	0.9654	0.7634	0.7756	0.9296	0.8150	0.8450
ACGT-flank	GGTACGTAGG	1.1549	1.0637	1.0399	1.0374	1.0885	1.0747
ACGT-flank	GGTACGTAGT	1.1810	1.2834	1.2631	1.2753	1.2266	1.2128
ACGT-flank	GGTACGTATC	1.4369	1.5830	1.3914	1.1286	1.1168	1.1029
ACGT-flank	GGTACGTATG	25.6910	26.0198	25.2894	25.8442	26.4468	25.4344
ACGT-flank	GGTACGTCAA	0.9200	0.9288	0.9396	0.8874	0.8889	0.8142
ACGT-flank	GGTACGTCAC	1.0242	1.0844	0.7214	0.7738	0.8363	0.8078
ACGT-flank	GGTACGTCAG	0.8909	0.9375	0.8385	0.9348	0.9478	0.8931
ACGT-flank	GGTACGTCCA	0.8973	0.9616	0.8560	0.8867	0.9855	0.9625
ACGT-flank	GGTACGTCCC	0.7077	0.8739	0.7288	0.7375	0.7356	0.8136
ACGT-flank	GGTACGTCCG	0.8039	0.8588	0.7964	0.6429	0.7064	0.8197
ACGT-flank	GGTACGTCCT	0.8591	1.1518	0.9322	0.7109	0.8622	1.0665
ACGT-flank	GGTACGTCGA	0.8138	0.9269	0.8207	0.9039	0.8494	0.8603
ACGT-flank	GGTACGTCGC	0.8249	0.7431	0.8818	0.8077	0.7511	0.8031
ACGT-flank	GGTACGTCGG	1.0115	0.8579	1.0681	0.8642	0.9669	0.8889
ACGT-flank	GGTACGTCGT	0.8912	0.9232	0.9081	0.8374	0.9361	0.9579
ACGT-flank	GGTACGTCTC	1.1003	1.0230	0.9929	0.8162	0.9787	1.0254
ACGT-flank	GGTACGTCTG	0.9551	0.9085	0.9375	0.8471	0.7884	0.7662
ACGT-flank	GGTACGTGAA	0.6238	0.7859	0.7331	0.8325	0.7296	0.7234
ACGT-flank	GGTACGTGAC	0.8969	0.8700	0.8246	0.8593	0.8646	0.9207
ACGT-flank	GGTACGTGAG	0.9586	0.8504	0.8927	0.8190	0.8845	0.8877
ACGT-flank	GGTACGTGAT	1.1314	1.1518	1.0909	0.8879	0.9463	0.9410
ACGT-flank	GGTACGTGCA	0.8817	0.8393	0.8201	0.9546	0.9376	0.8527
ACGT-flank	GGTACGTGCC	0.7810	1.0430	0.9040	0.8457	0.8824	0.8559
ACGT-flank	GGTACGTGCG	0.9550	0.9569	0.9006	0.8986	0.9078	0.9320
ACGT-flank	GGTACGTGCT	1.0254	0.9166	0.8914	0.8292	0.9714	0.8958

ACGT-flank	GGTACGTGGA	0.9559	0.9508	0.9254	1.0495	0.9515	1.0176
ACGT-flank	GGTACGTGGC	1.0765	1.1429	1.2230	1.1348	1.1695	1.1139
ACGT-flank	GGTACGTGGG	1.2888	1.3994	1.3641	1.3969	1.2923	1.3636
ACGT-flank	GGTACGTGGT	0.9296	0.8656	0.9936	0.9532	0.8549	0.9461
ACGT-flank	GGTACGTGTA	1.1581	0.8699	1.1382	0.8574	1.0731	1.1249
ACGT-flank	GGTACGTGTC	1.0601	1.1530	1.1147	1.3585	1.2902	1.2074
ACGT-flank	GGTACGTGTG	1.1881	1.1289	1.1914	1.1846	1.0688	1.1896
ACGT-flank	GGTACGTGTT	1.2761	1.5068	1.2448	1.3044	1.4758	1.4485
ACGT-flank	GGTACGTTAA	0.6974	1.2656	0.9363	0.8780	1.1004	1.1453
ACGT-flank	GGTACGTTAG	0.7237	0.8254	0.8490	0.7220	0.8131	0.7913
ACGT-flank	GGTACGTTCA	1.0872	1.0635	1.0561	0.8998	1.0105	1.1783
ACGT-flank	GGTACGTTCC	1.0006	0.6743	0.9141	0.7350	0.8968	0.7904
ACGT-flank	GGTACGTTCG	0.7000	0.6159	0.7863	0.8622	0.6960	0.7597
ACGT-flank	GGTACGTTGA	0.9352	0.7298	0.8113	0.7555	0.8041	0.7804
ACGT-flank	GGTACGTTGC	0.9444	0.9990	0.9527	0.9349	0.9275	0.8187
ACGT-flank	GGTACGTTGG	0.9056	0.8372	0.8946	0.9262	0.8556	0.9177
ACGT-flank	GGTACGTTGT	1.2036	0.8038	0.9153	0.9770	1.1705	1.0548
ACGT-flank	GTAACGTAAC	0.6027	0.6565	0.4796	0.6302	0.6866	0.6338
ACGT-flank	GTAACGTAAG	0.8435	0.7631	0.6775	0.8412	0.7542	0.8151
ACGT-flank	GTAACGTACC	0.4603	0.6406	0.5198	0.4856	0.5916	0.6836
ACGT-flank	GTAACGTACG	0.8860	0.8515	0.9204	0.8176	0.7719	0.7361
ACGT-flank	GTAACGTAGA	0.9100	0.8586	0.8364	0.7761	0.7966	0.8096
ACGT-flank	GTAACGTAGC	0.7374	0.7838	0.9968	0.7769	0.8056	0.8416
ACGT-flank	GTAACGTAGG	1.0255	0.9192	1.1634	0.8656	1.0231	1.0166
ACGT-flank	GTAACGTCAC	1.3461	0.7835	1.1943	0.9169	0.9946	0.9252
ACGT-flank	GTAACGTCAG	0.9437	0.8576	0.9607	0.9284	0.9561	0.9155
ACGT-flank	GTAACGTCCA	0.6375	0.7404	0.5115	0.7665	0.6131	0.7605
ACGT-flank	GTAACGTCCC	0.8911	0.7391	1.0300	0.9634	1.0299	1.0619
ACGT-flank	GTAACGTCCG	0.9583	0.9028	1.0207	1.0570	0.9220	0.9694
ACGT-flank	GTAACGTCCT	0.7721	0.8804	0.6622	0.9448	0.6574	1.0227
ACGT-flank	GTAACGTCGA	0.9028	0.8172	0.7438	0.7629	0.8038	0.6739
ACGT-flank	GTAACGTCGC	0.9229	0.9559	1.0397	0.8915	0.8476	0.8990
ACGT-flank	GTAACGTCGG	0.8604	1.0295	0.9597	0.8996	0.8975	0.8360
ACGT-flank	GTAACGTCGT	0.9800	1.0331	1.0270	1.1558	0.9054	0.9977
ACGT-flank	GTAACGTCTG	1.0861	0.8373	0.7934	0.9471	0.9563	1.1336
ACGT-flank	GTAACGTGAA	0.8908	0.9857	0.8864	0.7317	0.6862	0.7966
ACGT-flank	GTAACGTGAC	0.5675	0.3029	0.6302	0.4380	0.8253	0.8000
ACGT-flank	GTAACGTGAG	0.8395	0.9159	1.0531	0.9245	0.8750	0.8702
ACGT-flank	GTAACGTGCA	0.6124	0.6326	0.6073	0.6965	0.6439	0.7534
ACGT-flank	GTAACGTGCC	0.8051	1.2045	0.9482	0.9124	0.7644	0.9164
ACGT-flank	GTAACGTGCG	1.1120	0.9822	0.9350	0.9501	0.9505	0.9499
ACGT-flank	GTAACGTGCT	0.8818	0.8010	0.7248	0.6346	0.8218	0.8623
ACGT-flank	GTAACGTGGA	0.8178	1.0027	0.8651	1.0024	0.7269	0.8790
ACGT-flank	GTAACGTGGC	0.9111	0.9319	1.0154	1.0116	0.9224	0.9288

ACGT-flank	GTAACGTGGG	1.1728	1.0831	1.0711	1.0901	1.1404	1.1657
ACGT-flank	GTAACGTGGT	0.8106	0.8416	0.8193	0.9170	0.8335	0.8711
ACGT-flank	GTAACGTGTA	1.2552	1.1026	1.1194	1.0928	0.9619	1.1384
ACGT-flank	GTAACGTGTC	1.1681	0.9973	1.2339	1.1359	1.1159	1.2238
ACGT-flank	GTAACGTGTG	1.2060	1.1090	1.1532	1.1344	1.0958	1.1587
ACGT-flank	GTAACGTTCG	0.7391	0.6635	0.8043	0.9063	0.6711	0.8448
ACGT-flank	GTAACGTTGC	1.0290	0.9420	0.8996	1.0181	0.8042	1.0568
ACGT-flank	GTAACGTTGG	1.2374	0.9714	1.1349	1.1242	1.0390	1.0308
ACGT-flank	GTCACGTAAA	0.8843	0.9812	1.0012	0.8621	0.7240	0.8110
ACGT-flank	GTCACGTAAC	0.9639	0.8643	0.9242	0.9739	0.8105	1.0903
ACGT-flank	GTCACGTAAG	0.6233	0.8846	0.5591	0.9004	0.7264	0.7710
ACGT-flank	GTCACGTACA	0.9517	0.8964	0.9619	0.7598	0.8594	0.9246
ACGT-flank	GTCACGTACC	0.4134	0.9741	0.6025	0.6613	0.7393	0.8477
ACGT-flank	GTCACGTACG	0.8057	0.7318	0.7643	0.8474	0.7653	0.7480
ACGT-flank	GTCACGTAGA	0.9143	1.1062	1.3375	0.9111	0.9996	0.8733
ACGT-flank	GTCACGTAGC	0.9060	1.1806	0.8455	0.8705	0.9652	1.0239
ACGT-flank	GTCACGTAGG	1.0471	1.1108	1.0962	0.9315	1.0676	1.0098
ACGT-flank	GTCACGTATG	0.8409	0.8455	0.8081	0.8553	0.8089	0.9215
ACGT-flank	GTCACGTCAA	0.9796	0.5385	0.5433	0.7554	0.7772	1.0269
ACGT-flank	GTCACGTCAC	0.9840	0.8980	0.3827	0.7983	0.6188	0.9732
ACGT-flank	GTCACGTCAG	1.1104	1.0370	0.9812	1.2186	1.0522	1.0424
ACGT-flank	GTCACGTCCA	0.9402	0.9771	0.7085	0.9960	0.8992	0.7959
ACGT-flank	GTCACGTCCC	1.1829	1.0423	1.2673	1.1153	1.1197	1.1829
ACGT-flank	GTCACGTCCG	1.1432	0.8172	0.9684	1.0025	0.9777	0.9713
ACGT-flank	GTCACGTCCT	0.8283	0.5358	0.7816	0.8570	0.7346	0.8425
ACGT-flank	GTCACGTCGA	1.0193	1.1287	0.8762	0.8829	0.9234	1.0426
ACGT-flank	GTCACGTCGC	0.7499	0.7894	0.7787	0.6779	0.7594	0.8183
ACGT-flank	GTCACGTCGG	0.8013	0.8542	0.8950	0.9214	0.8991	0.8062
ACGT-flank	GTCACGTCGT	0.6051	0.7387	0.7143	0.7559	0.7600	0.8691
ACGT-flank	GTCACGTCTG	0.8544	0.9738	0.9198	0.9848	0.9597	0.8862
ACGT-flank	GTCACGTGAA	1.0099	1.0146	0.6870	0.8962	0.8525	0.7990
ACGT-flank	GTCACGTGAC	0.7890	0.8809	0.9054	0.8394	0.9101	0.9374
ACGT-flank	GTCACGTGAG	1.0298	1.0328	1.1014	0.8132	0.9028	0.9652
ACGT-flank	GTCACGTGAT	0.9269	0.8324	0.9660	0.9216	0.7941	0.8113
ACGT-flank	GTCACGTGCA	0.7560	1.1319	1.2231	0.8483	0.7559	1.0546
ACGT-flank	GTCACGTGCC	1.1493	0.8318	1.0670	1.0263	0.9159	0.9718
ACGT-flank	GTCACGTGCG	1.0037	1.1238	0.9277	0.9287	1.0000	0.9415
ACGT-flank	GTCACGTGCT	1.1322	1.2676	1.1152	1.0138	1.0608	1.1400
ACGT-flank	GTCACGTGGA	1.0834	0.7517	0.9246	0.8302	0.8810	0.8500
ACGT-flank	GTCACGTGGC	0.9856	0.9710	1.0443	0.9759	0.8255	0.9338
ACGT-flank	GTCACGTGGG	1.3612	1.3514	1.4103	1.4664	1.3702	1.4743
ACGT-flank	GTCACGTGGT	0.8395	1.0146	0.8898	0.9523	0.9103	0.8860
ACGT-flank	GTCACGTGTA	1.1367	1.2682	1.1913	1.0379	1.1361	1.2145
ACGT-flank	GTCACGTGTC	0.7730	0.8570	0.9050	0.8937	0.9065	0.9122

ACGT-flank	GTCACGTGTG	1.4280	1.3633	1.3983	1.2983	1.4070	1.3922
ACGT-flank	GTCACGTTCC	1.1063	1.0729	1.2136	0.8279	1.0988	0.9392
ACGT-flank	GTCACGTTCG	0.9195	0.9649	0.9513	0.7782	0.8685	0.9511
ACGT-flank	GTCACGTTGA	0.7563	0.6242	1.2262	0.9775	0.8640	0.9056
ACGT-flank	GTCACGTTGC	0.9606	0.8619	1.1212	1.0777	1.0172	0.8360
ACGT-flank	GTCACGTTGG	0.9941	0.9223	0.8245	0.9726	0.8748	0.8294
ACGT-flank	GTCACGTTGT	0.8512	0.8985	0.9380	0.7804	0.7624	0.8245
ACGT-flank	GTGACGTAAA	0.9444	1.1143	0.8894	0.7719	0.8400	1.1145
ACGT-flank	GTGACGTAAC	0.8906	0.8889	0.9259	0.8628	1.0037	1.0261
ACGT-flank	GTGACGTAAG	0.8509	0.7850	1.1297	0.8277	0.7736	0.9550
ACGT-flank	GTGACGTACA	1.0341	0.9895	0.9530	0.7049	0.7812	0.8799
ACGT-flank	GTGACGTACC	0.9129	0.8022	0.8860	0.8927	0.8988	0.9044
ACGT-flank	GTGACGTACG	0.6584	0.7420	0.8530	0.6632	0.7560	0.6927
ACGT-flank	GTGACGTACT	0.7335	0.8326	0.7476	0.8272	0.7713	0.9380
ACGT-flank	GTGACGTAGA	0.8943	0.8106	0.9421	0.8632	1.0061	0.9096
ACGT-flank	GTGACGTAGC	0.6351	0.6035	0.5379	0.7839	0.6035	0.7232
ACGT-flank	GTGACGTAGG	1.0811	0.9578	1.1101	0.9207	1.0902	1.0352
ACGT-flank	GTGACGTAGT	0.9154	0.7577	0.7880	0.9580	0.9822	0.9133
ACGT-flank	GTGACGTATC	0.8490	1.0502	0.9167	0.9762	0.8815	0.8018
ACGT-flank	GTGACGTATG	0.7580	0.7516	0.8629	0.7768	0.6284	0.7456
ACGT-flank	GTGACGTCAA	1.3576	1.6011	1.1637	1.1694	1.1739	0.9564
ACGT-flank	GTGACGTCAC	1.0254	1.0258	0.7843	0.8467	0.8783	0.8430
ACGT-flank	GTGACGTCAG	0.7846	0.7631	0.7594	0.9131	0.9096	0.8907
ACGT-flank	GTGACGTCAT	0.9480	1.0323	0.7396	0.8546	0.8679	0.9055
ACGT-flank	GTGACGTCCA	0.8046	1.0834	0.9959	0.8321	1.0463	0.9385
ACGT-flank	GTGACGTCCC	0.8812	0.8453	0.7340	0.8512	0.8541	0.8439
ACGT-flank	GTGACGTCCG	0.9338	0.9786	0.7996	0.7668	0.8037	0.8502
ACGT-flank	GTGACGTCCT	0.8244	1.0173	0.7513	0.8845	0.7089	0.9318
ACGT-flank	GTGACGTCGA	0.9753	0.7634	0.7807	0.8874	0.8348	0.8273
ACGT-flank	GTGACGTCGC	0.5887	0.6766	0.5067	0.6131	0.6765	0.6393
ACGT-flank	GTGACGTCGG	0.8357	0.8952	0.9680	0.8591	0.8397	0.9244
ACGT-flank	GTGACGTCGT	0.9147	1.0948	1.0385	0.9443	0.8778	0.9215
ACGT-flank	GTGACGTCTA	1.1171	0.7862	1.3413	0.9746	1.4247	1.0349
ACGT-flank	GTGACGTCTC	0.7517	1.0240	0.8402	0.8680	0.7735	0.6731
ACGT-flank	GTGACGTCTG	0.8066	0.7959	0.7784	0.8340	0.8860	0.8883
ACGT-flank	GTGACGTCTT	1.0323	1.4331	1.1610	1.1531	1.1015	1.1372
ACGT-flank	GTGACGTGAA	1.1298	0.7866	1.3199	1.0112	0.9609	1.0517
ACGT-flank	GTGACGTGAC	0.8866	0.9156	0.9239	1.1808	0.9873	0.8337
ACGT-flank	GTGACGTGAG	0.7742	0.9203	0.7767	0.7649	0.8319	0.9206
ACGT-flank	GTGACGTGAT	0.9036	0.9195	0.8349	0.8918	0.7787	0.7453
ACGT-flank	GTGACGTGCA	0.7656	0.8658	0.6706	0.7798	0.6017	0.6962
ACGT-flank	GTGACGTGCC	0.8397	0.6644	0.7824	0.7690	0.7067	0.7293
ACGT-flank	GTGACGTGCG	1.0213	0.9359	1.1483	1.0466	0.8566	0.8956
ACGT-flank	GTGACGTGCT	0.7983	0.7982	0.8211	0.8206	0.8736	0.7791

ACGT-flank	GTGACGTGGA	1.2431	0.9919	1.1416	1.0388	1.1706	1.2553
ACGT-flank	GTGACGTGGC	1.3707	1.1005	1.2976	1.4060	1.2374	1.1051
ACGT-flank	GTGACGTGGG	1.1055	1.1426	1.0192	1.2215	1.1153	1.0614
ACGT-flank	GTGACGTGGT	0.9845	0.9303	1.0223	0.8988	0.8507	0.9170
ACGT-flank	GTGACGTGTA	0.8309	1.1071	0.9757	1.0140	1.0325	0.8343
ACGT-flank	GTGACGTGTC	1.1183	1.2190	1.0083	1.0166	1.2041	1.2559
ACGT-flank	GTGACGTGTG	1.2276	1.0050	1.2058	1.3377	1.1363	1.2257
ACGT-flank	GTGACGTGTT	0.8360	0.9307	1.1325	1.2201	0.9899	1.1035
ACGT-flank	GTGACGTTAC	0.5626	0.7153	0.6079	0.4801	0.8153	0.5834
ACGT-flank	GTGACGTTAG	1.1861	1.0289	1.0704	0.8819	0.9945	0.9560
ACGT-flank	GTGACGTTCA	0.9999	0.8805	0.9522	1.2560	1.1233	1.0256
ACGT-flank	GTGACGTTCC	1.0389	0.9333	0.8472	0.8017	0.9624	0.8511
ACGT-flank	GTGACGTTCG	0.8158	0.7849	0.7761	0.7914	0.7918	0.8610
ACGT-flank	GTGACGTTGA	1.0339	0.9798	1.0387	1.0625	1.0517	0.9382
ACGT-flank	GTGACGTTGC	0.6745	0.7733	0.8938	0.8338	0.7527	0.6953
ACGT-flank	GTGACGTTGG	0.8429	0.8923	0.8580	0.8737	0.8481	0.8247
ACGT-flank	GTGACGTTGT	0.9686	0.7775	0.9027	0.7438	0.8540	0.8428
ACGT-flank	GTGACGTTTG	0.8902	0.7635	0.7895	0.8388	0.7986	0.9074
ACGT-flank	GTTACGTAGA	0.9542	0.9238	1.0345	0.8128	1.0010	0.9723
ACGT-flank	GTTACGTAGC	1.1147	1.0475	0.9829	0.9673	1.1007	0.9736
ACGT-flank	GTTACGTAGG	1.0276	1.2395	1.1739	1.1282	1.1515	1.1290
ACGT-flank	GTTACGTCCC	0.8388	0.6556	0.5364	0.7897	0.7418	0.9234
ACGT-flank	GTTACGTCCG	1.2899	1.4414	1.3468	1.4028	1.1982	1.2336
ACGT-flank	GTTACGTCGA	0.8641	0.7399	0.8176	0.7428	0.8241	0.8070
ACGT-flank	GTTACGTCGC	0.5197	0.8024	0.5788	0.6432	0.5744	0.7681
ACGT-flank	GTTACGTCGG	0.9834	0.9227	0.8127	0.8969	0.9420	0.9987
ACGT-flank	GTTACGTGAC	0.8292	0.6051	0.7274	0.8495	0.7051	0.6695
ACGT-flank	GTTACGTGAG	0.6928	0.7381	0.7774	0.6233	0.7356	0.7963
ACGT-flank	GTTACGTGCA	1.0526	1.1530	1.0943	1.1559	0.9955	1.0659
ACGT-flank	GTTACGTGCC	0.8440	0.7363	0.5794	0.6597	0.7702	0.7279
ACGT-flank	GTTACGTGCG	1.0464	0.8642	1.0312	1.0848	0.9489	1.1041
ACGT-flank	GTTACGTGGA	1.0245	1.0716	0.8148	0.9084	0.9952	1.0464
ACGT-flank	GTTACGTGGC	0.7905	0.8165	0.8446	0.8342	1.0213	0.8676
ACGT-flank	GTTACGTGGG	1.2079	1.1995	1.2022	1.2133	1.2102	1.1547
ACGT-flank	GTTACGTGGT	0.7820	0.8474	0.8181	0.7571	0.6635	0.6419
ACGT-flank	GTTACGTGTG	1.4105	0.9794	1.0464	0.8692	0.9427	1.2518
ACGT-flank	TAAACGTAAA	0.9947	0.7197	1.2163	1.1958	0.8762	1.1455
ACGT-flank	TAAACGTAAG	1.1968	1.6323	1.1454	1.2863	1.5980	1.7764
ACGT-flank	TAAACGTACC	1.3855	1.2771	1.3158	1.8275	1.0907	1.2387
ACGT-flank	TAAACGTACG	0.9956	0.5595	0.8379	1.1575	0.9430	1.0416
ACGT-flank	TAAACGTAGA	0.7406	0.9233	0.8527	0.9361	0.9033	0.9066
ACGT-flank	TAAACGTAGC	0.6523	0.8281	0.9502	0.8408	0.8510	0.5812
ACGT-flank	TAAACGTAGG	1.1616	1.1275	0.9286	1.1750	0.9638	1.0332
ACGT-flank	TAAACGTCAG	1.1385	1.0655	1.1249	0.9422	1.0917	1.1570

ACGT-flank	TAAACGTCCC	0.7180	0.6919	0.6311	0.7664	0.6603	0.6092
ACGT-flank	TAAACGTCCG	0.7188	0.7022	0.9900	0.6350	0.6752	0.8082
ACGT-flank	TAAACGTCGC	1.2406	0.9113	1.2223	0.8493	0.8840	1.0232
ACGT-flank	TAAACGTCGG	0.9288	1.0141	0.8483	0.7734	0.9264	0.9204
ACGT-flank	TAAACGTGAA	0.3550	0.2989	0.5398	0.7425	0.5929	0.4597
ACGT-flank	TAAACGTGAC	0.8625	0.8143	0.7749	0.9476	0.8012	0.7246
ACGT-flank	TAAACGTGAG	1.1854	0.9112	1.1061	0.9355	1.0976	1.0421
ACGT-flank	TAAACGTGCC	0.8467	0.8354	0.7349	0.7540	0.7164	0.6862
ACGT-flank	TAAACGTGCG	0.9907	0.8343	0.6553	0.9477	0.8091	0.7309
ACGT-flank	TAAACGTGCT	0.5410	0.6424	0.4886	0.9446	0.8401	0.7834
ACGT-flank	TAAACGTGGA	0.7915	1.0838	0.7902	0.8308	1.1422	1.0915
ACGT-flank	TAAACGTGGC	0.8843	1.0008	1.0478	0.7243	0.8402	0.9087
ACGT-flank	TAAACGTGGG	1.5533	1.4571	1.4542	1.6808	1.5160	1.4958
ACGT-flank	TAAACGTGTG	1.3647	1.7893	1.2907	1.6428	1.6010	1.7806
ACGT-flank	TAAACGTTGG	1.0257	0.9959	1.0118	1.4564	1.0402	0.9433
ACGT-flank	TACACGTACG	1.1180	0.8438	0.7257	0.9497	0.9154	0.8660
ACGT-flank	TACACGTAGA	0.8822	0.9470	1.0808	1.0803	1.0271	1.0099
ACGT-flank	TACACGTAGG	0.9302	1.0289	0.9580	0.9808	0.9455	0.8204
ACGT-flank	TACACGTCAG	0.8870	0.8737	0.6730	0.8876	0.6848	0.8223
ACGT-flank	TACACGTCCA	0.8287	0.8367	0.6575	0.6958	0.6453	0.7944
ACGT-flank	TACACGTCCG	0.8656	0.9824	0.9155	0.9172	0.8284	0.6125
ACGT-flank	TACACGTCGA	0.8526	0.5100	0.6933	0.2908	0.4604	0.4984
ACGT-flank	TACACGTCGC	0.9061	0.8557	0.9825	1.1016	0.8223	0.8613
ACGT-flank	TACACGTCGG	1.1162	1.0941	0.8966	0.8881	0.9115	0.9650
ACGT-flank	TACACGTGAG	0.6656	0.4423	0.2220	0.4899	0.5370	0.6999
ACGT-flank	TACACGTGCC	1.1119	1.1403	1.0828	1.1055	1.1217	1.2182
ACGT-flank	TACACGTGCG	1.0656	1.1052	1.0598	1.1907	1.1824	1.1587
ACGT-flank	TACACGTGGA	1.8860	1.5851	2.0107	2.3098	1.9865	1.9810
ACGT-flank	TACACGTGGC	1.9332	2.3896	2.3648	2.8444	2.7545	2.7070
ACGT-flank	TACACGTGGG	1.6192	1.3362	1.4993	1.6373	1.5875	1.5718
ACGT-flank	TACACGTGGT	1.3208	1.4508	1.5389	1.6614	1.5542	1.4986
ACGT-flank	TACACGTGTG	1.5098	1.6747	1.6803	1.6962	1.4798	1.7556
ACGT-flank	TACACGTTGG	0.7396	0.6604	0.8661	0.6987	0.7668	0.9940
ACGT-flank	TAGACGTAAC	0.5621	0.7905	0.6331	0.4810	0.4902	0.7786
ACGT-flank	TAGACGTAAG	0.9745	0.9079	1.0358	1.1394	0.9535	1.0709
ACGT-flank	TAGACGTACA	0.6650	0.5902	0.5649	0.1277	0.4644	0.6739
ACGT-flank	TAGACGTACC	0.8896	0.9791	1.0158	0.8411	1.0464	1.0329
ACGT-flank	TAGACGTACG	0.5397	0.7516	0.4707	0.6970	0.4884	0.5085
ACGT-flank	TAGACGTACT	0.7791	0.5638	0.8036	0.8238	0.9064	1.0606
ACGT-flank	TAGACGTAGA	0.8792	0.7342	0.9729	0.9158	0.7717	0.9074
ACGT-flank	TAGACGTAGC	0.6872	0.8905	0.7373	0.6255	0.6599	0.6696
ACGT-flank	TAGACGTAGG	1.1702	1.2068	1.2506	1.2472	1.2505	1.2480
ACGT-flank	TAGACGTAGT	1.2483	0.9559	1.0345	0.8597	1.2020	1.2471
ACGT-flank	TAGACGTATG	1.0380	0.9512	1.0368	0.9129	1.1506	1.0116

ACGT-flank	TAGACGTCAA	0.8697	1.0571	0.8605	0.9824	0.9823	0.9825
ACGT-flank	TAGACGTCAC	0.8183	0.7627	0.7997	0.4936	0.7370	0.6474
ACGT-flank	TAGACGTCAG	0.8549	0.8795	0.9488	0.7426	0.8525	0.7879
ACGT-flank	TAGACGTCCA	0.9682	0.9344	0.7961	0.6010	0.9198	0.8551
ACGT-flank	TAGACGTCCC	0.9136	0.7920	0.6021	0.7762	0.8455	0.7090
ACGT-flank	TAGACGTCCG	0.4001	0.8759	0.6150	0.8034	0.6546	0.7003
ACGT-flank	TAGACGTCGA	0.7422	0.9068	0.8416	0.8968	0.7991	0.8250
ACGT-flank	TAGACGTCGC	1.0174	1.1939	1.2400	1.0273	1.1790	1.1299
ACGT-flank	TAGACGTCGG	0.9138	0.9832	0.7384	0.8748	0.8835	0.9428
ACGT-flank	TAGACGTCGT	0.7009	0.8631	0.9728	0.8036	0.6800	0.7821
ACGT-flank	TAGACGTCTC	0.7841	0.7680	0.7813	0.7689	0.8032	0.9407
ACGT-flank	TAGACGTCTG	1.5907	1.2330	1.1631	1.1505	1.0524	1.0576
ACGT-flank	TAGACGTGAA	0.7721	0.8361	0.6351	0.8389	0.8164	0.7040
ACGT-flank	TAGACGTGAC	0.8619	0.8941	0.5524	0.6894	0.7527	0.7727
ACGT-flank	TAGACGTGAG	0.8866	0.9120	1.2279	1.1030	1.0620	1.0993
ACGT-flank	TAGACGTGCA	0.9768	0.6618	0.9276	0.6293	0.8679	0.8613
ACGT-flank	TAGACGTGCC	0.6571	0.7697	0.6643	1.0382	0.5869	0.6537
ACGT-flank	TAGACGTGCG	0.7780	1.0054	0.7078	0.8926	0.8790	0.8377
ACGT-flank	TAGACGTGCT	1.2362	1.0824	1.0316	1.2561	1.0451	0.9329
ACGT-flank	TAGACGTGGA	0.6809	0.9197	0.7183	0.8154	0.8033	0.7654
ACGT-flank	TAGACGTGGC	1.1108	0.9291	0.9378	1.0666	1.0193	1.0941
ACGT-flank	TAGACGTGGG	1.3429	1.3097	1.1983	1.3709	1.2872	1.3156
ACGT-flank	TAGACGTGGT	0.9754	0.9054	0.9125	0.8935	0.9142	0.9908
ACGT-flank	TAGACGTGTA	1.3734	1.0870	0.8786	1.0221	0.9165	1.0106
ACGT-flank	TAGACGTGTC	0.8101	1.0185	0.8428	1.0101	1.0300	0.9580
ACGT-flank	TAGACGTGTG	1.3106	1.2189	1.3754	1.5222	1.3277	1.4263
ACGT-flank	TAGACGTTAG	0.9060	0.9831	0.9955	0.9550	1.1415	0.9411
ACGT-flank	TAGACGTTCG	0.9291	0.9805	0.7248	0.7888	0.7534	0.7713
ACGT-flank	TAGACGTTGA	0.7898	0.9188	0.8614	0.5338	0.7337	0.8975
ACGT-flank	TAGACGTTGC	0.7495	0.8602	0.8334	0.8427	0.8855	0.6602
ACGT-flank	TAGACGTTGG	1.1607	1.0526	0.9972	1.0520	0.9528	1.1142
ACGT-flank	TATACGTCGG	0.8146	0.8364	0.7574	0.6829	0.6697	0.7982
ACGT-flank	TATACGTGAG	0.6672	0.9865	0.7220	0.6739	0.7685	0.8932
ACGT-flank	TATACGTGGC	1.1101	1.2859	1.2781	1.5023	1.2607	1.2225
ACGT-flank	TATACGTGGG	1.7565	1.6985	1.6864	1.9932	1.7343	1.5569
ACGT-flank	TCAACGTAAG	1.5317	2.2897	1.8396	1.7042	1.5996	1.5657
ACGT-flank	TCAACGTACG	1.4115	1.5310	1.1195	1.5742	1.4120	1.3084
ACGT-flank	TCAACGTAGA	0.5849	0.7352	0.8493	2.0586	0.7262	1.0843
ACGT-flank	TCAACGTAGC	1.3980	1.5294	1.7332	1.8932	1.5399	1.8262
ACGT-flank	TCAACGTAGG	2.2058	1.9550	2.3284	2.8043	2.3966	2.1240
ACGT-flank	TCAACGTATG	2.2121	1.6703	1.6481	2.4185	1.7770	1.9920
ACGT-flank	TCAACGTCAG	1.1491	1.4762	1.3101	1.3030	1.4644	1.0778
ACGT-flank	TCAACGTCCA	1.2711	1.1791	1.2746	1.1350	1.4190	1.3527
ACGT-flank	TCAACGTCCC	1.0897	0.7508	0.9883	1.0885	1.1032	0.9030

ACGT-flank	TCAACGTCCG	1.2843	1.3085	1.3299	1.2758	1.1489	1.0507
ACGT-flank	TCAACGTCGA	1.1531	1.0887	0.9514	0.9616	1.2386	1.2985
ACGT-flank	TCAACGTCGC	1.0986	0.9957	1.1816	1.3398	1.2837	1.0967
ACGT-flank	TCAACGTCGG	1.8220	1.8476	1.5261	2.1470	1.9072	1.8059
ACGT-flank	TCAACGTCGT	0.8778	0.9717	0.9413	1.1956	0.8783	0.9397
ACGT-flank	TCAACGTCTG	1.8879	1.4329	1.5190	1.7781	1.4262	1.7189
ACGT-flank	TCAACGTGAC	1.3325	1.0852	1.3115	1.1502	1.1707	1.2848
ACGT-flank	TCAACGTGAG	1.3575	1.2331	1.1561	1.9373	1.4695	1.4783
ACGT-flank	TCAACGTGCA	1.3169	1.1002	1.4174	1.0435	1.3813	1.2502
ACGT-flank	TCAACGTGCC	1.2057	1.1471	1.3396	1.1708	1.2913	1.2221
ACGT-flank	TCAACGTGCG	1.2560	1.3375	1.2881	1.6368	1.4530	1.3756
ACGT-flank	TCAACGTGGA	1.4845	1.4313	1.3622	1.2801	1.3630	1.2791
ACGT-flank	TCAACGTGGC	1.4601	1.1580	1.4519	1.6048	1.5395	1.3573
ACGT-flank	TCAACGTGGG	2.5078	2.4444	2.5921	2.9869	2.6223	2.4618
ACGT-flank	TCAACGTGGT	1.3046	1.2707	0.9179	1.2329	1.0892	0.9939
ACGT-flank	TCAACGTGTG	2.6331	2.6452	2.6012	2.8608	2.7075	2.4339
ACGT-flank	TCAACGTTGC	1.2741	1.4469	1.2035	2.3324	1.5299	1.5041
ACGT-flank	TCAACGTTGG	2.3216	2.4245	2.3577	3.0604	2.4783	2.1900
ACGT-flank	TCCACGTAAG	0.7865	0.8489	0.7051	0.8007	0.8407	0.7720
ACGT-flank	TCCACGTACC	0.8549	1.1320	1.0478	0.7340	0.8768	0.9884
ACGT-flank	TCCACGTACG	0.7449	0.8332	0.9215	0.9176	0.9059	0.9026
ACGT-flank	TCCACGTAGA	1.0656	0.6669	0.8774	0.8878	1.1004	1.0299
ACGT-flank	TCCACGTAGC	1.1102	0.8062	1.0752	0.8964	1.3799	1.2360
ACGT-flank	TCCACGTAGG	1.2962	1.2177	1.2139	1.2732	1.1699	1.2957
ACGT-flank	TCCACGTAGT	0.8243	0.7923	0.7988	0.8526	1.2614	0.8397
ACGT-flank	TCCACGTCAC	0.7961	1.1133	1.3277	0.9561	0.9932	0.9802
ACGT-flank	TCCACGTCAG	0.7566	0.5362	0.9565	0.9356	0.6087	0.5230
ACGT-flank	TCCACGTCCA	1.3243	1.2436	1.3968	1.1213	1.3806	1.4788
ACGT-flank	TCCACGTCCC	0.9644	1.0043	0.9764	0.9385	0.9116	0.9544
ACGT-flank	TCCACGTCCG	0.8773	0.9386	0.8128	0.9231	1.0700	0.9144
ACGT-flank	TCCACGTCCT	1.0857	1.1419	0.9095	0.9208	0.9023	1.0170
ACGT-flank	TCCACGTCGA	1.3928	1.2558	1.2979	1.2604	1.3255	1.1424
ACGT-flank	TCCACGTCGC	0.8667	1.1335	0.9752	0.9195	0.9792	0.9346
ACGT-flank	TCCACGTCGG	0.9850	1.2167	1.1000	1.1790	1.0850	1.0839
ACGT-flank	TCCACGTCGT	0.9180	0.6456	0.8168	1.0462	0.7908	0.8910
ACGT-flank	TCCACGTCTC	0.8114	0.9100	0.6341	1.2103	0.7555	0.7004
ACGT-flank	TCCACGTCTG	0.9620	0.9074	0.9392	1.1048	0.9685	1.1156
ACGT-flank	TCCACGTGAA	0.9318	0.8455	0.7479	1.0229	0.8244	0.8559
ACGT-flank	TCCACGTGAC	0.7082	0.4746	0.7796	0.9305	0.9111	0.9102
ACGT-flank	TCCACGTGAG	1.0693	0.9442	1.0694	1.0867	1.0056	1.0202
ACGT-flank	TCCACGTGCA	1.0387	0.8789	0.8969	1.0337	1.0113	1.1015
ACGT-flank	TCCACGTGCC	1.3566	1.2710	1.2569	1.5398	1.3719	1.4463
ACGT-flank	TCCACGTGCG	0.9843	0.8795	1.0256	1.1049	0.9698	1.0367
ACGT-flank	TCCACGTGCT	1.1169	0.9978	1.0126	1.0782	1.2638	1.1724

ACGT-flank	TCCACGTGGA	0.9551	1.1764	0.9805	1.3048	1.0654	1.2274
ACGT-flank	TCCACGTGGC	1.8262	1.8431	1.8040	2.2634	2.2232	2.0367
ACGT-flank	TCCACGTGGG	1.8167	1.8367	1.8794	2.0214	1.9380	1.9413
ACGT-flank	TCCACGTGGT	1.2224	1.2648	1.2157	1.2696	1.2073	1.2194
ACGT-flank	TCCACGTGTA	1.0870	1.6741	1.5577	1.6969	1.7552	1.5868
ACGT-flank	TCCACGTGTC	1.7753	1.7687	2.0657	2.2093	1.8991	1.7751
ACGT-flank	TCCACGTGTG	1.4776	1.4542	1.2834	1.2940	1.4083	1.4410
ACGT-flank	TCCACGTTCC	0.8604	0.8003	1.3195	0.9058	0.9427	0.9388
ACGT-flank	TCCACGTTCG	0.5720	0.7674	0.8292	0.6988	0.6841	0.8748
ACGT-flank	TCCACGTTGA	0.8906	0.7845	1.0816	0.8622	0.8654	0.8404
ACGT-flank	TCCACGTTGC	0.7929	0.9789	1.3424	1.1786	1.0984	0.9015
ACGT-flank	TCCACGTTGG	1.1265	1.1774	1.2549	1.3024	1.1414	1.0341
ACGT-flank	TCGACGTAAA	0.5691	0.8733	0.6119	0.7623	0.7221	0.6276
ACGT-flank	TCGACGTAAC	0.8548	1.0011	0.9876	0.8933	0.7476	0.7629
ACGT-flank	TCGACGTAAG	0.8910	0.7961	0.8179	0.8829	0.8705	0.8521
ACGT-flank	TCGACGTACA	0.8957	0.7465	0.8532	0.6640	0.7346	0.8086
ACGT-flank	TCGACGTACC	1.0448	0.9567	1.1571	0.9552	1.0982	1.0913
ACGT-flank	TCGACGTACG	1.0104	0.8536	0.7891	0.7560	0.8725	0.8708
ACGT-flank	TCGACGTACT	0.7457	1.0631	0.9287	0.7733	0.8142	0.8725
ACGT-flank	TCGACGTAGA	0.8890	1.3463	1.0528	1.1608	1.0559	1.2151
ACGT-flank	TCGACGTAGC	0.8982	0.8319	0.7596	0.8171	0.7980	0.8606
ACGT-flank	TCGACGTAGG	1.0288	1.0971	1.0605	0.9952	1.1789	1.1916
ACGT-flank	TCGACGTAGT	0.9617	0.6836	0.9567	0.8241	0.9485	0.8141
ACGT-flank	TCGACGTATC	0.7555	0.6888	0.8671	0.5994	0.8655	0.7679
ACGT-flank	TCGACGTATG	0.8824	1.1590	0.9814	0.8710	1.0710	1.0554
ACGT-flank	TCGACGTCAA	0.4194	0.5375	0.4336	0.4861	0.4280	0.4039
ACGT-flank	TCGACGTCAC	1.0975	0.5359	1.1957	1.2595	1.1660	0.8720
ACGT-flank	TCGACGTCAG	0.7970	0.8373	0.8182	0.8831	0.8489	0.8143
ACGT-flank	TCGACGTCAT	0.8182	0.7193	0.6612	0.8756	0.7563	0.9596
ACGT-flank	TCGACGTCCA	1.0203	0.8898	0.9350	0.9000	0.8066	0.8628
ACGT-flank	TCGACGTCCC	0.9606	1.2123	0.7642	0.5690	0.7169	0.8224
ACGT-flank	TCGACGTCCG	0.7966	0.6985	0.8452	0.7464	0.7338	0.7872
ACGT-flank	TCGACGTCCT	0.9099	0.7121	1.0049	0.7822	0.7733	0.7174
ACGT-flank	TCGACGTCGA	0.8211	0.9708	0.9543	1.0193	0.8728	0.8787
ACGT-flank	TCGACGTCGC	0.6475	0.6730	0.8189	0.8583	0.6265	0.6001
ACGT-flank	TCGACGTCGG	0.8789	0.8829	0.8546	0.8838	0.9219	0.9927
ACGT-flank	TCGACGTCGT	0.8389	0.7291	0.9402	0.8525	0.9217	0.9802
ACGT-flank	TCGACGTCTA	0.6432	0.6933	0.7329	0.8683	0.7584	0.8851
ACGT-flank	TCGACGTCTC	0.9270	0.8949	0.6894	0.7556	0.9065	0.9322
ACGT-flank	TCGACGTCTG	1.0535	1.0292	0.9921	0.9810	1.0418	1.0921
ACGT-flank	TCGACGTGAA	1.0100	0.9357	0.9846	0.8255	0.8397	0.9634
ACGT-flank	TCGACGTGAC	0.8786	1.0126	0.8504	0.8162	0.8794	0.8157
ACGT-flank	TCGACGTGAG	0.8120	0.7329	0.7957	0.8121	0.7486	0.7932
ACGT-flank	TCGACGTGAT	1.0107	0.7818	0.6727	0.8254	0.7318	0.8342

ACGT-flank	TCGACGTGCA	0.9481	0.8713	0.9215	0.8009	1.1062	1.0209
ACGT-flank	TCGACGTGCC	0.8564	1.1036	0.8064	0.7306	0.8399	0.8312
ACGT-flank	TCGACGTGCG	0.8327	0.9466	0.8213	0.7244	0.8514	0.9433
ACGT-flank	TCGACGTGCT	0.8158	0.8228	0.8452	0.9308	1.0139	0.7953
ACGT-flank	TCGACGTGGA	1.1289	1.0090	1.0875	1.1614	0.9855	1.0495
ACGT-flank	TCGACGTGGC	1.0846	0.9908	1.0442	1.0349	1.0487	1.0409
ACGT-flank	TCGACGTGGG	1.3406	1.3324	1.2491	1.4645	1.3443	1.3756
ACGT-flank	TCGACGTGGT	0.9954	0.9597	1.1073	0.9144	1.0092	0.8823
ACGT-flank	TCGACGTGTA	1.3021	0.8543	1.1945	0.9249	1.0883	1.0502
ACGT-flank	TCGACGTGTC	1.5717	1.9622	1.9457	1.6704	1.6580	1.7144
ACGT-flank	TCGACGTGTG	1.0511	1.1245	1.0608	1.1661	1.3145	1.2062
ACGT-flank	TCGACGTGTT	0.9539	1.0770	1.0150	1.1742	1.0906	1.0429
ACGT-flank	TCGACGTTAC	1.2051	1.0955	1.3692	0.9259	1.2312	1.1232
ACGT-flank	TCGACGTTAG	0.9792	1.0645	0.9443	0.8739	0.8934	0.9314
ACGT-flank	TCGACGTTCC	0.7487	0.7084	0.9342	0.5378	0.6126	0.7346
ACGT-flank	TCGACGTTCG	0.7261	0.7542	0.8049	0.7938	0.8001	0.7905
ACGT-flank	TCGACGTTGA	0.9939	0.8665	0.8834	0.7748	0.9555	0.9309
ACGT-flank	TCGACGTTGC	0.7647	0.9198	0.7163	1.0975	0.7931	0.8822
ACGT-flank	TCGACGTTGG	0.8915	0.8835	0.8719	0.9484	0.9170	0.8865
ACGT-flank	TCGACGTTGT	0.3938	0.5979	0.6027	0.8326	0.7668	0.6440
ACGT-flank	TCGACGTTTG	1.2258	0.9436	1.4478	0.7660	1.0148	1.1077
ACGT-flank	TCTACGTAAG	0.7391	1.2339	1.3245	1.0223	0.7750	0.9059
ACGT-flank	TCTACGTACC	0.8774	0.6257	0.8441	0.6895	0.7259	0.7603
ACGT-flank	TCTACGTAGA	1.0454	0.8345	1.1125	0.8015	0.7490	0.9462
ACGT-flank	TCTACGTAGC	1.1336	1.1879	1.2479	1.2502	1.1154	1.0929
ACGT-flank	TCTACGTAGG	0.9012	0.7020	0.8594	1.0745	0.9181	0.7394
ACGT-flank	TCTACGTCCC	0.9321	0.8104	1.0470	0.8778	0.8832	0.9860
ACGT-flank	TCTACGTCCG	0.4454	0.3715	0.7059	0.7561	0.5136	0.5713
ACGT-flank	TCTACGTCCT	0.9839	0.9298	0.8911	0.7965	0.9191	0.9690
ACGT-flank	TCTACGTCGC	1.1193	1.1413	0.8667	0.8632	1.0506	1.0727
ACGT-flank	TCTACGTCGG	0.9421	0.8622	0.9989	0.9952	0.9678	0.9012
ACGT-flank	TCTACGTGAC	0.8164	0.9365	1.0106	0.8322	0.7410	0.8128
ACGT-flank	TCTACGTGAG	1.1072	0.9292	1.5544	1.1755	1.3597	1.1306
ACGT-flank	TCTACGTGCA	0.8166	0.8280	0.8911	0.9525	0.7199	0.9889
ACGT-flank	TCTACGTGCC	0.8581	0.8534	0.8941	0.8968	0.9473	0.9901
ACGT-flank	TCTACGTGCG	0.9513	0.8603	0.8977	0.9979	0.9208	0.9694
ACGT-flank	TCTACGTGGA	0.7990	0.6993	0.8574	0.7772	0.8450	0.8614
ACGT-flank	TCTACGTGGC	1.1557	1.0684	0.9477	1.4776	1.4077	1.2283
ACGT-flank	TCTACGTGGG	1.6347	1.5723	1.6851	1.8961	1.7058	1.6030
ACGT-flank	TCTACGTGTG	1.5827	1.4526	1.7360	1.7275	1.8623	1.5764
ACGT-flank	TCTACGTTGC	0.8879	0.6104	1.0437	1.0113	1.0644	0.8842
ACGT-flank	TCTACGTTGG	1.0698	1.3218	1.5012	0.8892	0.9152	0.8604
ACGT-flank	TGAACGTAAA	0.7575	1.3080	0.8941	0.8827	0.9836	1.1272
ACGT-flank	TGAACGTAAG	0.8432	0.9390	0.9177	0.7665	0.6622	0.8284

ACGT-flank	TGAACGTACA	1.2481	1.1918	1.2352	0.8663	1.1151	0.9826
ACGT-flank	TGAACGTACC	0.7441	0.7706	0.4358	0.7669	0.5603	0.7763
ACGT-flank	TGAACGTACG	0.9034	0.9590	0.9920	0.9241	1.0140	0.8745
ACGT-flank	TGAACGTAGA	0.7448	0.7707	0.6069	0.9412	0.6182	0.7136
ACGT-flank	TGAACGTAGC	0.9405	0.9123	1.1206	1.0844	1.0567	1.1214
ACGT-flank	TGAACGTAGG	1.1990	1.1969	1.1658	1.0512	1.0893	1.1523
ACGT-flank	TGAACGTAGT	0.7382	0.8167	0.7873	0.9060	0.9127	0.8130
ACGT-flank	TGAACGTATG	0.8322	0.6408	0.8897	0.9044	0.8161	0.7775
ACGT-flank	TGAACGTCAA	0.8762	0.6808	0.8595	0.7906	0.7970	0.8808
ACGT-flank	TGAACGTCAC	1.3050	0.9379	1.3212	1.1726	1.1984	1.0759
ACGT-flank	TGAACGTCAG	0.8546	0.8420	0.6492	0.9466	0.6868	0.6846
ACGT-flank	TGAACGTCCC	0.8371	0.8959	0.9614	0.9143	0.8711	0.9990
ACGT-flank	TGAACGTCCG	0.7612	0.7321	0.6467	0.7458	0.7381	0.8172
ACGT-flank	TGAACGTCCT	0.5946	0.7957	0.6300	0.6717	0.7430	0.7426
ACGT-flank	TGAACGTCGA	0.7310	0.7678	1.0146	0.7565	0.8009	0.8304
ACGT-flank	TGAACGTCGC	0.9508	0.7434	1.1093	0.9858	1.0082	0.9508
ACGT-flank	TGAACGTCGG	1.0072	0.7782	0.8234	0.8447	0.8609	0.8675
ACGT-flank	TGAACGTCGT	0.7422	0.5313	0.8120	0.7770	0.7280	0.9325
ACGT-flank	TGAACGTCTA	0.6063	0.7896	0.8026	0.6334	0.6154	0.7103
ACGT-flank	TGAACGTCTC	0.7948	0.9615	1.0355	0.8885	0.9226	0.8884
ACGT-flank	TGAACGTCTG	0.6796	0.7910	0.5642	0.4709	1.0513	0.8349
ACGT-flank	TGAACGTGAA	0.6059	0.6011	0.6840	0.7417	0.7054	0.6836
ACGT-flank	TGAACGTGAC	0.8664	0.7018	0.8945	0.8106	0.8698	0.8253
ACGT-flank	TGAACGTGAG	0.8944	0.7831	0.8232	0.9360	0.9072	0.8902
ACGT-flank	TGAACGTGCA	1.1220	0.7471	1.1306	0.8826	0.9859	1.0751
ACGT-flank	TGAACGTGCG	0.9751	0.8876	0.8480	0.9519	1.0324	0.9894
ACGT-flank	TGAACGTGCT	0.9571	0.8343	0.8920	0.8301	0.9015	0.8661
ACGT-flank	TGAACGTGGA	1.0378	0.9450	0.8936	0.9126	0.9236	0.9253
ACGT-flank	TGAACGTGGC	0.7135	0.6841	0.6285	0.8060	0.7761	0.8610
ACGT-flank	TGAACGTGGG	1.2207	1.3529	1.2219	1.2391	1.2809	1.2574
ACGT-flank	TGAACGTGGT	0.8824	0.8693	0.8682	0.9082	0.7994	0.8140
ACGT-flank	TGAACGTGTA	0.6178	0.9754	0.9348	0.7811	0.7294	0.9400
ACGT-flank	TGAACGTGTC	1.2301	1.0174	1.0317	0.9395	0.9090	0.8843
ACGT-flank	TGAACGTGTG	1.2807	1.3964	1.2973	1.1105	1.2253	1.2204
ACGT-flank	TGAACGTTAG	1.3530	1.6240	1.3425	1.0136	1.1820	1.5098
ACGT-flank	TGAACGTTCG	0.9719	1.1415	1.0820	0.7860	1.0442	0.9888
ACGT-flank	TGAACGTTGA	1.0540	0.7238	1.0196	0.7361	0.9704	0.9626
ACGT-flank	TGAACGTTGC	0.6902	0.7334	0.6668	0.8845	0.6113	0.7654
ACGT-flank	TGAACGTTGG	1.2996	1.1431	1.0153	0.9788	1.0757	1.1022
ACGT-flank	TGCACGTAAC	0.7181	0.6628	0.8334	0.8442	0.9038	1.1445
ACGT-flank	TGCACGTAAG	0.8572	0.8223	0.9649	0.8706	0.7186	0.8754
ACGT-flank	TGCACGTACC	0.6998	0.7563	0.8032	0.8080	0.8674	0.8383
ACGT-flank	TGCACGTACG	0.7358	0.8536	0.7001	0.9461	0.7879	0.6860
ACGT-flank	TGCACGTACT	0.8844	0.7914	0.9198	1.0266	0.9459	0.9351

ACGT-flank	TGCACGTAGA	0.7397	0.6255	0.9958	0.6656	0.6912	0.7564
ACGT-flank	TGCACGTAGC	0.7410	0.6696	0.7273	0.6894	0.6804	0.7727
ACGT-flank	TGCACGTAGG	1.1211	0.9621	1.1197	1.0814	0.9882	1.0831
ACGT-flank	TGCACGTAGT	0.8916	0.9272	0.8308	1.0075	0.8305	0.9318
ACGT-flank	TGCACGTATC	1.6228	1.2116	0.8865	0.7969	1.3041	1.5649
ACGT-flank	TGCACGTATG	0.8586	0.6509	0.9682	0.7137	0.8422	0.8449
ACGT-flank	TGCACGTCAA	1.0278	0.5621	0.6909	0.5631	0.7480	0.7799
ACGT-flank	TGCACGTCAG	0.9445	1.2106	1.0945	1.0005	0.8925	1.0052
ACGT-flank	TGCACGTCCA	0.9668	1.0729	0.8223	1.0147	1.2265	1.2201
ACGT-flank	TGCACGTCCC	0.8018	0.8098	0.8493	0.6824	0.8480	0.8713
ACGT-flank	TGCACGTCCG	0.8147	1.0941	0.7859	0.9173	0.8632	0.8294
ACGT-flank	TGCACGTCCT	0.8189	1.0085	0.8632	0.8118	0.6495	0.8426
ACGT-flank	TGCACGTCGA	0.7133	0.7658	0.6321	0.7515	0.6845	0.7799
ACGT-flank	TGCACGTCGC	0.6593	0.6526	0.7147	0.8728	0.7125	0.7034
ACGT-flank	TGCACGTCGG	0.8622	0.8331	0.7469	0.7889	0.7366	0.7521
ACGT-flank	TGCACGTCGT	0.9451	0.9404	0.8466	0.7484	0.9103	0.8663
ACGT-flank	TGCACGTCTC	0.8398	1.0349	0.5853	0.8138	0.8322	0.7615
ACGT-flank	TGCACGTCTG	0.7844	1.0153	0.8127	0.8461	0.9293	0.8789
ACGT-flank	TGCACGTGAA	0.6548	0.6141	0.9116	0.7539	0.8757	0.9252
ACGT-flank	TGCACGTGAC	0.8158	0.9753	0.8626	0.7030	0.7356	0.8778
ACGT-flank	TGCACGTGAG	0.8060	0.7134	0.7542	0.7341	0.6443	0.7077
ACGT-flank	TGCACGTGAT	1.0032	0.8907	0.7408	0.8554	0.8538	0.8563
ACGT-flank	TGCACGTGCA	1.0653	1.1685	1.0728	1.0141	1.0295	1.0822
ACGT-flank	TGCACGTGCC	0.6146	0.9228	0.4821	0.7256	0.8083	0.8485
ACGT-flank	TGCACGTGCG	0.8229	0.9166	0.9271	1.0155	0.9078	0.8084
ACGT-flank	TGCACGTGCT	1.2174	1.1449	1.4562	1.0436	1.2691	1.1273
ACGT-flank	TGCACGTGGA	0.8860	0.8359	1.0230	1.1950	1.1163	0.9823
ACGT-flank	TGCACGTGGC	1.3371	1.2913	1.3218	1.5482	1.3884	1.3974
ACGT-flank	TGCACGTGGG	1.2668	1.3023	1.2202	1.3743	1.3058	1.2906
ACGT-flank	TGCACGTGGT	0.9419	0.9421	0.9761	1.0023	0.9289	0.9962
ACGT-flank	TGCACGTGTC	0.9283	0.7974	0.6278	0.9433	0.8962	0.8317
ACGT-flank	TGCACGTGTG	1.0157	1.0970	1.1689	1.0422	1.1535	1.0141
ACGT-flank	TGCACGTGTT	1.3488	1.0883	1.3324	1.4820	1.3336	1.3819
ACGT-flank	TGCACGTTAG	0.7958	0.7816	0.8668	0.5942	0.6910	0.6836
ACGT-flank	TGCACGTTCC	0.9375	1.1629	0.8129	0.7208	0.8514	0.8739
ACGT-flank	TGCACGTTCG	0.9199	0.8971	0.7904	0.8465	0.8743	1.1044
ACGT-flank	TGCACGTTGA	1.2462	0.9637	1.2286	1.0716	1.1692	0.9862
ACGT-flank	TGCACGTTGC	1.0695	0.9408	0.9468	1.0088	1.0904	0.9781
ACGT-flank	TGCACGTTGG	1.0105	0.9907	1.0111	0.9836	0.9059	0.9859
ACGT-flank	TGCACGTTGT	0.8561	0.8242	0.8076	0.8931	0.7847	0.7076
ACGT-flank	TGGACGTAAA	0.6464	0.7171	0.6761	0.7992	0.7920	0.7703
ACGT-flank	TGGACGTAAC	0.8103	0.7532	0.6190	0.7319	0.8576	0.7951
ACGT-flank	TGGACGTAAG	0.9337	1.0918	0.9468	0.8980	0.9432	0.9542
ACGT-flank	TGGACGTAAT	0.6487	0.7016	1.0767	1.1203	0.8629	0.6863

ACGT-flank	TGGACGTACA	0.7197	0.7999	0.6911	0.9467	0.8050	0.8451
ACGT-flank	TGGACGTACC	0.4787	0.7167	0.8767	0.7697	0.4874	0.6191
ACGT-flank	TGGACGTACG	0.8014	0.7627	0.7802	0.7323	0.7823	0.7606
ACGT-flank	TGGACGTACT	0.7591	0.8076	0.8646	0.8378	0.7906	0.7886
ACGT-flank	TGGACGTAGA	0.8165	0.8624	0.8185	0.7630	0.8071	0.8392
ACGT-flank	TGGACGTAGC	0.8267	0.9685	0.9830	0.8086	0.9559	0.9293
ACGT-flank	TGGACGTAGG	1.1726	1.1039	1.1321	1.1721	1.2184	1.1526
ACGT-flank	TGGACGTAGT	0.9597	0.9008	0.8974	1.0059	0.8942	0.9070
ACGT-flank	TGGACGTATA	1.1919	0.6869	0.9337	0.9773	1.0860	1.0441
ACGT-flank	TGGACGTATC	0.6829	0.8793	1.0149	0.8487	0.8307	0.9046
ACGT-flank	TGGACGTATG	1.0028	1.1369	0.9159	1.0570	0.9598	1.0440
ACGT-flank	TGGACGTCAA	0.7558	0.7362	0.6009	0.8977	0.6416	0.8455
ACGT-flank	TGGACGTCAC	0.8455	0.8540	0.7475	0.7799	0.9196	0.8279
ACGT-flank	TGGACGTCAG	0.7864	0.7696	0.8582	0.8300	0.7586	0.7814
ACGT-flank	TGGACGTCAT	0.6485	0.7587	0.8904	0.8870	0.6100	0.7710
ACGT-flank	TGGACGTCCA	0.9817	0.8121	0.8022	0.8450	0.7884	0.9544
ACGT-flank	TGGACGTCCC	0.7180	0.8599	0.7439	0.6530	0.9221	0.9149
ACGT-flank	TGGACGTCCG	0.9190	0.9041	0.9634	0.8907	0.9114	0.8353
ACGT-flank	TGGACGTCCT	0.9144	0.8666	0.7714	0.8910	0.7995	0.8750
ACGT-flank	TGGACGTCGA	0.9644	0.9173	0.7717	0.8794	0.8195	0.8086
ACGT-flank	TGGACGTCGC	0.8855	0.8672	0.9528	0.8338	0.8348	0.9184
ACGT-flank	TGGACGTCGG	1.0943	1.1595	1.0875	1.0430	1.1123	1.1356
ACGT-flank	TGGACGTCGT	0.9389	0.9381	0.8285	0.9456	0.8788	0.8788
ACGT-flank	TGGACGTCTA	0.8718	0.8639	0.9818	0.8731	0.9989	0.9391
ACGT-flank	TGGACGTCTC	0.8525	0.9681	0.8778	0.8024	0.8613	0.9865
ACGT-flank	TGGACGTCTG	0.8061	0.8448	0.7865	0.7552	0.7559	0.7607
ACGT-flank	TGGACGTGAA	0.8094	0.7110	1.0166	0.7482	0.6870	0.8659
ACGT-flank	TGGACGTGAC	0.7716	0.8262	0.8836	0.8535	0.8832	0.7884
ACGT-flank	TGGACGTGAG	0.7730	0.8198	0.7836	0.9559	0.8038	0.8343
ACGT-flank	TGGACGTGAT	0.7305	0.7609	0.9427	0.9147	0.8257	0.7751
ACGT-flank	TGGACGTGCA	0.9139	0.9036	0.8934	0.8709	0.9991	0.8645
ACGT-flank	TGGACGTGCC	0.6752	0.8586	0.5298	0.5998	0.7274	0.6952
ACGT-flank	TGGACGTGCG	0.8260	0.8197	0.7574	0.8891	0.8067	0.8422
ACGT-flank	TGGACGTGCT	0.8539	0.9366	0.9179	0.7891	0.8145	0.8505
ACGT-flank	TGGACGTGGA	0.7748	0.7704	0.8139	0.9609	0.8890	0.8245
ACGT-flank	TGGACGTGGC	1.3511	1.2296	1.2886	1.1443	1.2070	1.2261
ACGT-flank	TGGACGTGGG	1.5063	1.3838	1.3673	1.5533	1.4408	1.4814
ACGT-flank	TGGACGTGGT	0.9600	0.8723	0.8798	0.8708	0.9169	0.9317
ACGT-flank	TGGACGTGTA	1.0749	1.3238	1.0285	1.2094	1.0663	1.0233
ACGT-flank	TGGACGTGTC	0.8621	0.9429	0.9404	0.8563	1.0463	0.8437
ACGT-flank	TGGACGTGTG	1.1786	1.0728	1.1260	1.0470	1.0907	1.1347
ACGT-flank	TGGACGTGTT	1.4175	1.4118	1.7298	1.6772	1.7151	1.5742
ACGT-flank	TGGACGTTAA	0.6527	0.6047	0.6164	0.7929	0.8053	0.9560
ACGT-flank	TGGACGTTAC	0.7484	0.9926	0.6967	0.7647	0.7735	0.8139

ACGT-flank	TGGACGTTAG	0.5555	0.9221	0.9246	0.7946	0.8007	0.8161
ACGT-flank	TGGACGTTCA	0.9003	0.6926	0.7655	1.2210	1.0149	0.8095
ACGT-flank	TGGACGTTCC	0.7925	0.7702	0.8125	0.7263	0.8304	0.8368
ACGT-flank	TGGACGTTCG	0.7548	0.7408	0.7309	0.6839	0.7100	0.7192
ACGT-flank	TGGACGTTGA	1.0315	0.9407	0.8687	0.7781	0.8038	0.7878
ACGT-flank	TGGACGTTGC	1.0221	0.8850	0.8456	0.8810	0.9591	0.9902
ACGT-flank	TGGACGTTGG	1.1751	1.1995	1.1495	1.1075	1.0886	1.1265
ACGT-flank	TGGACGTTGT	0.7783	0.8249	0.8155	0.6240	0.7098	0.7910
ACGT-flank	TGGACGTTTG	0.7278	0.9501	1.0096	0.8066	0.8003	0.8488
ACGT-flank	TGTACGTAAG	0.6704	0.9833	1.2372	0.5564	0.8884	0.8302
ACGT-flank	TGTACGTACC	0.5859	0.7300	0.6463	0.7753	0.7430	0.6987
ACGT-flank	TGTACGTACG	0.6605	0.7575	0.8652	0.7613	0.8475	0.8449
ACGT-flank	TGTACGTAGC	0.9337	0.7562	0.7394	0.9367	0.6893	0.8024
ACGT-flank	TGTACGTAGG	1.0309	1.2664	1.2889	1.2364	1.0983	1.1261
ACGT-flank	TGTACGTCAA	0.8947	0.8913	0.7821	0.9540	1.0109	0.9049
ACGT-flank	TGTACGTCAG	1.2665	1.3135	1.3462	1.1457	1.1428	1.2169
ACGT-flank	TGTACGTCCA	0.8652	0.5527	0.8705	0.7435	0.6317	0.6248
ACGT-flank	TGTACGTCCC	0.8712	0.8720	0.8140	0.8734	0.9247	1.0779
ACGT-flank	TGTACGTCCG	1.1715	1.1939	1.3504	1.2288	1.2241	1.2569
ACGT-flank	TGTACGTCGA	0.7461	0.7262	0.7820	0.7488	0.7085	0.7166
ACGT-flank	TGTACGTCGC	0.7826	0.7317	0.7427	0.8020	0.6569	0.7318
ACGT-flank	TGTACGTCGG	0.7971	0.9121	0.8823	0.8049	0.7903	0.8579
ACGT-flank	TGTACGTCGT	0.7312	0.8559	0.7949	0.7534	0.9456	1.0319
ACGT-flank	TGTACGTCTG	0.4511	0.6615	0.8397	0.6740	0.9724	1.2005
ACGT-flank	TGTACGTGAC	0.6577	0.7982	0.6598	0.7089	0.6036	0.6056
ACGT-flank	TGTACGTGAG	0.9441	0.9981	0.9953	0.9612	0.9244	1.0723
ACGT-flank	TGTACGTGCA	0.9797	1.0449	1.1896	0.9875	1.1750	1.0633
ACGT-flank	TGTACGTGCC	1.0427	1.1312	1.0852	0.8188	1.0280	1.0663
ACGT-flank	TGTACGTGCG	0.9521	0.9250	0.8679	0.8500	0.9122	1.0126
ACGT-flank	TGTACGTGGA	0.8729	0.9479	1.0295	0.8733	0.8184	0.9008
ACGT-flank	TGTACGTGGC	1.1559	1.0759	1.1449	1.0866	1.2110	1.1479
ACGT-flank	TGTACGTGGG	1.3182	1.4058	1.3366	1.3946	1.3504	1.3255
ACGT-flank	TGTACGTGGT	1.1498	1.0381	1.0178	0.8776	1.2076	1.0013
ACGT-flank	TGTACGTGTA	1.2584	0.6182	0.9013	0.6281	0.9167	1.0296
ACGT-flank	TGTACGTGTC	0.8241	0.8832	0.9413	1.0272	0.9595	0.7679
ACGT-flank	TGTACGTGTG	1.1090	1.1430	1.0297	1.1173	1.2017	1.1471
ACGT-flank	TGTACGTTGC	0.8423	0.9081	0.8942	0.9204	0.7303	0.9063
ACGT-flank	TGTACGTTGG	0.9599	1.0953	0.9543	0.8382	0.9201	0.8583
ACGT-flank	TTAACGTAGG	0.9429	0.6203	1.0536	0.7929	0.9218	0.8069
ACGT-flank	TTAACGTCGG	0.6834	0.8935	0.8003	0.5892	0.8828	1.0205
ACGT-flank	TTAACGTGCC	0.8369	0.8459	1.0696	0.8302	0.7374	0.8634
ACGT-flank	TTAACGTGGC	0.9596	0.7119	0.7025	0.9275	0.8296	0.9594
ACGT-flank	TTAACGTGGG	1.5655	0.9719	1.1538	1.1273	1.2438	1.0430
ACGT-flank	TTCACGTAGG	0.8486	1.1586	0.4472	0.6691	0.8111	0.8904

ACGT-flank	TTCACGTCGG	0.7910	1.0426	0.8638	0.7849	0.6048	0.6770
ACGT-flank	TTCACGTGCC	0.6517	0.7486	0.4507	0.8618	0.5943	0.7283
ACGT-flank	TTCACGTGCG	1.0356	1.0026	0.9682	1.0888	1.1698	0.9166
ACGT-flank	TTCACGTGGC	0.9387	0.9362	0.9443	1.0609	0.9841	0.8787
ACGT-flank	TTCACGTGGG	1.2805	1.4656	1.4189	1.1600	1.4332	1.3232
ACGT-flank	TTGACGTAAG	0.8028	0.8042	1.1721	0.9603	0.9076	0.9971
ACGT-flank	TTGACGTACC	1.0661	0.9563	1.1209	1.1725	1.1146	1.0055
ACGT-flank	TTGACGTAGA	1.1122	1.3681	1.2291	1.1964	0.8010	1.0933
ACGT-flank	TTGACGTAGC	1.0308	1.0062	0.9237	1.3268	0.9988	1.0449
ACGT-flank	TTGACGTAGG	1.2864	1.2411	1.2092	1.1381	1.0484	1.0891
ACGT-flank	TTGACGTATG	0.7679	0.9383	0.6961	0.7368	0.6690	0.8927
ACGT-flank	TTGACGTCAG	1.2546	0.9266	1.0279	1.3016	1.4016	1.0087
ACGT-flank	TTGACGTCCA	1.0413	1.0774	1.2114	1.1596	1.0618	0.9973
ACGT-flank	TTGACGTCCC	1.2598	1.1128	0.9442	1.1495	1.1192	1.1520
ACGT-flank	TTGACGTCCG	1.0602	1.0118	1.0744	1.0589	1.0834	1.1147
ACGT-flank	TTGACGTCGA	0.8274	0.9096	1.1001	0.8418	0.9416	0.8537
ACGT-flank	TTGACGTCGC	0.9763	1.1819	1.0265	0.8895	1.0105	1.0442
ACGT-flank	TTGACGTCGG	1.1890	1.0783	1.1335	1.0466	1.1299	1.1294
ACGT-flank	TTGACGTCTG	1.2323	1.2392	1.3477	1.2619	1.2706	1.2749
ACGT-flank	TTGACGTGAA	0.9876	0.8699	1.2743	1.0247	1.0095	0.8961
ACGT-flank	TTGACGTGAC	1.2134	1.2391	1.3245	1.2793	1.2928	1.1591
ACGT-flank	TTGACGTGAG	1.0654	1.0468	1.2744	1.1961	1.1482	1.0729
ACGT-flank	TTGACGTGCA	1.5316	1.1990	1.3606	1.3153	1.3603	1.1938
ACGT-flank	TTGACGTGCC	1.0152	0.9606	1.0553	0.8802	0.9934	1.0758
ACGT-flank	TTGACGTGCG	0.9750	0.9833	1.0374	1.1252	1.0192	1.0068
ACGT-flank	TTGACGTGGA	1.0632	1.3145	1.2480	1.2919	1.2857	1.2067
ACGT-flank	TTGACGTGGC	1.2280	1.2892	1.2574	1.4327	1.2286	1.1697
ACGT-flank	TTGACGTGGG	1.4280	1.3368	1.5520	1.5561	1.4997	1.3992
ACGT-flank	TTGACGTGGT	0.8612	1.0726	1.1263	1.0081	1.0594	1.0586
ACGT-flank	TTGACGTGTC	0.7942	1.2192	1.3835	1.6500	1.4045	1.4612
ACGT-flank	TTGACGTGTG	1.3810	1.4278	1.1607	1.5089	1.5036	1.3343
ACGT-flank	TTGACGTTCG	1.3195	0.7878	0.8577	0.8871	0.9888	1.1414
ACGT-flank	TTGACGTTGG	1.3690	1.3996	1.2010	1.2645	1.4361	1.2797
ACGT-flank	ACAACGTAAC	0.7938	0.9720	0.8191	0.7461	0.7042	0.9020
ACGT-flank	AGGACGTTTC	1.1017	1.1257	1.1382	1.1642	1.2802	1.0855
ACGT-flank	AGTACGTTGC	0.8594	0.7687	0.8402	0.7108	0.7536	1.0014
ACGT-flank	CCCACGTGCT	0.9570	1.1900	0.9896	1.1183	1.1733	1.0217
ACGT-flank	CCTACGTGCA	1.1788	0.8081	0.9830	0.8677	0.9567	0.9641
ACGT-flank	CTAACGTTCG	0.8140	0.7794	0.9737	0.6497	0.9199	0.8304
ACGT-flank	CTCACGTATC	0.9408	0.7028	0.9534	0.6901	0.6222	0.6474
ACGT-flank	GTTACGTTGG	1.0103	0.6759	0.9330	0.7347	0.7103	0.7684
ACGT-flank	TCAACGTGTA	1.3863	1.5008	1.4319	1.7382	1.5057	1.3810
ACGT-flank	AAAACGTAAC	0.8752	0.8790	0.9032	0.6910	0.7601	0.7457
ACGT-flank	CATACGTCCA	0.8432	0.7350	1.0352	1.1328	0.7686	0.8190

ACGT-flank	CGGACGTGCA	0.7363	0.9693	0.8942	0.7884	0.7437	0.8901
ACGT-flank	CTAACGTTAG	1.0858	1.3690	0.9682	0.9350	0.9241	0.8684
ACGT-flank	GACACGTCTT	1.0248	1.2820	1.0249	1.2987	1.3099	1.2213
ACGT-flank	GCGACGTAGC	0.6446	0.6749	0.8889	0.6623	0.5356	0.7035
ACGT-flank	TTGACGTACG	0.9848	0.6892	1.0354	0.8451	0.8432	0.8860

Table S4. Identification of ABA-responsive ABRE variants.

Library	ABREs	log_2(FoldChange)	P-value	Effect
ACGT-core	TCTATGAGTC	1.3188	0.0002	Enhancing
ACGT-core	TCTTCTAGTC	1.2740	0.0008	Enhancing
ACGT-core	TCTAGAAGTC	1.0900	0.0164	Enhancing
ACGT-core	TCTAGCTGTC	0.9419	0.0093	Enhancing
ACGT-core	TCTCACTGTC	0.8270	0.0020	Enhancing
ACGT-core	TCTCACGGTC	0.6356	0.0215	Enhancing
ACGT-core	TCTTCCTGTC	0.5790	0.0074	Enhancing
ACGT-core	TCTAGATGTC	0.5763	0.0129	Enhancing
ACGT-core	TCTCTCTGTC	0.5417	0.0079	Enhancing
ACGT-core	TCTGTACGTC	0.5095	0.0051	Enhancing
ACGT-core	TCTGTCGGTC	0.4796	0.0157	Enhancing
ACGT-core	TCTGGATGTC	0.4787	0.0119	Enhancing
ACGT-core	TCTGTCCGTC	0.4295	0.0473	Enhancing
ACGT-core	TCTATGCGTC	0.3950	0.0403	Enhancing
ACGT-core	TCTGTGTGTC	-0.3619	0.0439	Blunting
ACGT-core	TCTTTCGGTC	-0.3851	0.0176	Blunting
ACGT-core	TCTGACCGTC	-0.4213	0.0428	Blunting
ACGT-core	TCTGATAGTC	-0.4547	0.0369	Blunting
ACGT-core	TCTTAGAGTC	-0.4715	0.0433	Blunting
ACGT-core	TCTATGTGTC	-0.5974	0.0187	Blunting
ACGT-core	TCTACAGGTC	-0.6658	0.0344	Blunting
ACGT-core	TCTCACCGTC	-0.7294	0.0036	Blunting
ACGT-flank	GCGACGTAGA	0.8777	0.0009	Enhancing
ACGT-flank	ACAACGTCGT	0.8762	0.0009	Enhancing
ACGT-flank	TCAACGTAGA	0.8378	0.0185	Enhancing
ACGT-flank	ACGACGTCTA	0.6894	0.0064	Enhancing
ACGT-flank	TAAACGTGCT	0.6305	0.0048	Enhancing
ACGT-flank	AGAACGTCCC	0.6174	0.0349	Enhancing
ACGT-flank	ACCACGTCCA	0.5833	0.0023	Enhancing
ACGT-flank	GAGACGTGAA	0.5672	0.0019	Enhancing
ACGT-flank	TCCACGTGAC	0.4889	0.0031	Enhancing
ACGT-flank	TCAACGTTGC	0.4600	0.0063	Enhancing
ACGT-flank	TGGACGTTAA	0.4505	0.0015	Enhancing
ACGT-flank	GGCACGTGCC	0.4459	0.0250	Enhancing
ACGT-flank	ACGACGTACT	0.4397	0.0096	Enhancing
ACGT-flank	GACACGTGTC	0.4280	0.0000	Enhancing
ACGT-flank	CTGACGTGTT	0.4249	0.0114	Enhancing
ACGT-flank	TTGACGTGTC	0.4116	0.0386	Enhancing
ACGT-flank	AGTACGTCCT	0.4055	0.0155	Enhancing
ACGT-flank	TCAACGTGAG	0.3904	0.0016	Enhancing
ACGT-flank	TCTACGTGGC	0.3843	0.0008	Enhancing
ACGT-flank	TGCACGTAAC	0.3841	0.0298	Enhancing

ACGT-flank	TAGACGTACT	0.3788	0.0339	Enhancing
ACGT-flank	TGGACGTTCA	0.3772	0.0384	Enhancing
ACGT-flank	ATGACGTGTA	0.3714	0.0285	Enhancing
ACGT-flank	GACACGTGGT	0.3344	0.0000	Enhancing
ACGT-flank	CGCACGTCTT	0.3254	0.0288	Enhancing
ACGT-flank	AGAACGTGTG	0.3220	0.0020	Enhancing
ACGT-flank	GCGACGTCTG	0.3173	0.0105	Enhancing
ACGT-flank	TACACGTGGC	0.3167	0.0037	Enhancing
ACGT-flank	GCCACGTGGA	0.3116	0.0369	Enhancing
ACGT-flank	GCTACGTAAA	0.2997	0.0398	Enhancing
ACGT-flank	GCTACGTGGC	0.2963	0.0367	Enhancing
ACGT-flank	GACACGTGTA	0.2921	0.0020	Enhancing
ACGT-flank	CACACGTGCC	0.2878	0.0362	Enhancing
ACGT-flank	ACCACGTGTC	0.2872	0.0072	Enhancing
ACGT-flank	TGAACGTGGC	0.2764	0.0065	Enhancing
ACGT-flank	GCTACGTCCG	0.2719	0.0172	Enhancing
ACGT-flank	GGAACGTTCT	0.2700	0.0485	Enhancing
ACGT-flank	TGCACGTGGA	0.2667	0.0321	Enhancing
ACGT-flank	GACACGTGGC	0.2655	0.0030	Enhancing
ACGT-flank	GGCACGTCTT	0.2639	0.0303	Enhancing
ACGT-flank	AAAACGTAAC	-0.2699	0.0146	Blunting
ACGT-flank	AAAACGTCGG	-0.2718	0.0033	Blunting
ACGT-flank	GCCACGTTCG	-0.2759	0.0020	Blunting
ACGT-flank	GGTACGTGAT	-0.2759	0.0003	Blunting
ACGT-flank	CCGACGTCTA	-0.2786	0.0330	Blunting
ACGT-flank	CGCACGTCGT	-0.2818	0.0139	Blunting
ACGT-flank	TAGACGTCTG	-0.2825	0.0335	Blunting
ACGT-flank	GGGACGTCTA	-0.2981	0.0005	Blunting
ACGT-flank	AGAACGTCGA	-0.3022	0.0362	Blunting
ACGT-flank	TGAACGTACA	-0.3042	0.0158	Blunting
ACGT-flank	CAGACGTACT	-0.3050	0.0418	Blunting
ACGT-flank	TGCACGTTAG	-0.3089	0.0257	Blunting
ACGT-flank	GTGACGTCAA	-0.3109	0.0330	Blunting
ACGT-flank	CGTACGTCAC	-0.3110	0.0212	Blunting
ACGT-flank	GTAACGTGAA	-0.3147	0.0108	Blunting
ACGT-flank	ATGACGTTGA	-0.3162	0.0348	Blunting
ACGT-flank	CGTACGTCGA	-0.3169	0.0108	Blunting
ACGT-flank	GTGACGTACA	-0.3269	0.0018	Blunting
ACGT-flank	ACCACGTGAT	-0.3345	0.0115	Blunting
ACGT-flank	CTAACGTCCA	-0.3421	0.0046	Blunting
ACGT-flank	AACACGTGCG	-0.3434	0.0089	Blunting
ACGT-flank	TCGACGTTCC	-0.3441	0.0421	Blunting
ACGT-flank	GCAACGTAGA	-0.3627	0.0193	Blunting
ACGT-flank	AGTACGTACG	-0.3684	0.0019	Blunting

ACGT-flank	GAGACGTTAC	-0.3751	0.0346	Blunting
ACGT-flank	TTCACGTCGG	-0.3784	0.0467	Blunting
ACGT-flank	ACGACGTGGT	-0.3834	0.0027	Blunting
ACGT-flank	GAGACGTTCT	-0.3850	0.0104	Blunting
ACGT-flank	CAAACGTATG	-0.3877	0.0351	Blunting
ACGT-flank	GGTACGTATC	-0.3905	0.0263	Blunting
ACGT-flank	ACAACGTGCA	-0.3932	0.0047	Blunting
ACGT-flank	AGCACGTGTT	-0.3978	0.0149	Blunting
ACGT-flank	CTCACGTATC	-0.4036	0.0296	Blunting
ACGT-flank	GCGACGTAGT	-0.4070	0.0090	Blunting
ACGT-flank	CGCACGTAGA	-0.4071	0.0077	Blunting
ACGT-flank	AAAACGTCCA	-0.4094	0.0130	Blunting
ACGT-flank	GATACGTCGA	-0.4198	0.0463	Blunting
ACGT-flank	AGCACGTAAC	-0.4305	0.0272	Blunting
ACGT-flank	AGCACGTCCC	-0.4345	0.0263	Blunting
ACGT-flank	CTAACGTCGA	-0.4434	0.0082	Blunting
ACGT-flank	CAAACGTGCA	-0.4474	0.0114	Blunting
ACGT-flank	CCAACGTTGC	-0.4604	0.0169	Blunting
ACGT-flank	TCGACGTCCC	-0.4711	0.0438	Blunting
ACGT-flank	CAAACGTCCA	-0.4726	0.0403	Blunting
ACGT-flank	CTCACGTAAG	-0.4763	0.0102	Blunting
ACGT-flank	ACGACGTCCC	-0.4854	0.0321	Blunting
ACGT-flank	CCCACGTGAG	-0.5196	0.0170	Blunting
ACGT-flank	GAAACGTCTA	-0.5220	0.0173	Blunting
ACGT-flank	TCTACGTTGG	-0.5443	0.0029	Blunting
ACGT-flank	GCGACGTTTA	-0.5464	0.0009	Blunting
ACGT-flank	CAGACGTCGC	-0.6125	0.0431	Blunting
ACGT-flank	CATACGTAGC	-0.6386	0.0051	Blunting
ACGT-flank	ACCACGTATC	-0.6854	0.0005	Blunting
ACGT-flank	TACACGTCGA	-0.7160	0.0042	Blunting
